# Current and Future Niche of North and Central American Sand Flies (Diptera: Psychodidae) in Climate Change Scenarios

**DOI:** 10.1371/journal.pntd.0002421

**Published:** 2013-09-19

**Authors:** David Moo-Llanes, Carlos N. Ibarra-Cerdeña, Eduardo A. Rebollar-Téllez, Sergio Ibáñez-Bernal, Camila González, Janine M. Ramsey

**Affiliations:** 1 Centro Regional de Investigación en Salud Pública (CRISP), Instituto Nacional de Salud Pública (INSP), Tapachula, Chiapas, México; 2 Departamento de Ecología Humana, Centro de Investigaciones y Estudios Avanzados del Instituto Politécnico Nacional (CINVESTAV), Mérida, Yucatán, México; 3 Universidad Autónoma de Nuevo León (UANL), Facultad de Ciencias Biológicas, Zoología de Invertebrados, Cuidad Universitaria, San Nicolás de los Garzas, Nuevo León, México; 4 Red Ambiente y Sustentabilidad, Instituto de Ecología A.C. (INECOL), Veracruz, México; 5 Departamento de Ciencias Biológicas, Centro de Investigaciones en Microbiología y Parasitología Tropical (CIMPAT), Universidad de los Andes, Bogotá, Colombia; National Institutes of Health, United States of America

## Abstract

Ecological niche models are useful tools to infer potential spatial and temporal distributions in vector species and to measure epidemiological risk for infectious diseases such as the Leishmaniases. The ecological niche of 28 North and Central American sand fly species, including those with epidemiological relevance, can be used to analyze the vector's ecology and its association with transmission risk, and plan integrated regional vector surveillance and control programs. In this study, we model the environmental requirements of the principal North and Central American phlebotomine species and analyze three niche characteristics over future climate change scenarios: i) potential change in niche breadth, ii) direction and magnitude of niche centroid shifts, iii) shifts in elevation range. Niche identity between confirmed or incriminated *Leishmania* vector sand flies in Mexico, and human cases were analyzed. Niche models were constructed using sand fly occurrence datapoints from Canada, USA, Mexico, Guatemala and Belize. Nine non-correlated bioclimatic and four topographic data layers were used as niche components using GARP in OpenModeller. Both B2 and A2 climate change scenarios were used with two general circulation models for each scenario (CSIRO and HadCM3), for 2020, 2050 and 2080. There was an increase in niche breadth to 2080 in both scenarios for all species with the exception of *Lutzomyia vexator*. The principal direction of niche centroid displacement was to the northwest (64%), while the elevation range decreased greatest for tropical, and least for broad-range species. *Lutzomyia cruciata* is the only epidemiologically important species with high niche identity with that of *Leishmania* spp. in Mexico. Continued landscape modification in future climate change will provide an increased opportunity for the geographic expansion of NCA sand flys' ENM and human exposure to vectors of Leishmaniases.

## Author Summary

The present study models the niche of the most abundant sand fly species in North and Central America, including all proven and incriminated vectors of Leishmaniases, an important neglected tropical disease of the region. The expansion and elevation or centroid shifts of the species' niche are modeled for extreme (A2) and conservative (B2) climate change scenarios to 2020, 2050 and 2080. In climate change scenarios, models predict significant niche breadth changes in geographic space, principally in temperate sand fly species, while elevation shifts occur principally in tropical, and greatest, in vector species. Niche centroid shifts for individual species were predominately to the northwest, and secondarily to the northeast. The highest proportion of human population at-risk for contact with a vector species was with *Lutzomyia diabolica* and *Lutzomyia shannoni*. Despite the fact that *Lutzomyia olmeca olmeca* is the only confirmed vector species in Mexico, the present study demonstrates a significant niche identity between *Leishmania* spp. and *Lutzomyia cruciata*.

## Introduction

Leishmaniases are an increasingly important disease group worldwide, based on case numbers, geographic expansion, socioeconomic implications, psychological impact, and immunosuppression due to HIV infection which re-activates *Leishmania* spp. patency [1]. There are four main clinical manifestations of Leishmaniases: localized cutaneous leishmaniases (LCL), diffuse cutaneous leishmaniases, mucocutaneous leishmaniases and visceral leishmaniases (VL); the first (LCL) and last (VL) cause the greatest disease burden and mortality, respectively, for the disease group [2].

*Leishmania* spp. are transmitted by female sand flies of the genus *Lutzomyia* in the New World [2], [3]. In North and Central America (NCA) included in the North American tectonic plate which extends to southern Guatemala, 62 species of sand flies have been recorded [3]–[5], of which nine have been confirmed or incriminated as vectors of *Leishmania* [6]–[9]. The sand flies *Lutzomyia longipalpis* and *Lutzomyia evansi* are confirmed primary VL vectors in several countries [2], [10], whereas *Lutzomyia olmeca olmeca* is a confirmed vector of LCL in Mexico [6]. Other sand fly species, such as *Lutzomyia anthophora* [8], *Lutzomyia cruciata* [9], [11], *Lutzomyia diabolica* [7], *Lutzomyia ovallesi* [12], *Lutzomyia panamensis* [9], *Lutzomyia shannoni* [7], [9], and *Lutzomyia ylephiletor* [9], [13], however, have been found naturally infected or experimentally infected with *Leishmania* spp. [11]. Four species of *Leishmania* have been isolated in NCA, and are responsible for all human and canine clinical manifestations: *Leishmania mexicana*, *Leishmania braziliensis*, *Leishmania panamensis* and *Leishmania infantum chagasi* [2], [6], [14]. Clinical symptoms depend on the host species, its immune-competence, parasite species or strain, in addition to other as yet unidentified genetic determinants [15].

Despite early studies on the taxonomy and geographic distribution of sand flies in NCA, knowledge regarding the biology, distribution, and ecology of new collections and species continues to be registered from only a few regions [16]. Knowledge of current and potential sand fly distributions are important to predict the impact of environmental modification, the expansion of human settlements and migration, and climate change (CC) or its variation on parasite and vector population dynamics. Hence, there is a need for alternative tools to analyze species' distributions and potential sand fly dispersal areas [17], [18]. The choice and use of prevention strategies in risk areas for all Leishmaniases will depend on current and potential distributions of epidemiologically relevant species (ERS) [19].

Generally, macroclimatic variables influence species distributions at coarse scales, topographic variables at regional scales, and land use and biotic interactions at finer scales [20]. Whereas land use and biotic interactions are more related to demographic dynamics, macroclimatic variables determine the distributional limits while topography delineates physical barriers for dispersal [21], [22]. Therefore, species' geographic range shifts are predicted in the forthcoming decades, as a result of the accelerated rate of climate change [23] which reduces niche suitability in current locations, while offering new suitable colonization sites [24]. The change of at least two important attributes of a species' ecological niche, the niche breadth (the expressed geographic coverage of the abiotic niche related to the available geographic space) and the niche's geographic centroid (the geometric central point of the specie's geographic range which indicates the latitudinal mid-point of the range) would dramatically affect the geographic epidemiology of Leishmaniases in North America (i.e. the emergence of new regions where transmission cycles could be established due to the convergence of mammal hosts, parasite and vectors, and human population exposed to these vectors).

Ecological niche modeling (ENM) has already been used to project the geographic distribution potential of epidemiologically relevant Old World sand fly species: *Phlebotomus papatasi* [25], *Phlebotomus orientalis*, *Phlebotomus martini* [26], and *Phlebotomus alexandri* [25]. ENM have also been generated for a few New World, such as *Lutzomyia whitmani*, *Lutzomyia intermedia* and *Lutzomyia migonei* [17], and a few NCA species [16], [18]. Multiple abiotic and biotic factors have been associated with NCA sand fly species' distributions, in particular precipitation, temperature, altitude, latitude, physical barriers, and host distributions and abundance [27], [28]. While certain sand fly species exhibit local extinctions, others are predicted to adapt successfully and indeed to increase their relative abundance in modified habitats [29]. All of these factors also affect the spatial and temporal distribution of vectors and reservoirs, which in turn affect the epidemiology and dynamics of pathogen transmission to the human population [30]. Analysis of the impact of climate variability on Leishmaniases has focused principally on vector distributional changes due to El Niño [17], [18], [31], or using climate simulations [32].

All tropical and temperate NCA species from Guatemala and Belize to Canada are modeled together in this study, and niche characteristics as well as epidemiological associations of relevant species are analyzed in two contrasting CC scenarios. We have focused on analyzing potential change in species' geographic ranges as predicted by macroclimatic changes at the coarse-grain level, since these provide greater model consistency and accuracy for climate circulation models and their bioclimatic variables [33]. No reliable data layers for future land use changes are available to be incorporated into the niche models, although we use the differential between climate change scenarios to predict the impact of local scale habitat changes.

## Methods

### Study area

The study area for model construction and projection includes Canada, USA, Mexico, Guatemala and Belize, limited by 14.07°N, 58.23°N and −136.15°W, −56.29°W. The region was divided into 7,536,074 pixels at a resolution of 30 arc-seconds (0.008333°≈1 km) for latitude and longitude. Ecological region categories were assigned using the World Wildlife Fund (WWF) shape files based en Terrestrial Eco-regions of the World [34].

### Sand fly database

A database was constructed from collections reported in published scientific literature, entomological collections housed in several academic institutions in Mexico (Universidad Autónoma de Yucatán (UADY), El Colegio de la Frontera Sur (ECOSUR), and Universidad Autónoma de Nuevo León (UANL)), the Instituto Nacional de Diagnóstico y Referencia Epidemiológica (InDRE), and author's unpublished collections (Table S1). The database included 1,478 occurrence data points for 28 sand fly species with ≥10 records in the NCA region: Belize (N = 230), Canada (N = 2), USA (N = 208), Guatemala (N = 42) and Mexico (N = 996). In order to analyze niche shift trends, all species were assigned to one of three ecological region categories: tropical (moist and dry forest, n = 22; 1,306 data points), temperate (desert, grasslands, steppe, savanna, prairies, mountains forest, scrubland, pine forest, conifer forest, swamps, mangroves and mezquital, n = 4; 103 data points) and broad-range (species in both regions; n = 2; 69 data points) (Table 1).

**Table 1:** Eco-region category and data points for ENM maps of NCA sand fly species.

Eco-region	Species	Data points	ENM map figure
Tropical	*Brumptomyia hamata*	20	S1
	*Br. mesai*	68	S2
	*Lutzomyia beltrani*	17	S3
	*Lu. bispinosa*	12	S4
	*Lu. carpenteri*	46	S5
	*Lu. cayennensis*	55	S6
	*Lu. chiapanensis*	14	S7
	*Lu. cratifer*	25	S8
	*Lu. cruciata*	234	3
	*Lu. deleoni*	98	S9
	*Lu. dodgei*	11	S10
	*Lu. longipalpis*	43	S11
	*Lu. olmeca olmeca*	108	4
	*Lu. ovallesi*	55	S12
	*Lu. panamensis*	68	S13
	*Lu. permira*	23	S14
	*Lu. serrana*	17	S15
	*Lu. shannoni*	240	2
	*Lu. steatopyga*	43	S16
	*Lu. trinidadensis*	52	S17
	*Lu. undulata*	38	S18
	*Lu. ylephiletor*	17	S19
Temperate	*Lu. anthophora*	26	S20
	*Lu. californica*	17	S21
	*Lu. diabolica*	44	S22
	*Lu. stewarti*	16	S23
Broad-range	*Lu. texana*	29	S24
	*Lu. vexator*	42	5

**ENM maps for each species analyzed in both CSIRO and HadCM3 models and A2 and B2 climate change scenarios.**

### Ecological niche models (ENM)

Thirteen environmental layers were used for the construction of ENM. Nine bioclimatic data layers (annual mean temperature, temperature seasonality, maximum temperature of warmest month, minimum temperature of coldest month, temperature annual range, annual precipitation, precipitation of wettest month, precipitation of driest quarter and precipitation seasonality) were obtained from the Worldclim- Global Climate Data (www.worldclim.org; last accessed Nov, 2011) at a resolution of 30 arc-seconds [35]. These bioclimatic variables were selected from 19 by choosing the more meaningful variables hypothesized to limit species distribution at coarse-grain scale, after analysis of multicolinearity in a correlation matrix [18]. The final dataset layer includes variables with relatively low inter-correlation (r<0.75). Additionally, four topographic layers (aspect, slope, topographic index and elevation) obtained from the Hydro 1k data set (Earth Resources Observations and Science- http://eros.usgs.gov/products/elevation/gtopo30/gtopo30.html; last accessed Dec, 2011) were also used for ENM models.

ENM based on occurrence data, bioclimatic and topographic layers were constructed using the Genetic Algorithm for Rule-set Prediction (GARP) and *best subsets* implementation [36], [37] from the OpenModeller desktop *ver.* 1.1.0 [38]. In general, the procedure focuses on modeling the set of ecological conditions in which a species can maintain populations without immigration [39]. GARP is the preferred model for datasets which may have heterogeneous occurrence records across a broad geographic range. The software randomly divides occurrence points into training data for model building (75%) and test data for model testing (25%). One hundred replicate models were developed for each species and a soft omission threshold of 20% of the distribution was used for all [37].

Each ENM was evaluated using two tests: accuracy, a measure of performance, and the AUC (area under the receiver operating curve [ROC]), as a test of predictive ability. Both tests are based on two types of error: commission (areas of actual absence predicted present) and omission (areas of actual presence predicted absent) [37]. The internal (training data) and external (test data) accuracy was calculated using the confusion matrix, equivalent to “sensitivity” [a/(a+c)]. The AUC (ROC curve) was calculated using the values of “sensitivity” in the y-axis and the commission error in the x-axis, measuring the maximum inflection point where both errors are minimized. The AUC has a range of 0.0 to 1.0 (in general, acceptable models have AUC>0.85) [18]. We used a minimum presence threshold criterion of 90% in order to generate a binary map (presence/absence) of each projection from the 0–100 range of the model output. To do this, we first selected a set of 90% of random records per species and projected them onto the model. Then, we selected a threshold that predicted the presence of all of the 90% datapoints and converted the values≥of that number in “1” (presence) and the values<of that threshold in “0” (absence) to get a binary map of distribution. The binary maps were tested on training and test datasets, using a binomial test which evaluates the success rate of correct classification of presence data in comparison with random expectation [40].

Since there is no active epidemiological surveillance for Leishmaniases in Mexico, we use an identity test to identify niche overlap of *Leishmania* spp. (PEN) and each vector [41]. ENM were generated for all incriminated vector species: *Lu. anthophora*, *Lu. cruciata*, *Lu. diabolica*, *Lu. longipalpis*, *Lu. olmeca olmeca*, *Lu. ovallesi*, *Lu. panamensis*, *Lu. shannoni* and *Lu. ylephiletor*. Human cases of Leishmaniases from multiple Mexican states, Campeche (N = 8), Chiapas (N = 161), Guerrero (N = 10), Morelos (N = 2), Oaxaca (N = 3), Puebla (N = 4), Quintana Roo (N = 101), Tabasco (N = 15) and Veracruz (N = 37) were used as proxy to generate the PEN. A maximum-entropy-based algorithm, MaxEnt [42] was used to generate all vectors and PEN ENM using topographic and bioclimatic variables previously mentioned, since this spatially explicit test and corresponding statistical analyses are not available for GARP. The parameters to measure identity were the random test percentage (75%), replicated run type (bootstrap), maximum iterations (500), and the threshold rule (minimum training presence), using ENMtools *ver.*13.2 (http://enmtools.com/, last accessed Mar, 2012; [43]).

### Climate change models and scenarios

Two climate change scenarios were used: the A2 and B2 scenarios [44]. The A2 scenario assumes a rapid increase in human population, economy, technology, land use change, agriculture and energy consumption, while these parameters are more moderate in the B2 scenario. In the A2 scenario, there is an average of 3.4°C temperature increase for the year 2099, while in the B2 scenario, this increase would not supercede 2.4°C [33], [44].

Two general circulation models were used for both scenarios: CSIRO (CSIRO Division of Marine and Atmospheric Research, Australia [45]) and HadCM3 (Hadley Center for Climate Prediction and Research, England [45], [46]). Both models included four primary characteristics (atmosphere, ocean, sea ice and land) and feature a 1% increase to 2×CO_2_ at time of doubling. The CSIRO model uses an increase in 1.21°C, 2.05°C and 3.07°C for 2020, 2050, and 2080, respectively. The HadCM3 model uses an increase of 1.21°C, 2.10°C and 3.17°C for the same years [44]. Generally, the CSIRO model has better performance at a global level [45], while the HadCM3 model was chosen according to performance in reproducing regional climate for Mexico, Central America and the Caribbean [33].

### Data analyses

#### Characteristics of the effects of CC on sand fly ENM

Change in geographic niche breadth for each ENM was calculated using the proportion of occupied pixels/total number of pixels. The overlap for current, 2020, 2050, and the 2080 models was classified into three categories: 25–50%, 51–75% and 76–100%. A shift in the niche centroid was measured using the spatial analyst tool (Zonal>zonal geometry) of ESRI ArcMap 10.0 (www.esri.org; last accessed Sep, 2011). The centroid of each ENM (current+future) was connected between the previous and the azimuth of the trajectory measured for future scenarios. The elevation range change for each ENM was also measured with the spatial analyst tool (local>combine) from ArcMap.

#### Hazard of human exposure to sand flies in México

The total population growth rate for Mexico was generated using projections for fertility, mortality and international migration [47]. The population growth rate in Mexico is projected to increase by 30% for both 2030 and 2050. The current Mexican population from the 2010 census was 112,336,537 inhabitants (INEGI; www.censo2010.org.mx/; last accessed Feb, 2012). After obtaining the ENM, population projections were calculated using Hawth's Analysis Tool version 3.27 (Analysis tools<Intersect point tool) (www.spatialecology.com/htooldesc.php; last accessed Feb, 2012).

#### Niche identity

We calculated the “*Hellinger's-based I*” for all pairwise combinations of vector-*Leishmania* spp. The empirical measure of niche similarity between populations is compared to a null distribution to test whether they are significantly different from similarity generated from niche models constructed with data points extracted randomly from the distribution range of the compared species. The hypothesis of niche identity is rejected when the empirically observed value for “*Hellinger's based I*” is significantly lower than the values expected from the pseudoreplicate data sets [43], [48].

## Results

**Figure 1:**
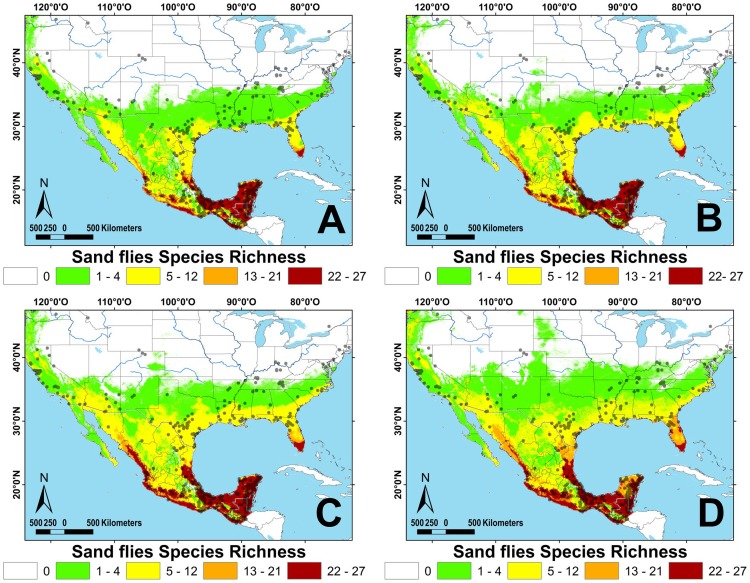
Sand fly richness for North and Central America, based on ecological niche models (ENM's). A: present, B: 2020, C: 2050, D: 2080. Dots represent sand fly collection sites.

**Figure 2:**
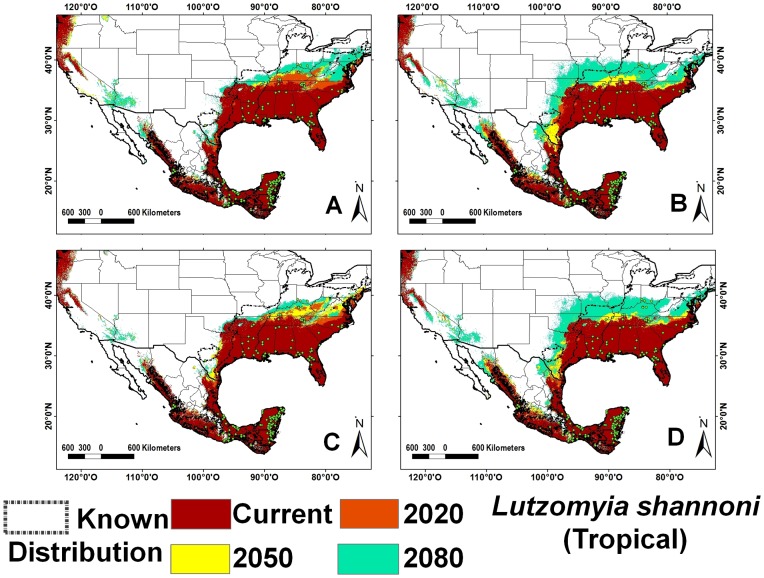
Ecological niche models for *Lutzomyia shannoni* (tropical). A) A2 scenario, CSIRO model; B) A2 scenario, HadCM3 model; C) B2 scenario, CSIRO model and D) B2 scenario, HadCM3 model.

**Figure 3:**
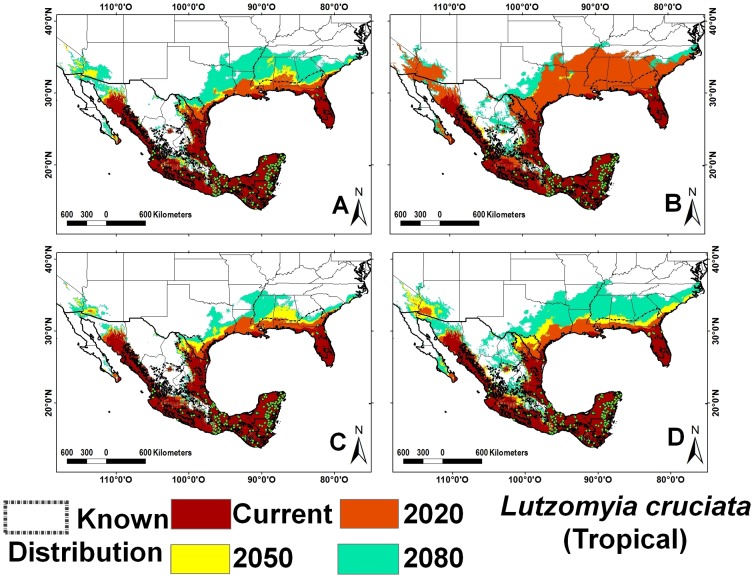
Ecological niche models for *Lutzomyia cruciata* (tropical). A) A2 scenario, CSIRO model; B) A2 scenario, HadCM3 model; C) B2 scenario, CSIRO model and D) B2 scenario, HadCM3 model.

**Figure 4:**
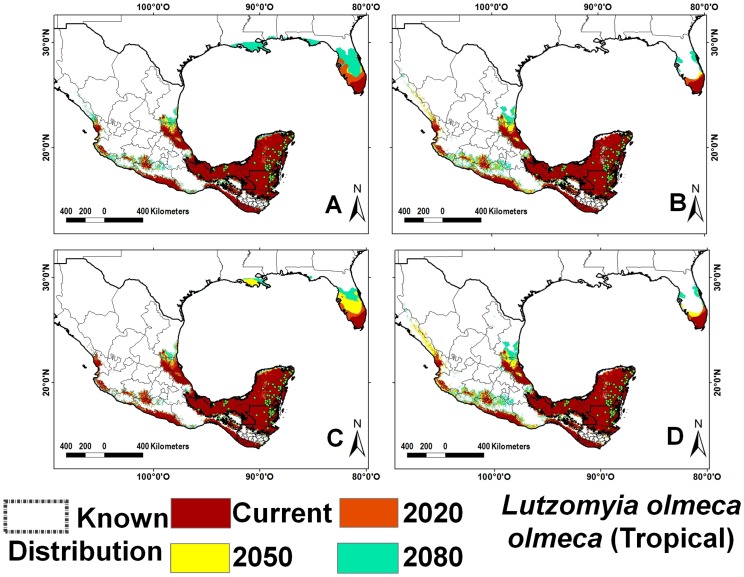
Ecological niche models for *Lutzomyia olmeca olmeca* (tropical). A) A2 scenario, CSIRO model; B) A2 scenario, HadCM3 model; C) B2 scenario, CSIRO model and D) B2 scenario, HadCM3 model.

Sand fly species richness is greater in tropical as compared with temperate climates. The tropical region includes southeastern Mexico, Guatemala, Belize, and Florida (USA), with the exception of the higher elevation areas of the Sierra Madre of Chiapas and the volcanic range in San Marcos, Guatemala (Figure 1A). The four species with greatest occurrence representing 46% of all records were *Lu. shannoni* (Figure 2), *Lu. cruciata* (Figure 3), *Lu. olmeca olmeca* (Figure 4), and *Lutzomyia deleoni* (Table 1).

**Figure 5:**
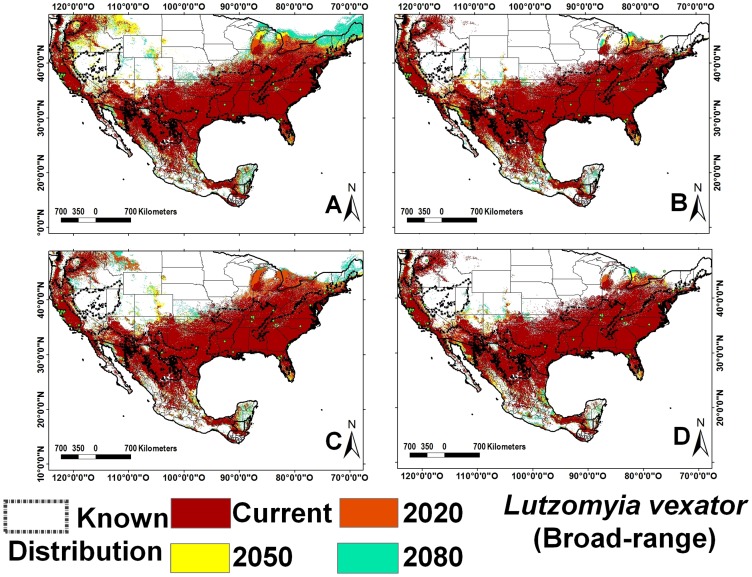
Ecological niche models for *Lutzomyia vexator* (broad-range). A) A2 scenario, CSIRO model; B) A2 scenario, HadCM3 model; C) B2 scenario, CSIRO model and D) B2 scenario, HadCM3 model.

All vector ENM models had high accuracy and high AUC values. The internal accuracy ranged from 0.83 to 1.00 (20 with an accuracy = 1.00) and external accuracy from 0.57 to 1.00 (20 with an accuracy = 1.00); the lowest accuracy values were calculated for *Lutzomyia vexator* (Figure 5) and *Lu. anthophora*. The AUC ranged from 0.80 to 0.99 (42 with an AUC higher than 0.96) for both training and test data. The *p*-values for all species were highly significant in predicting the known distribution of species (Table S2). Tropical species overlap approximately 3% of their ENM, while the *Lu. shannoni* ENM overlaps 86% with other species. The average overlap for temperate species is 9%, for broad-range species is 6%, and for ERS is 0.05%. Climate change models predict a broadening of regions with 5–11 species (140%) and 12–21 species (160%), northward, particularly along the Pacific coast of NA (Figure 1B, C, D). Highest species richness diminishes approximately 17% over time in the Yucatan Peninsula, especially along its western half, in the states of Yucatan and Campeche (Figure 1D, Table 2). Greatest species richness in Mexico increases over time in two particular regions: the Pacific coast north of the Tehuantepec isthmus, and the northern Gulf coast (Tamaulipas state).

**Table 2:** Changes in species richness over time in the B2 scenario using the HadCM3 model.

		2020		2050		2080	
Species richness	Current	km2	% change	km2	% change	km2	% change
1–4	4,313,812	3,741,763	−13.26	3,471,105	−19.54	3,591,081	−16.75
5–11	1,012,739	1,443,384	42.52	1,793,846	77.13	2,438,209	140.75
12–21	147,650	166,653	12.87	175,901	19.13	381,215	158.19
22–27	381,743	424,051	11.08	500,619	31.14	457,368	19.81

**Proportion change for 2020, 2050, and 2080 is expressed for each time period in comparison with the current value.**

### Niche breadth and its changes in CC scenarios

**Figure 6:**
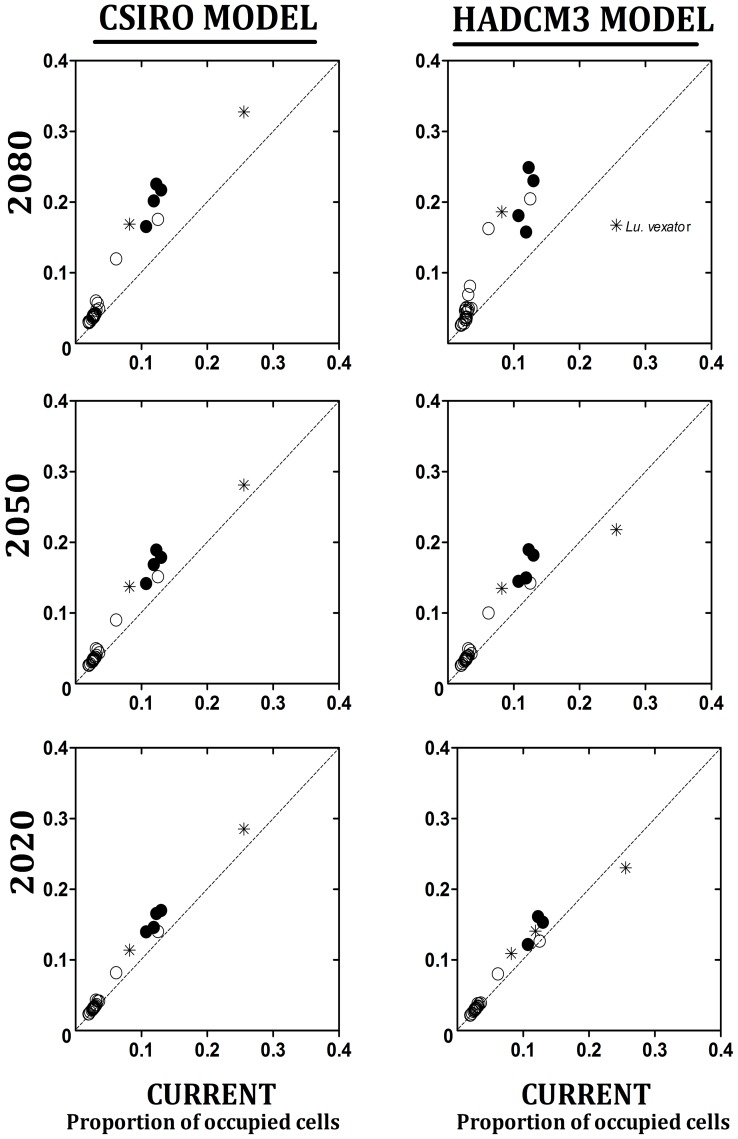
Proportion of pixels for sand fly ENM from current to 2080 in the B2 scenario. Predicted future scenarios using the CSIRO model (left) and HadCM3 model (right). Empty circles = tropical species; solid dot = temperate species; asterisk = broad-range species.

In both models and CC scenarios (B2 and A2), niche breadth increased over time for all but one species (Figure 6). In the CSIRO model *Lu. vexator's* ENM expanded over time as for all other species, while it contracted using the HadCM3 model. The geographic projection of this species' ENM diminished in the HadCM3 model, in northern regions, as well as fragments in the south. Average niche breadth increase was marginally greater in temperate and broad-range as compared with tropical species. Most tropical species had a greater increase in B2 than A2, while the opposite was observed for temperate species (Table S3). Species with the greatest ENM increase over time were *Lu. bispinosa*, *Lu. cruciata*, *Lu, ylephiletor*, *Lu. diabolica*, and *Lu. texana*, most of which had greatest change in the A2 scenario. Temperate as compared to tropical ERS species have greater breadth increase, specifically in the A2 scenario. Despite variable expansion of ENM in geographic space, the change in niche overlap between current and 2050 was minimal: 93.5% in A2 and 98.6% in B2 for tropical species, and 95.6% for A2 and 97.2% for B2 for temperate species (Table S2).

Changes in ENM overlap among sand fly species (11.41–80.20%) was variable according to distribution category and time period. Overlap between current and 2050 projections was lowest for temperate species (average 43.49%) and highest for tropical (67.44%) species (Table 3). The ERS have an intermediate average 55.3% overlap, as compared with 67.7% for non-incriminated vector species. In the A2 scenario, *Lu. cruciata* had the lowest overlap with other species (11.4%), while *Lutzomyia permira* (80.1%) and *Lutzomyia undulata* (80.2%) had the highest overlap with other species.

**Table 3:** Effect of climate change to 2050 on NCA sand fly ENM breadth, centroid and elevation shift.

Categories	Species	A2 SCENARIO			B2 SCENARIO		
		Range overlap conserved (%)	Centroid distance change (km)	Change in elevation range (km)	Range overlap conserved (%)	Centroid distance change (km)	Change in elevation range (km)
Tropical	*Brumptomyia hamata*	76.95	176.00	+105.00	77.31	175.00	−21.00
	*Br. mesai*	73.31	195.00	+60.00	75.52	199.00	−49.00
	*Lutzomyia beltrani*	67.72	278.00	−44.00	72.22	283.00	−138.00
	*Lu. bispinosa*	50.34	422.00	+18.00	55.89	399.00	−156.00
	*Lu. carpenteri*	67.42	181.00	+266.00	73.22	183.00	−69.00
	*Lu. cayennensis*	61.95	336.00	+5.00	74.12	232.00	−48.00
	*Lu. chiapanensis*	75.96	170.00	+4.00	76.66	242.00	−141.00
	*Lu. cratifer*	70.29	208.00	+78.00	73.91	255.00	−96.00
	*Lu. cruciata*	11.41	272.00	−126.00	41.21	220.00	−297.00
	*Lu. deleoni*	77.19	152.00	+67.00	77.48	187.00	−68.00
	*Lu. dodgei*	66.31	390.00	−28.00	75.31	263.00	−136.00
	*Lu. longipalpis*	61.29	421.00	−113.00	71.51	260.00	−177.00
	*Lu. olmeca olmeca*	59.43	341.00	+56.00	68.32	285.00	−72.00
	*Lu. ovallesi*	48.86	516.00	−27.00	62.39	371.00	−102.00
	*Lu. panamensis*	68.95	228.00	+148.00	74.00	235.00	+5.00
	*Lu. permira*	80.20	139.00	−21.00	80.10	183.00	−127.00
	*Lu. serrana*	77.00	162.00	+87.00	76.29	252.00	−100.00
	*Lu. shannoni*	23.40	119.00	−21.00	45.08	130.00	−117.00
	*Lu. steatopyga*	74.70	131.00	+5.00	73.78	235.00	−53.00
	*Lu. trinidadensis*	65.80	272.00	−77.00	74.94	204.00	−130.00
	*Lu. undulata*	78.40	144.00	+24.00	80.06	164.00	−20.00
	*Lu. ylephiletor*	64.90	318.00	−105.00	70.08	314.00	−107.00
Temperate	*Lu. anthophora*	51.47	238.00	+78.00	46.70	336.00	+68.00
	*Lu. californica*	26.14	257.00	−76.00	48.87	314.00	−200.00
	*Lu. diabolica*	68.50	262.00	+165.00	70.47	240.00	−7.00
	*Lu. stewarti*	25.30	220.00	−78.00	43.36	234.00	−303.00
Broad-range	*Lu. texana*	60.70	289.00	+127.00	62.94	282.00	+186.00
	*Lu. vexator*	46.10	116.00	−79.00	70.58	197.00	−115.00

**The HadCM3 model was used for B2 and A2 scenarios.**

### ENM centroid directional and amplitude shifts

There is a direction shift in ENM centroids for all species in all time periods and both CC scenarios; the majority of species shifted to the northwest (64.3%), followed by northeast (35.1%), and minimally to the southwest (0.6%). The direction shift was to the northeast for *Lu. longipalpis* and *Lu. panamensis*, and to the northwest for all other ERS. The distance shift of ENM centroids was variable (47–940 km) according to species and time periods (Table 3). As expected, the shift was greater in the A2 than in the B2 scenario. In general, centroid shifts were greatest for temperate species, followed by tropical and broad-range categories. *Lutzomyia ovallesi* (tropical) had the greatest centroid shift, followed by *Lutzomyia bispinosa* (tropical) and *Lu. anthophora* (temperate). In contrast, *Lu. shannoni* (tropical) has the lowest centroid shift of all 28 species.

### Elevation range changes in CC scenarios

Although the shift in elevation range is highly variable, the average range for all 28 species decreased in future CC scenarios. In general, the decrease in the A2 scenario was greater than in the B2 (Table 3). The elevation patterns of ERS did not change substantially, although tropical species such as *Lu. cruciata* and *Lu. longipalpis* shift to lower elevations. The elevation range of all broad-range species, as well as *Lu. anthophora* (temperate) and *Lutzomyia carpenteri* (tropical) increased.

### Effect of climate change on global ENM patterns

**Figure 7:**
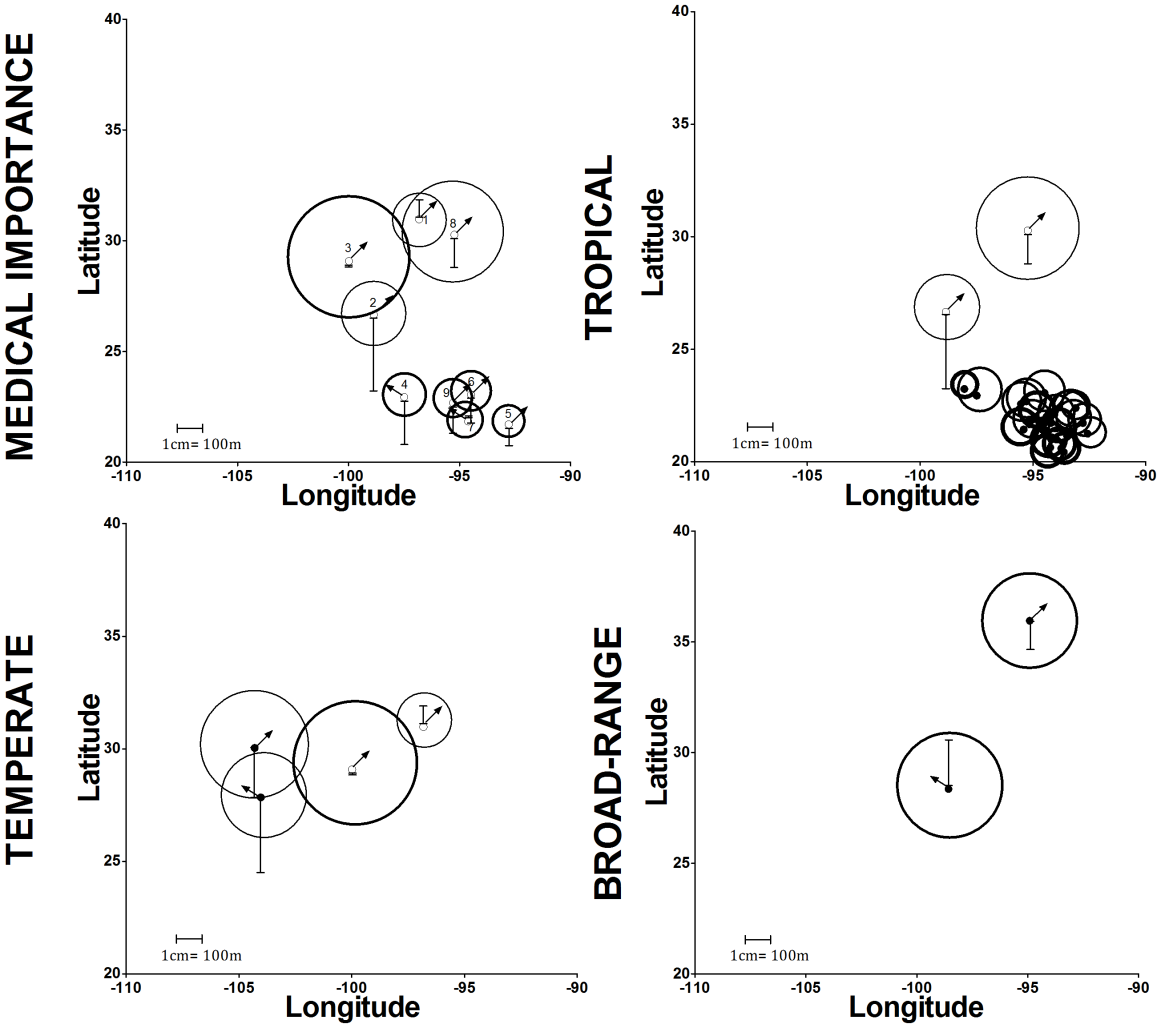
Changes in niche breadth, ENM centroid and elevational range in B2 scenario. The circle size represents the proportional distribution change, while the thickness is proportional to the overlap percentage between current and 2050 projections (border thickness of 0.5 pt = 25–50%; 1 pt = 51–75%; 2 pt = 76–100%). The direction of the arrow represents the direction of change of the centroid position and its size represents its magnitude. The elevational range changes are represented by the bars (bar up = increase; bar below = decrease). Epidemiologically important species are: 1) *Lu. anthophora*, 2) *Lu. cruciata*, 3) *Lu. diabolica*, 4) *Lu. longipalpis*, 5) *Lu. olmeca olmeca*, 6) *Lu. ovallesi*, 7) *Lu. panamensis*, 8) *Lu. shannoni* and 9) *Lu. ylephiletor*.

The combined changes in niche breadth, elevation and centroid range and direction was analyzed focusing on 2050; the CSIRO model was run using the B2 scenario (Figure 7), and the HadCM3 model with the A2 scenario (Figure S26). The pattern of niche breadth and centroid shift was similar between scenarios, although elevation range shifts were differentially affected in the combined analysis. The average maximum elevation was higher in the A2 as compared with the B2 scenario (1,147, 1,380 m, and 1,411 m for tropical, temperate and broad-range species, respectively).

Epidemiologically relevant sand flies had similar patterns in both scenarios. Based on these patterns in the B2 scenario, four categories of ERS were defined based on average elevation range shift: the first group includes only *Lu. olmeca olmeca* (average elevation range = 930 m), the second group includes *Lu. ovallesi*, *Lu. panamensis*, and *Lu. ylephiletor* (1,230 m), the third group contains only *Lu. longipalpis* (1,255 m), and the fourth group is composed of *Lu. anthophora*, *Lu. cruciata*, *Lu. diabolica*, and *Lu. shannoni* (1,599 m). In the A2 scenario, the average shift for the four groups was 1,058 m, 1,156 m, 1,319 m, and 1,590 m, respectively (Table 3).

### Epidemiological relevance of sand flies in Mexico

**Figure 8:**
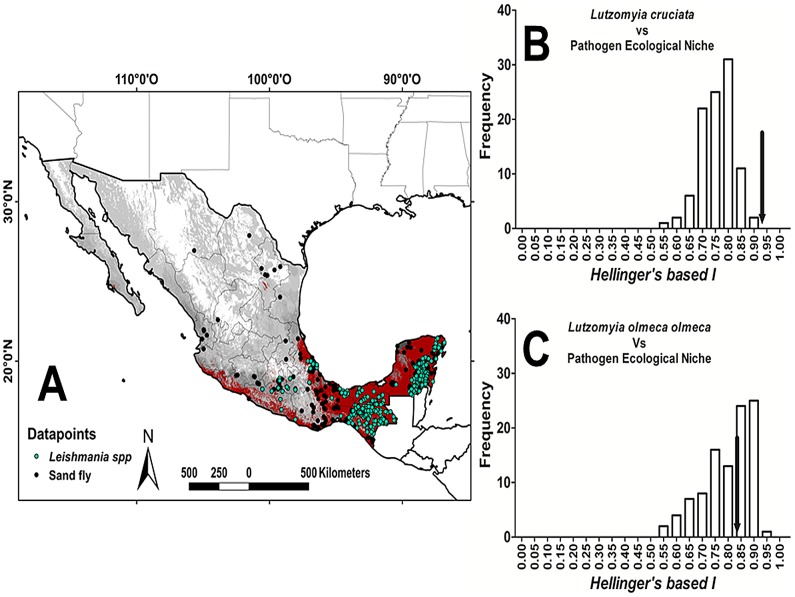
Niche identity test for sand fly species and *Leishmania* spp. (PEN), for Mexico. A) Map of sandfly species richness (compendium of all ENM models; gray), *Leishmania* spp. PEN (red), and datapoints for all sand flies and *Leishmania*; B) Niche identity test for *Lu. cruciata* and PEN; C) Niche identity test for *Lu. olmeca olmeca* and PEN. The histograms represents the entire range of *Hellinger's based I* values from the 100 random-drawn models, while the observed identity is represented by a black arrow.

The complete projected vector-exposed Mexican population, based on ENMs of ERS, was calculated separately for urban and rural communities. *Lutzomyia diabolica*'s niche covers the greatest total human population (107,176,279 inhabitants), followed by *Lu. shannoni* (71,002,449), *Lu. cruciata* (57,966,560), *Lu. longipalpis* (42,563,408), *Lu. ylephiletor* (32,403,860), *Lu. ovallesi* (30,792,955), *Lu. anthophora* (24,230,744), and *Lu. olmeca olmeca* (24,174,255). The rural population (communities <10,000 inhabitants) exposed to sand flies will increase over time in both CC scenarios (Table 4). *Lutzomyia diabolica*'s niche overlapped with the largest rural human population, while *Lu. anthophora*'s contained the least. Tropical sand flies *Lu. shannoni* and *Lu. cruciata* have ENM in areas with the highest proportion (20.7% and 21.0%, respectively) of exposed population, while *Lu. olmeca olmeca* overlaps with the lowest (8.9%). The only sand fly with significant niche identity with *Leishmania* spp. was *Lu. cruciata* (Figure 8 and Table 5).

**Table 4:** Rural population in Mexico with current hazard or that projected for 2050 for *Leishmania* spp. vector transmission.

Categories	Vector species	CURRENT	B2 SCENARIO 2050						A2 SCENARIO 2050					
			CSIRO			HadCM3			CSIRO			HadCM3		
			20	50	80	20	50	80	20	50	80	20	50	80
Tropical	*Lu. cruciata*	23.6	30.5	37.9	42.5	29.3	41.0	49.1	29.0	38.6	43.7	35.4	41.8	49.0
	*Lu. longipalpis*	16.6	21.5	26.5	28.7	20.4	26.9	32.8	21.1	26.8	31.1	20.4	27.1	32.8
	*Lu. o. olmeca*	10.0	13.1	16.1	17.6	12.2	17.1	18.8	12.5	16.2	18.2	12.3	16.6	18.8
	*Lu. ovallesi*	11.1	16.7	20.7	22.9	16.0	21.7	26.3	16.3	21.3	24.6	16.0	21.9	26.2
	*Lu. panamensis*	13.2	16.9	20.5	22.3	16.2	21.8	25.5	16.6	21.2	23.8	16.1	21.8	25.5
	*Lu. shannoni*	23.3	30.5	36.4	39.4	28.0	38.5	43.5	27.2	35.8	39.9	29.0	39.2	43.5
	*Lu. ylephiletor*	15.3	19.3	23.6	25.3	18.8	24.7	28.8	19.1	24.0	26.1	18.8	24.5	28.7
Temperate	*Lu. anthophora*	11.8	14.8	21.7	20.3	15.7	16.1	20.1	14.0	19.4	18.0	15.4	18.5	20.0
	*Lu. diabolica*	31.7	41.8	54.3	56.3	41.5	55.3	56.9	41.9	55.1	56.6	42.2	56.0	56.8

Census database from INEGI (www.inegi.mx); population expressed in millions.

**Table 5:** Ecological niche similarity between ENM of sand fly species and *Leishmania* spp.

Vector sand flies	Identity with PEN
*Lu. anthophora*	0.45^+^
*Lu. cruciata*	0.92*
*Lu. diabolica*	0.52^+^
*Lu. longipalpis*	0.83^+^
*Lu. o. olmeca*	0.82^+^
*Lu. ovallesi*	0.82^+^
*Lu. panamensis*	0.86^+^
*Lu. shannoni*	0.86^+^
*Lu. ylephiletor*	0.87^+^

Ecologically indistinct ENM have a significant value (p<0.05 = *), not significantly more distinct than expected at random (+), or when identity is not significant (p>0.05 = ns).

## Discussion

The present study models the niche and potential natural distribution of the most abundant 28 NCA sand fly species, and projects these in two CC scenarios using atmospheric, ocean surface, sea ice land surface and elevation characteristics, and climate models appropriate for the region [33]. Although there are approximately 500 phlebotomine sand fly species described in the Americas, NCA has the lowest sand fly diversity with only 62 species reported to date. We have focused this study on the 28 species which fulfilled minimum abundance collection registries for modeling confidence [3]. Even though the ecology of reservoir hosts and certain abiotic variables [16], [29] have been associated with sand fly distributions, their association with ecological niche modeled in a broad geographic area has not been analyzed. In addition, the differential impact of current versus accelerated environmental modifications (local anthropic change) in future CC scenarios has been explored for the geographic projection of the breadth, centroid location and elevation range shifts of these ENM.

Highest sand fly species diversity in NCA, based on ENM, occurs in the sub-tropical region of southern Mexico, Belize and Guatemala, including Florida. The present model confirms highest sand fly species' richness in areas where other taxa (mammal, reptile, bird, and amphibian) diversity is highest [49], [50], which may be due to the fact that major land-use change is far more advanced in temperate as compared to sub-tropical regions [51]. Greatest vector ENM shifts are projected to occur where historical environmental modifications have occurred in temperate areas (higher longitudes and lower elevations, [51]), and are projected to have greatest impact to 2020, 2050, and 2080, in the extreme A2 scenario in these same areas. More accelerated environmental modification coincides with broader human exposure to these vectors as observed both for overall sand fly distribution, and specifically for ERS, by comparing between CC scenarios [52]. It is interesting to note that the HadCM3 model, currently considered one of the more appropriate to model climate for Mexico [33], projects greater geographic expansion for all sand fly species, in comparison with CSIRO.

The geographic projection of niche breadth increase is uniform surrounding most NCA sand fly ENMs over time in both CC scenarios. This uniform increase depends on specific landscape components, biotic interactions, habitat modification, or other characteristics affecting population growth or the species' fitness, all of which affect the realized niche [32], [48], [53]. Temperate and broad-range sand fly species' niche are projected to increase more than that of tropical species, an expansion which may reflect an increase of generalist host species' resources in modified habitats. Jetz et al. [54] projected the impact of climate and land-use change on global bird diversity, and although greatest impact was expected in temperate areas, species at greatest risk are narrow-range species endemic to the tropics, where range reduction is a result of anthropogenic land conversion. In the present study, *Lu. shannoni* is projected to have the greatest increase in range size over time, perhaps due to the heterogeneous landscape where this species occurs: aquatic mangrove habitats, arid vegetation of spiny forest, desert, grasslands and xerophilous brushland, temperate vegetation of conifer and cloud forest and perennial, deciduous, and sub-deciduous tropical forest [54]. May et al. [55] have already reported a recent increase in *Lu. shannoni*'s relative abundance and habitat adaptation (conserved and modified habitats) at least in Quintana Roo, Mexico. Interestingly, *Lu. cruciata*, which also inhabits a wide variety of landscapes and is also a potential vector of *Leishmania* [55]–[57], has the second greatest projected increase in niche breadth due to CC in the present study.

The predominant trend for ENM centroid shift to the northwest was consistent over time for most sand fly species. However, the centroid of temperate species, which have the greatest ENM breadth increase, shifted predominately to the south, a trend previously observed in European birds [58]. Generally, the greater the expected environmental modification (A2), the greater was the distance shift in niche centroid, indicating that highly fragmented and degraded landscapes have a greater impact on sand fly ENM shifts. Loarie et al. [59] observed a trend for diversity shifts northward toward coastal areas in Californian flora; the species' centroids shifted by an average 151 km, to higher elevations. Huntley et al. [58] analyzing six climate scenarios to 2070-99 for 431 European bird species, observed a mean centroid shift of 258 to 882 km in a direction between 341° (NNW) and 45° (NE). Although these previous studies involve distant taxa, they represent evidence for species' ENM centroid shifts away from increasing climate values, positively associated with environmental modification.

In addition to potential increases in fundamental niche area and latitude/longitude centroid shifts, the elevation range for certain species have been reported, due to shifting or varying thermoclines and precipitation [57], [60]. The elevation range for all 28 sand fly species decreases over time and the greater the environmental modification (A2), the greater the decrease in ENM elevation range. *Lutzomyia intermedia* has an increased body size and greater dispersal capacity at higher elevations [61]. Previous studies of tropical sand fly species' ENM project an elevation range increase due to habitat modification in the southern tropical lowlands of NCA [51]. This trend is not homogeneous for ERS; some epidemiologically relevant species increase elevation range in CC scenarios (*Lu. anthophora*, *Lu. diabolica*, *Lu. olmeca olmeca* and *Lu. panamensis*), while others shift to a lower range (*Lu. cruciata*). Interestingly, an increase in Leishmaniases incidence in higher elevations has been reported in various countries, although it is not clear whether this is due to a prevalence shift of vector species, an increase in parasite prevalence only in certain vector species, or increased human exposure and changes in biotic interactions at higher elevations [62], [63]. Important changes in the geographic projection of ENM of temperate and broad-range sand fly species are also projected, although the dynamics and degree of these projected shifts are species-specific, with ERS having the least overall shift over time.

Niche breadth increase and centroid shifts of potential vector species could contribute to an increase in parasite dispersal and hence an increase in human transmission hazard. Even though the fundamental niche is projected to expand, dispersal capacity of the species will depend upon genetic plasticity, the availability of dispersal routes, and host interactions [59], [62]. The species with the least shift over time in distribution centroid was *Lu. shannoni*, which is the same species projected to have the greatest increase in total niche breadth. *Lutzomyia cruciata*, an ERS currently proposed to form a species complex [64], is the species with the second largest niche breadth increase and centroid shift.

The public health Secretariat in Mexico recognizes only 17,000 Leishmaniases cases over the last twenty-two years (CENAPRECE; www.dgepi.salud.gob.mx; last accessed Dec, 2012). However, there is no effective surveillance program and very poor knowledge by medical personnel of the diseases, and hence the total number of officially recognized cases may be much higher. Since rural populations are the principal group exposed to vector contact [65], there may be as many as 32 million inhabitants at-risk for exposure to transmission of Leishmaniases in Mexico. Population at-risk is projected to increase to 2080 [18], and based on significant niche identity, this increase corresponds principally to exposure from *Lu. diabolica*, *Lu. shannoni*, and *Lu. cruciata*.

Ecological niche modeling of pathogens has been applied to a broad range of infectious and toxicity-related diseases in order to project potential shifts to the end of the present century: dengue fever and *Aedes aegypti* [66], malaria transmission in Africa [67], plague and tularemia [68], and *Loxosceles reclusa* in the US [69]. Fundamental biodiversity analyses have used these same methods to model biotic community interactions and the impact of environmental modification, key issues affecting pathogen dispersal: Argentinian ants [70], Canadian butterfly species [71], European birds [58], amphibians in Australia [72], mammals in Spain [73], and maize races in Mexico [74]. In the present analysis, only *Lu. cruciata* has significant niche identity with that of human infection with *Leishmania* spp.. González et al. [16] reported an association between recurrent Leishmaniases transmission areas and *Lu. panamensis* (91.08%) and *Lu. olmeca olmeca* (84.84%), based only on geographic overlap. Additional studies will be required to analyze landscape quality and its impact on niche overlap areas where vector, reservoir, and parasite species interact, in order to extend the use of niche identity analysis within fragmented landscapes.

The present analysis of the distribution of NCA sand fly species and their ENM shifts in climate change scenarios predicts range shifts which may modify vector-host interactions and relationships associated with habitat and future land use. Temperate sand fly species, and therefore those with least epidemiological importance, project the greatest ENM changes. Those changes projected for certain epidemiologically relevant tropical species support previous evidence for current, and also highlight future importance of *Lu. cruciata* as an important vector of *Leishmania* spp. in México.

## Supporting Information

Figure S1 **Ecological niche models for** ***Brumptomyia hamata*** **(tropical).** A) A2 scenario, CSIRO model; B) A2 scenario, HadCM3 model; C) B2 scenario, CSIRO model and D) B2 scenario, HadCM3 model.
(TIF)

Figure S2 **Ecological niche models for** ***Brumptomyia mesai*** **(tropical).** A) A2 scenario, CSIRO model; B) A2 scenario, HadCM3 model; C) B2 scenario, CSIRO model and D) B2 scenario, HadCM3 model.
(TIF)

Figure S3 **Ecological niche models for** ***Lutzomyia beltrani*** **(tropical).** A) A2 scenario, CSIRO model; B) A2 scenario, HadCM3 model; C) B2 scenario, CSIRO model and D) B2 scenario, HadCM3 model.
(TIF)

Figure S4 **Ecological niche models for** ***Lutzomyia bispinosa*** **(tropical).** A) A2 scenario, CSIRO model; B) A2 scenario, HadCM3 model; C) B2 scenario, CSIRO model and D) B2 scenario, HadCM3 model.
(TIF)

Figure S5 **Ecological niche models for** ***Lutzomyia carpenteri*** **(tropical).** A) A2 scenario, CSIRO model; B) A2 scenario, HadCM3 model; C) B2 scenario, CSIRO model and D) B2 scenario, HadCM3 model.
(TIF)

Figure S6 **Ecological niche models for** ***Lutzomyia cayennensis*** **(tropical).** A) A2 scenario, CSIRO model; B) A2 scenario, HadCM3 model; C) B2 scenario, CSIRO model and D) B2 scenario, HadCM3 model.
(TIF)

Figure S7 **Ecological niche models for** ***Lutzomyia chiapanensis*** **(tropical).** A) A2 scenario, CSIRO model; B) A2 scenario, HadCM3 model; C) B2 scenario, CSIRO model and D) B2 scenario, HadCM3 model.
(TIF)

Figure S8 **Ecological niche models for** ***Lutzomyia cratifer*** **(tropical).** A) A2 scenario, CSIRO model; B) A2 scenario, HadCM3 model; C) B2 scenario, CSIRO model and D) B2 scenario, HadCM3 model.
(TIF)

Figure S9 **Ecological niche models for** ***Lutzomyia deleoni*** **(tropical).** A) A2 scenario, CSIRO model; B) A2 scenario, HadCM3 model; C) B2 scenario, CSIRO model and D) B2 scenario, HadCM3 model.
(TIF)

Figure S10 **Ecological niche models for** ***Lutzomyia dodgei*** **(tropical).** A) A2 scenario, CSIRO model; B) A2 scenario, HadCM3 model; C) B2 scenario, CSIRO model and D) B2 scenario, HadCM3 model.
(TIF)

Figure S11 **Ecological niche models for** ***Lutzomyia longipalpis*** **(tropical).** A) A2 scenario, CSIRO model; B) A2 scenario, HadCM3 model; C) B2 scenario, CSIRO model and D) B2 scenario, HadCM3 model.
(TIF)

Figure S12 **Ecological niche models for** ***Lutzomyia ovallesi*** **(tropical).** A) A2 scenario, CSIRO model; B) A2 scenario, HadCM3 model; C) B2 scenario, CSIRO model and D) B2 scenario, HadCM3 model.
(TIF)

Figure S13 **Ecological niche models for** ***Lutzomyia panamensis*** **(tropical).** A) A2 scenario, CSIRO model; B) A2 scenario, HadCM3 model; C) B2 scenario, CSIRO model and D) B2 scenario, HadCM3 model.
(TIF)

Figure S14 **Ecological niche models for** ***Lutzomyia permira*** **(tropical).** A) A2 scenario, CSIRO model; B) A2 scenario, HadCM3 model; C) B2 scenario, CSIRO model and D) B2 scenario, HadCM3 model.
(TIF)

Figure S15 **Ecological niche models for** ***Lutzomyia serrana*** **(tropical).** A) A2 scenario, CSIRO model; B) A2 scenario, HadCM3 model; C) B2 scenario, CSIRO model and D) B2 scenario, HadCM3 model.
(TIF)

Figure S16 **Ecological niche models for** ***Lutzomyia steatopyga*** **(tropical).** A) A2 scenario, CSIRO model; B) A2 scenario, HadCM3 model; C) B2 scenario, CSIRO model and D) B2 scenario, HadCM3 model.
(TIF)

Figure S17 **Ecological niche models for** ***Lutzomyia trinidadensis*** **(tropical).** A) A2 scenario, CSIRO model; B) A2 scenario, HadCM3 model; C) B2 scenario, CSIRO model and D) B2 scenario, HadCM3 model.
(TIF)

Figure S18 **Ecological niche models for** ***Lutzomyia undulata*** **(tropical).** A) A2 scenario, CSIRO model; B) A2 scenario, HadCM3 model; C) B2 scenario, CSIRO model and D) B2 scenario, HadCM3 model.
(TIF)

Figure S19 **Ecological niche models for** ***Lutzomyia ylephiletor*** **(tropical).** A) A2 scenario, CSIRO model; B) A2 scenario, HadCM3 model; C) B2 scenario, CSIRO model and D) B2 scenario, HadCM3 model.
(TIF)

Figure S20 **Ecological niche models for** ***Lutzomyia anthophora*** **(temperate).** A) A2 scenario, CSIRO model; B) A2 scenario, HadCM3 model; C) B2 scenario, CSIRO model and D) B2 scenario, HadCM3 model.
(TIF)

Figure S21 **Ecological niche models for** ***Lutzomyia californica*** **(temperate).** A) A2 scenario, CSIRO model; B) A2 scenario, HadCM3 model; C) B2 scenario, CSIRO model and D) B2 scenario, HadCM3 model.
(TIF)

Figure S22 **Ecological niche models for** ***Lutzomyia diabolica*** **(temperate).** A) A2 scenario, CSIRO model; B) A2 scenario, HadCM3 model; C) B2 scenario, CSIRO model and D) B2 scenario, HadCM3 model.
(TIF)

Figure S23 **Ecological niche models for** ***Lutzomyia stewarti*** **(temperate).** A) A2 scenario, CSIRO model; B) A2 scenario, HadCM3 model; C) B2 scenario, CSIRO model and D) B2 scenario, HadCM3 model.
(TIF)

Figure S24 **Ecological niche models for** ***Lutzomyia texana*** **(broad-range).** A) A2 scenario, CSIRO model; B) A2 scenario, HadCM3 model; C) B2 scenario, CSIRO model and D) B2 scenario, HadCM3 model.
(TIF)

Figure S25 **Proportion of pixels for sand fly ENM from current to 2080 in the A2 scenario.** Predicted future scenarios using the CSIRO model (left) and HadCM3 model (right). Empty circles = tropical species; solid dot = temperate species; asterisk = broad-range species.
(TIF)

Figure S26 **Changes in niche breadth, ENM centroid and elevational range in A2 scenario.** The circle size represents the proportional distribution change, while the thickness is proportional to the overlap percentage between current and 2050 projections (border thickness of 0.5 pt = 25–50%; 1 pt = 51–75%; 2 pt = 76–100%). The direction of the arrow represents the direction of change of the centroid position and its size represents its magnitude. The elevational range changes are represented by the bars (bar up = increase; bar below = decrease). Epidemiologically important species are: 1) *Lu. anthophora*, 2) *Lu. cruciata*, 3) *Lu. diabolica*, 4) *Lu. longipalpis*, 5) *Lu. olmeca olmeca*, 6) *Lu. ovallesi*, 7) *Lu. panamensis*, 8) *Lu. shannoni and* 9) *Lu. ylephiletor*.
(TIF)

Table S1 **Reference points for sand fly collection records used in ENM modeling of NCA species.**
(PDF)

Table S2 **Test accuracy, AUC (ROC curve) and statistical significance.**
(PDF)

Table S3 **Proportion of sand fly species' ENM overlap and their territorial projection of change to 2050.**
(PDF)

## References

[1] World Health Organization (2010) Report of a meeting of the WHO Expert Committee on the Control of Leishmaniases, Geneva, Switzerland 201.

[2] Killick-Kendrick R (1999) The biology and control of Phlebotominae sand flies. Clin Dermatol 17: 279–289. 10384867 10.1016/s0738-081x(99)00046-2

[3] Young DG, Duncan MA (1994) Guide to the identification and geographic distribution of *Lutzomyia* sand flies in México, the West Indies, Central and South American (Diptera: Psychodidae). Mem Amer Ent Inst. Gainesville: Associated Publishers. 881 pp.

[4] Ibáñez-Bernal S (2001) Notes on the Psychodidae (Diptera) of Belize: Subfamilies Bruchomyiinae and Phlebotominae. Ann Entomol Soc Am 94: 367–385.

[5] Ibáñez-Bernal S, May-Uc E, Rebollar-Téllez EA (2010) Two new species of phlebotomine sand flies (Diptera: Psychodidae, Phlebotominae) from Quintana Roo, Mexico. Zootaxa 2448: 26–34.

[6] Biagi F, de Biagi A, Beltrán F (1965) *Phlebotomus flaviscutellatus*, transmisor natural de *Leishmania mexicana* . Prensa Med Mex 30: 267–272. 5598515

[7] Lawyer P, Young D, Butler J, Akin D (1987) Development of *Leishmania mexicana* in *Lutzomyia diabolica* and *Lutzomyia shannoni* (Diptera: Psychodidae). J Med Entomol 24: 347–355. 3585930 10.1093/jmedent/24.3.347

[8] McHugh C, Grogl M, Kreutzer R (1993) Isolation of *Leishmania mexicana* (Kinetoplastida: Trypanosomatidae) from *Lutzomyia anthophora* (Diptera: Psychodidae) collected in Texas. J Med Entomol 30: 631–633. 8510126 10.1093/jmedent/30.3.631

[9] Pech-May A, Escobedo F, Berzunza-Cruz M, Rebollar-Téllez EA (2010) Incrimination of four sand fly previously unrecognized as vectors of *Leishmania* parasites in Mexico. Med Vet Entomol 24: 150–161. 20604861 10.1111/j.1365-2915.2010.00870.x

[10] Travi B, Vélez D, Brutus L, Segura I, Jaramillo C, et al (1990) *Lutzomyia evansi*, an alternate vector of *Leishmania chagasi* in a Colombian focus of visceral leishmaniasis. Trans R Soc Trop Med Hyg 84: 676–677. 2278068 10.1016/0035-9203(90)90142-2

[11] Williams P (1966) Experimental transmission of *Leishmania mexicana* by *Lutzomyia cruciata* . Ann Trop Med Parasit 60: 365–370. 5971136

[12] Rowton E, de Mata M, Rizzo N, Porter C, Navin R (1992) Isolation of *Leishmania braziliensis* from *Lutzomyia ovallesi* (Diptera: Psychodidae) in Guatemala. Am J Trop Med Hyg 46: 465–468. 1575293 10.4269/ajtmh.1992.46.465

[13] Porter C, Steurer FJ, Kreutzer RD (1987) Isolation of *Leishmania mexicana mexicana* from *Lutzomyia ylephiletor* in Guatemala. Trans R Soc Trop Med Hyg 81: 929–930. 3503412 10.1016/0035-9203(87)90355-5

[14] Lainson R (2010) The neotropical *Leishmania* species: a brief historical review of the discovery, ecology and taxonomy. Rev Pan-Amaz Saude 1: 13–32.

[15] Silveira FT, Lainson R, Corbett C (2004) Clinical and inmunopathological spectrum of American cutaneous Leishmaniasis with special reference to the disease in Amazonia Brazil- a review. Mem Inst Oswaldo Cruz 99: 239–251. 15273794 10.1590/s0074-02762004000300001

[16] González C, Rebollar-Téllez EA, Ibáñez-Bernal S, Becker-Fauser I, Martínez-Meyer E, et al (2011) Current knowledge of *Leishmania* vector in México: how species' geographic distributions relate to transmission areas. Am J Trop Med Hyg 85: 839–846. 22049037 10.4269/ajtmh.2011.10-0452 PMC3205629

[17] Peterson AT, Shaw J (2003) *Lutzomyia* vector for cutaneous leishmaniasis in Southern Brazil: ecological niche models, predicted geographic distributions, and climate change effects. Int J Parasitol 33: 919–931. 12906876 10.1016/s0020-7519(03)00094-8

[18] González C, Wang O, Strutz S, González-Salazar C, Sánchez-Cordero V, et al (2010) Climate change and risk of Leishmaniasis in North America: Predictions from ecological niche models of vector and reservoirs species. PLoS Negl Trop Dis 4: e585 doi:10.1371/journal.pntd.0000585 20098495 10.1371/journal.pntd.0000585 PMC2799657

[19] Hartemink N, Vanwambeke SO, Heesterbeek H, Roger D, Morley D, et al (2011) Integrated mapping of establishment risk for emerging vector-borne infections: A case study of canine Leishmaniasis in Southwest France. PLos ONE 6: e20817 doi:10.1371/journal.pone.0020817 21857899 10.1371/journal.pone.0020817 PMC3153454

[20] Peterson AT, Soberón J, Pearson RG, Anderson RP, Martínez-Meyer E, et al.. (2011) Ecological niches and geographic distributions. Princeton University Press. 328 p.

[21] Barve N, Barve V, Jiménez-Valverde A, Lira-Noriega A, Maher S, et al (2011) The crucial role of the accessible area in ecological niche modeling and species distribution modeling. Ecol Mod 222: 1810–1819.

[22] Soberón J (2007) Grinnellian and Eltonian niches and geographic distributions of species. Ecol Lett 10: 1115–1123. 17850335 10.1111/j.1461-0248.2007.01107.x

[23] Thuiller W (2007) Biodiversity: Climate change and the ecologist. Nature 448: 550–552. 17671497 10.1038/448550a

[24] Aitken SN, Yeaman S, Holliday J, Wang T, Curtis-McLane S (2008) Adaptation, migration or extirpation: climate change outcomes for tree populations. Evol Appl 1: 95–111. 25567494 10.1111/j.1752-4571.2007.00013.x PMC3352395

[25] Colacicco-Mayhugh M, Masuoka P, Grieco J (2010) Ecological niche model of *Phlebotomus alexandri* and *P. papatasi* (Diptera: Psychodidae) in the Middle East. Int J Health Geogr 9: 1–9. 20089198 10.1186/1476-072X-9-2 PMC2823717

[26] Gebre-Michael T, Malone J, Balkew M, Ali A, Berhe N, et al (2004) Mapping the potential distribution of *Phlebotomus martini* and *P. orientalis* (Diptera: Psychodidae), vectors of Kala-azar in East Africa by use of geographic information systems. Acta Trop 90: 73–86. 14739026 10.1016/j.actatropica.2003.09.021

[27] Stephens CR, Heau JG, González C, Ibarra-Cerdeña CN, Sánchez-Cordero V, et al (2009) Using biotic interaction networks for prediction in Biodiversity and Emerging Diseases. PLoS ONE 4: e5725 doi: 10.1371/journal.pone.0005725 19478956 10.1371/journal.pone.0005725 PMC2685974

[28] González-Salazar C, Stephens CR (2012) Constructing ecological networks: A tools to infer risk of transmission and dispersal of Leishmaniasis. Zoonoses Public Hlth 59: 179–193. 10.1111/j.1863-2378.2012.01479.x 22958263

[29] Bejarano E, Uribe S, Rojas W, Vélez I (2002) Phlebotomine sand flies (Diptera: Psychodidae) associated with the appearance of urban Leishmaniasis in the City of Sincelejo, Colombia. Mem Inst Oswaldo Cruz 97: 645–647. 12219128

[30] Rohr J, Dobson A, Johnson P, Marm A, Paull S, et al (2011) Frontier in climate change-disease research. Trends Ecol Evol 26: 270–277. 21481487 10.1016/j.tree.2011.03.002 PMC3374867

[31] Cárdenas R, Sandoval C, Rodríguez A, Franco C (2006) Impact of climate variability in the occurrence of Leishmaniasis in northeastern Colombia. Am J Trop Med Hyg 75: 273–277. 16896132

[32] Chaves L, Cohen J, Pascual M, Wilson M (2008) Social exclusion modifies climate and deforestation impacts on a vector-borne disease. PLoS Negl Trop Dis 2: e176 doi:10.1371/journal.pntd.0000176 18265876 10.1371/journal.pntd.0000176 PMC2238711

[33] Conde C, Estrada F, Martínez B, Sánchez O, Gay C (2011) Regional climate change scenarios for Mexico. Atmósfera 24: 125–140.

[34] Olson DM, Dinerstein E, Wikramanayake DE, Burgess ND, Powell GVN, et al (2001) Terrestrial ecoregions of the world: a new map of life on earth. BioScience 51: 933–938.

[35] Hijmans RJ, Cameron SE, Parra JL, Jones PG, Jarvis A (2005) Very high resolution interpolated climate surfaces for global land areas. Int J Climatol 25: 1965–1978.

[36] Stockwell D, Peters D (1999) The GARP modelling systems: Problems and solutions to automated spatial prediction. Int J Geogr Inf Syst 13: 143–158.

[37] Anderson R, Lew D, Peterson AT (2003) Evaluating predictive models of specie's distributions: Criteria for selecting optimal models. Ecol Mod 162: 211–232.

[38] de Souza M, de Giovanni R, Ferreira M, Sutton T, Brewer P, et al (2011) openModeller: a generic approach to specie's potential distribution modelling. Geoinformatica 15: 111–135.

[39] Soberón J, Peterson AT (2005) Interpretation of models of fundamental ecological niches and species' distributional areas. Biodivers Inform 2: 1–10.

[40] Raxworthy C, Martinez-Meyer E, Horning N, Nussbaum R, Schneider G, et al (2003) Predicting distributions of known and unknown reptile species in Madagascar. Nature 426: 837–841. 14685238 10.1038/nature02205

[41] Maher S, Ellis C, Gage K, Enscore R, Peterson AT (2010) Range-wide determinants of plague distribution in North America. Am J Trop Med Hyg 83: 736–742. 20889857 10.4269/ajtmh.2010.10-0042 PMC2946734

[42] Phillips S, Anderson R, Schapire R (2006) Maximum entropy modeling of species geographic distributions. Ecol Mod 190: 231–259.

[43] Warren D, Glor R, Turelli M (2010) ENMtools: a toolbox for comparative studies of environmental niche models. Ecography 33: 607–611.

[44] Intergovernmental Panel on Climate Change (2007) Cambio climático 2007. Informe de Síntesis. Contribución de los grupos de trabajo I, II y III al Cuarto Informe de evaluación del Grupo Intergubernamental de Expertos sobre el Cambio climático. Geneva, Switzerland. 104 pp.

[45] Gordon HB, Rotstayn LD, McGregor JL, Dix MR, Kowalczyk EA, et al.. (2002) The CSIRO Mk3 Climate System Model [Electronic publication]. Aspendale: CSIRO Atmospheric Research. (CSIRO Atmospheric Research technical paper; no. 60). 130 pp.

[46] Johns TC, Gregory JM, Ingram WJ, Johnson CE, Jones A, et al (2003) Anthropogenic climate change for 1860 to 2100 simulated with the HadCM3 model under updated emissions scenarios. Clim Dyn 20: 583–612.

[47] Consejo Nacional de Población (2005) Proyecciones de población de México 2005–2050. México, D.F. 29 pp.

[48] Warren D, Glor R, Turelli M (2008) Environmental niche equivalency versus conservatism: quantitative approaches to niche evolution. Evolution 62: 2868–2883. 18752605 10.1111/j.1558-5646.2008.00482.x

[49] Rodríguez P, Soberón J, Arita H (2003) El componente Beta de la diversidad de mamíferos de México. Acta Zool Mex 89: 241–259.

[50] Trejo I, Martínez-Meyer E, Calixto-Pérez E, Sánchez-Colon S, Vázquez de la Torre R, et al (2011) Analysis of the effects of climate change on plant communities and mammals in Mexico. Atmósfera 24: 1–14.

[51] Sala O, Stuart F, Armesto JJ, Berlow E, Bloomfield J, et al (2000) Global Biodiversity Scenarios for the year 2100. Science 287: 1770–1774 DOI:10.1126/science.287.5459.1770 10710299 10.1126/science.287.5459.1770

[52] Rousteenoja K, Carter T, Jylha K, Toumenvirta H (2003) Future climate in World regions: An intercomparison of model-based projections for the New IPCC Emission Scenarios. Finnish Environment Institute, Kelsinki. 83pp.

[53] Pearman P, Guisan A, Broenninmann O, Randin C (2007) Niche dynamics in space and time. Trends Ecol Evol 23: 149–158. 10.1016/j.tree.2007.11.005 18289716

[54] Jetz W, Wilcove D, Dobson A (2007) Projected impacts of climate change and land-use change on the global diversity of birds. PLoS Biol 5: e157 doi:10.1371/journal.pbio.0050157 17550306 10.1371/journal.pbio.0050157 PMC1885834

[55] May E, Hernández H, Rebollar-Téllez EA (2011) Distribución de flebotomineos (Diptera: Psychodidae) en Quintana Roo, México. Acta Zool Mex 27: 273–289.

[56] Rebollar-Téllez EA, Ramírez A, Andrade F (1996) A two years study on vectors of cutaneous Leishmaniasis. Evidence for sylvatic transmission cycle in the state of Campeche, Mexico. Mem Inst Oswaldo Cruz 91: 555–560. 9137741 10.1590/s0074-02761996000500004

[57] Rebollar-Téllez EA, Manrique-Saide P (2001) New distributional record of *Lutzomyia cruciata* (Diptera: Psychodidae) in the state of Yucatan, Mexico. Entomol News 112: 337–339.

[58] Huntley B, Collingham Y, Willis S, Green R (2008) Potential impacts of climate change on European breeding birds. PLoS One 3: e1439 doi:10.1371/journal.pone.0001439 18197250 10.1371/journal.pone.0001439 PMC2186378

[59] Loarie S, Carter B, Hayhoe K, McMahon S, Moe R, et al (2008) Climate change and the future of California's endemic flora. PLoS One 3: e2502. doi: 10.1371/journal.pone.0002502 18648541 10.1371/journal.pone.0002502 PMC2481286

[60] Barón S, Morilla F, Morales M, Díaz V, Irigaray C, et al (2011) Risk maps for the presence and absence of *Phlebotomus perniciosus* in an endemic area of Leishmaniasis in southern Spain: implications for the control of the disease. Parasitol 138: 1234–1244. 10.1017/S0031182011000953 21854702

[61] Brisola C, Leuch A, Falqueto A, Brazil R, Galati E, et al (1999) Influence of Altitude, Latitude and season of collection (Bergmann's Rule) on the dimensions of *Lutzomyia intermedia* (Lutz & Neiva, 1912) (Diptera, Psychodidae, Phlebtominae). Mem Inst Oswaldo Cruz 94: 693–700. 10464420 10.1590/s0074-02761999000500026

[62] Elnaiem DE, Schorscher J, Bendall A, Obsomer V, Osman M, et al (2003) Risk mapping of visceral Leishmaniasis: The role of local variation in rainfall and altitude on the presence and incidence of Kala-azar in eastern Sudan. Am J Trop Med Hyg 68: 10–17. 12556141

[63] Guernaoui S, Boumezzough A, Laamarani A (2006) Altitudinal structuring of sand flies (Diptera: Psychodidae) in the High-Atlas mountains (Morocco) and its relation to the risk of leishmaniasis transmission. Acta Trop 97: 346–351. 16460654 10.1016/j.actatropica.2006.01.001

[64] Pech-May A, Marina CF, Vázquez-Domínguez E, Berzunza-Cruz M, Rebollar-Téllez EA, et al (2013) Genetic structure and divergence in populations of Lutzomyia cruciata, a phlebotomine sand fly (Diptera: Psychodidae) vector of Leishmania Mexicana in southeastern Mexico. Infect Gen Evol 16: 254–262. 10.1016/j.meegid.2013.02.004 23416432

[65] Sánchez-Tejada G, Rodríguez N, Parra C, Hernández O, Barker D, et al (2001) Cutaneous leishmaniasis caused by members of *Leishmania braziliensis* complex in Nayarit, State of Mexico. Mem Inst Oswaldo Cruz 96: 15–19. 11285471 10.1590/s0074-02762001000100002

[66] Hales S, de Wet N, Maindonald J, Woodward A (2002) Potential effect of population and climate change on global distribution of Dengue fever: an empirical model. Lancet 360: 830–834. 12243917 10.1016/S0140-6736(02)09964-6

[67] Tanser F, Sharp B, le Sueur D (2003) Potential effect of climate change on malaria transmission in Africa. Lancet 362: 1792–1798. 14654317 10.1016/S0140-6736(03)14898-2

[68] Nakazawa Y, Williams R, Peterson AT, Mead P, Staples E, et al (2007) Climate change effects on Plague and Tularemia in the United States. Vector-Borne Zoonotic Dis 7: 529–540. 18047395 10.1089/vbz.2007.0125

[69] Saupe E, Papes M, Selden P, Vetter R (2011) Tracking a medically important spider: climate change, ecological niche modeling, and the Brown recluse (*Loxosceles reclusa*). PLoS One 6: e17731 doi:10.1371/journal.pone.0017731 21464985 10.1371/journal.pone.0017731 PMC3064576

[70] Roura-Pascual N, Suarez A, Gómez C, Pons P, Touyama Y, et al (2004) Geographical potential of Argentine ants (*Linepithema himule* Mays) in the face of global climate change. Proc R Soc Lond B 271: 2527–2534. 10.1098/rspb.2004.2898 PMC1691899 15615677

[71] Peterson AT, Martínez-Meyer E, González-Salazar C, Hall P (2004) Modeled climate change effects on distributions of Canadian butterfly species. Can J Zool 82: 851–858.

[72] Kearney M, Phillips B, Tracy CR, Christian K, Betts G, et al (2008) Modelling species distributions without using species distributions: the cane toad in Australia under current and future climates. Ecography 31: 423–434.

[73] Morueta-Holme N, Flojgaard C, Svenning JC (2010) Climate change risks and conservation implications for a threatened small-range mammal species. PLoS One 5: e10360 doi:10.1371/journal.pone.0010360 20454451 10.1371/journal.pone.0010360 PMC2861593

[74] Ureta C, Martínez-Meyer E, Perales H, Álvarez-Buylla E (2012) Projecting the effects of climate change on the distribution of maize races and their wild relatives in Mexico. Glob Change Biol 18: 1073–1082.

