# Supplementary figures and images for: Current and Future Niche of North and Central American Sand Flies (Diptera: Psychodidae) in Climate Change Scenarios
Source: PLoS Negl Trop Dis. 2013 Sep 19;7(9):e2421. doi: 10.1371/journal.pntd.0002421 (PMC3777871; doi:10.1371/journal.pntd.0002421)

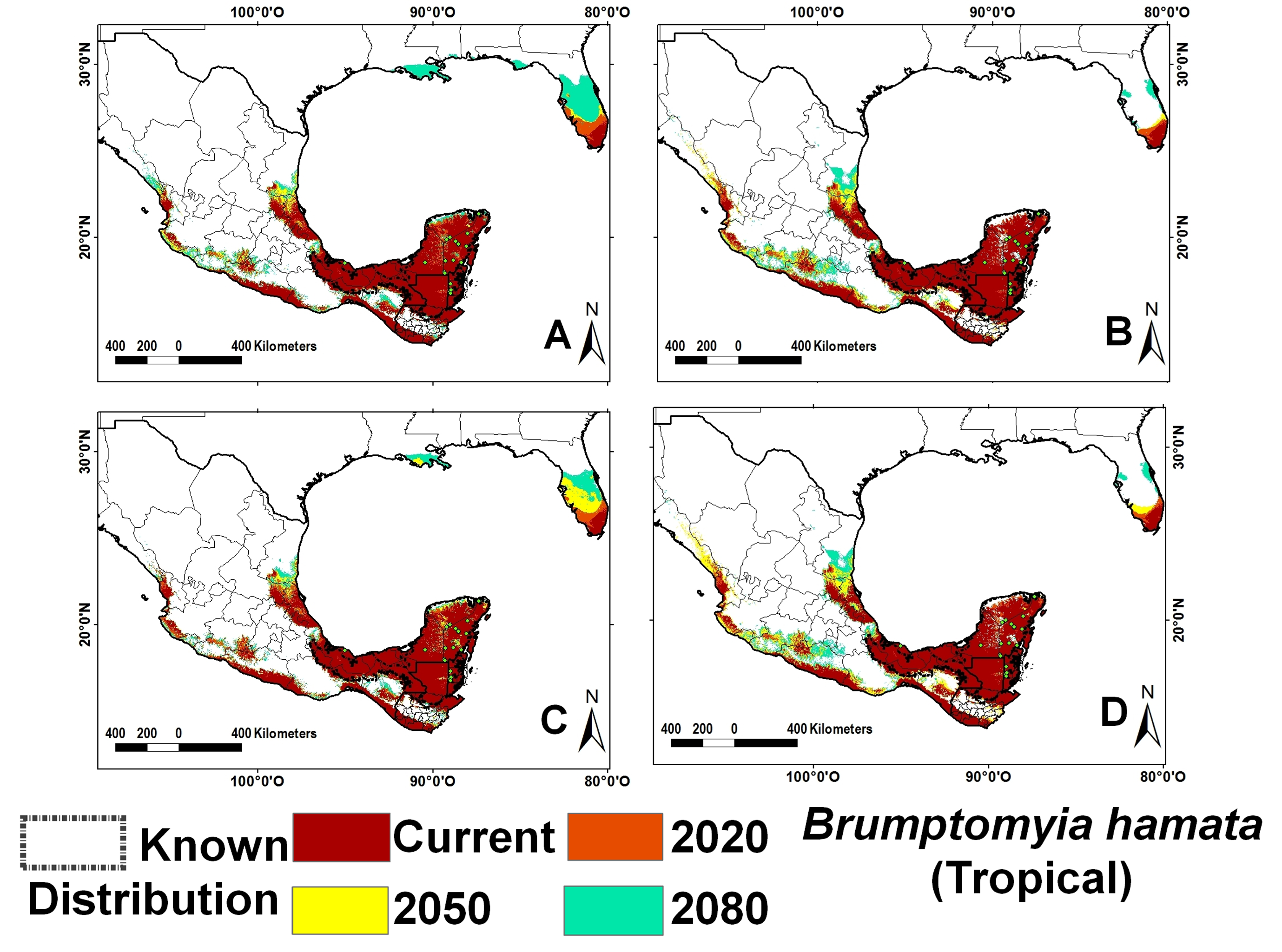

Supplement: Figure S1 — Ecological niche models for Brumptomyia hamata (tropical). A) A2 scenario, CSIRO model; B) A2 scenario, HadCM3 model; C) B2 scenario, CSIRO model and D) B2 scenario, HadCM3 model. (TIF) [file pntd.0002421.s001.tif]

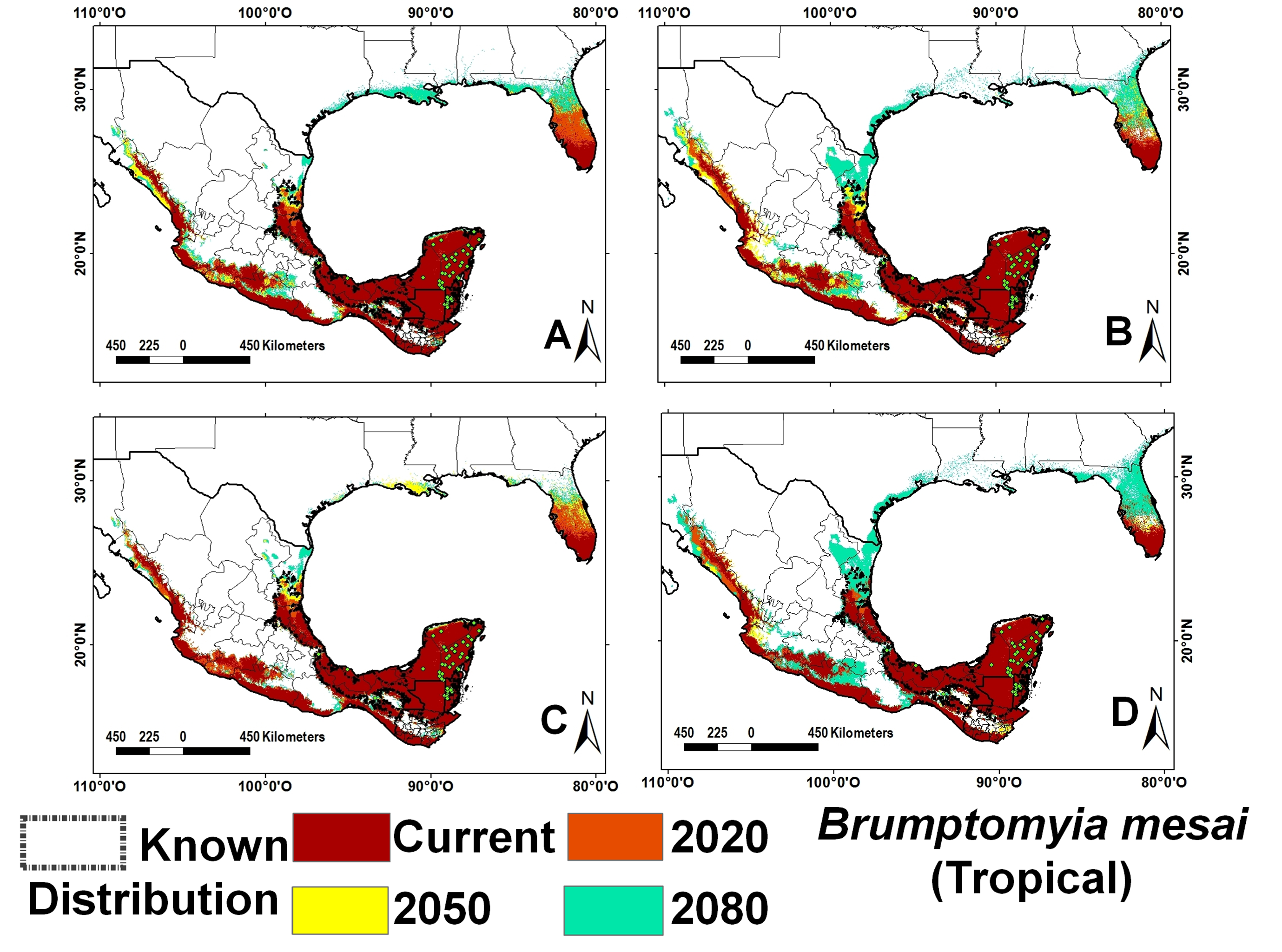

Supplement: Figure S2 — Ecological niche models for Brumptomyia mesai (tropical). A) A2 scenario, CSIRO model; B) A2 scenario, HadCM3 model; C) B2 scenario, CSIRO model and D) B2 scenario, HadCM3 model. (TIF) [file pntd.0002421.s002.tif]

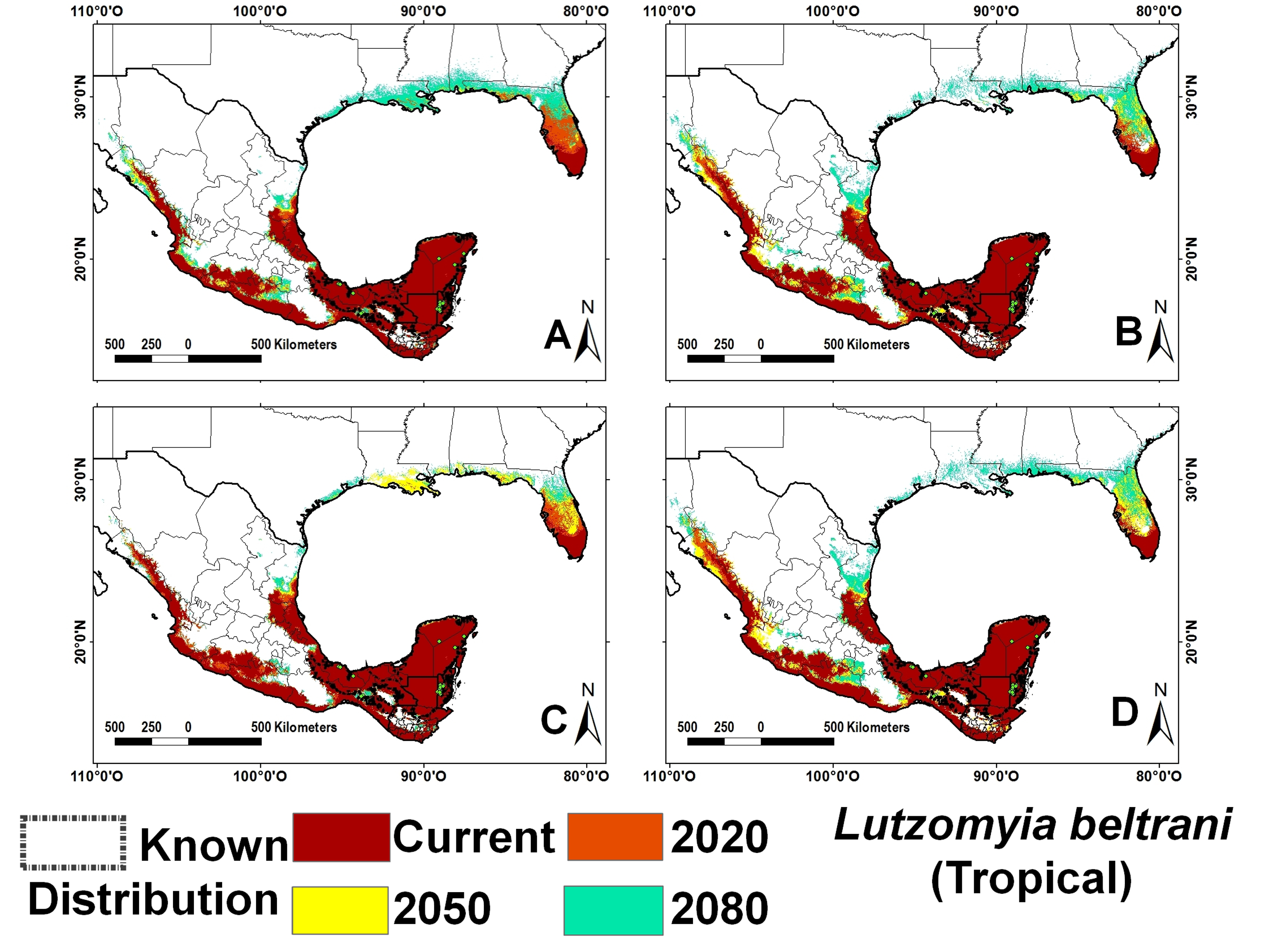

Supplement: Figure S3 — Ecological niche models for Lutzomyia beltrani (tropical). A) A2 scenario, CSIRO model; B) A2 scenario, HadCM3 model; C) B2 scenario, CSIRO model and D) B2 scenario, HadCM3 model. (TIF) [file pntd.0002421.s003.tif]

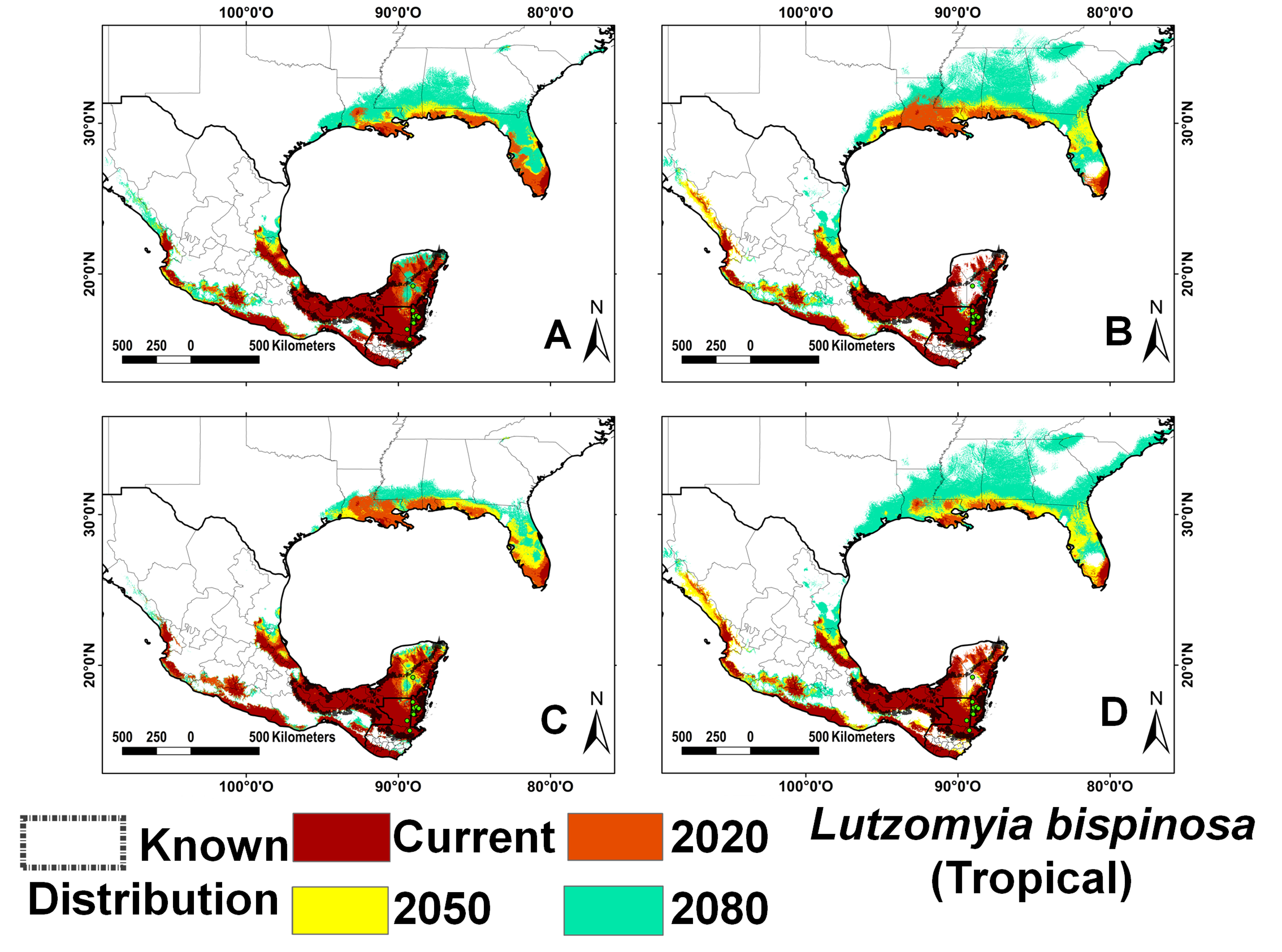

Supplement: Figure S4 — Ecological niche models for Lutzomyia bispinosa (tropical). A) A2 scenario, CSIRO model; B) A2 scenario, HadCM3 model; C) B2 scenario, CSIRO model and D) B2 scenario, HadCM3 model. (TIF) [file pntd.0002421.s004.tif]

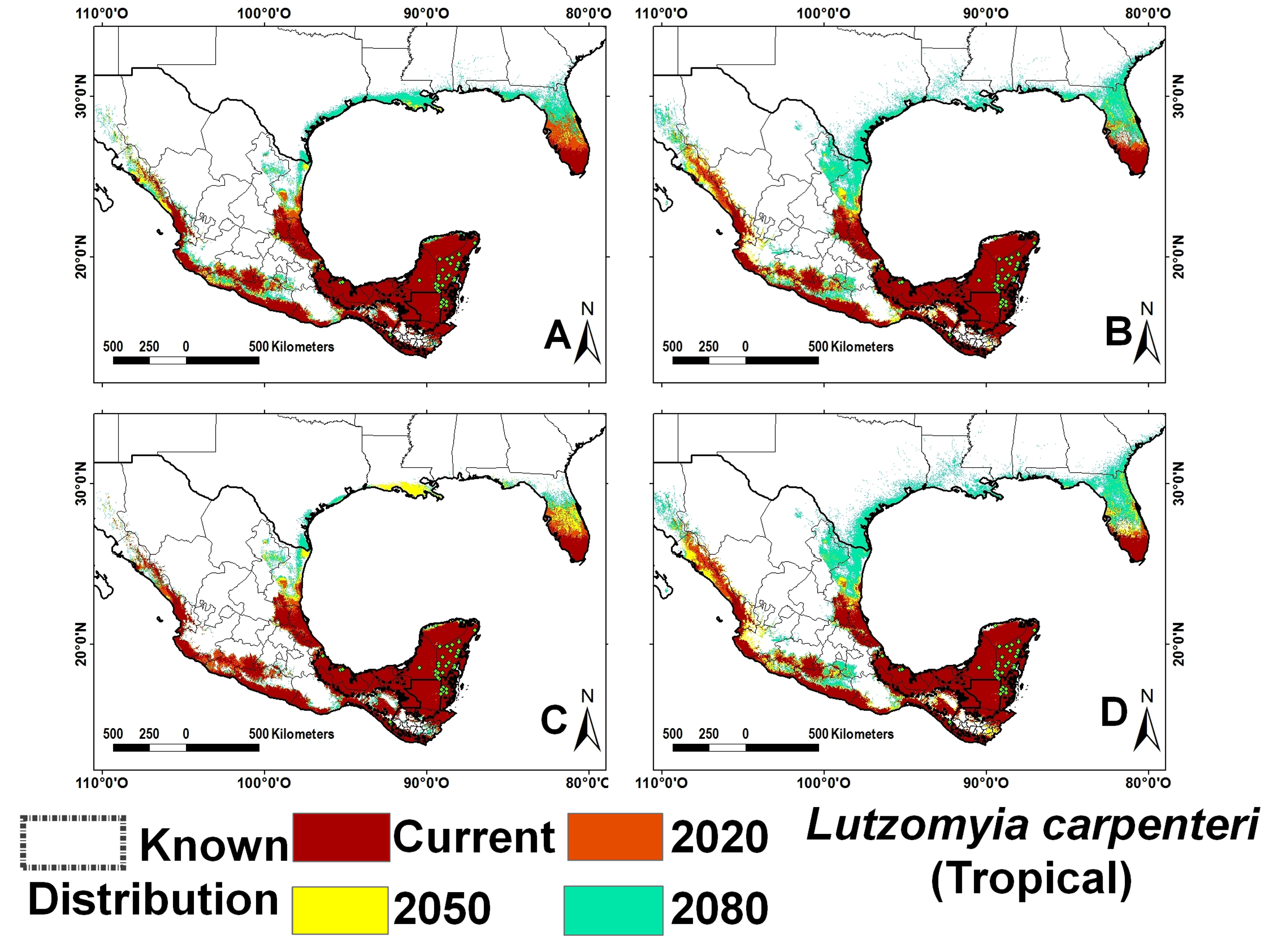

Supplement: Figure S5 — Ecological niche models for Lutzomyia carpenteri (tropical). A) A2 scenario, CSIRO model; B) A2 scenario, HadCM3 model; C) B2 scenario, CSIRO model and D) B2 scenario, HadCM3 model. (TIF) [file pntd.0002421.s005.tif]

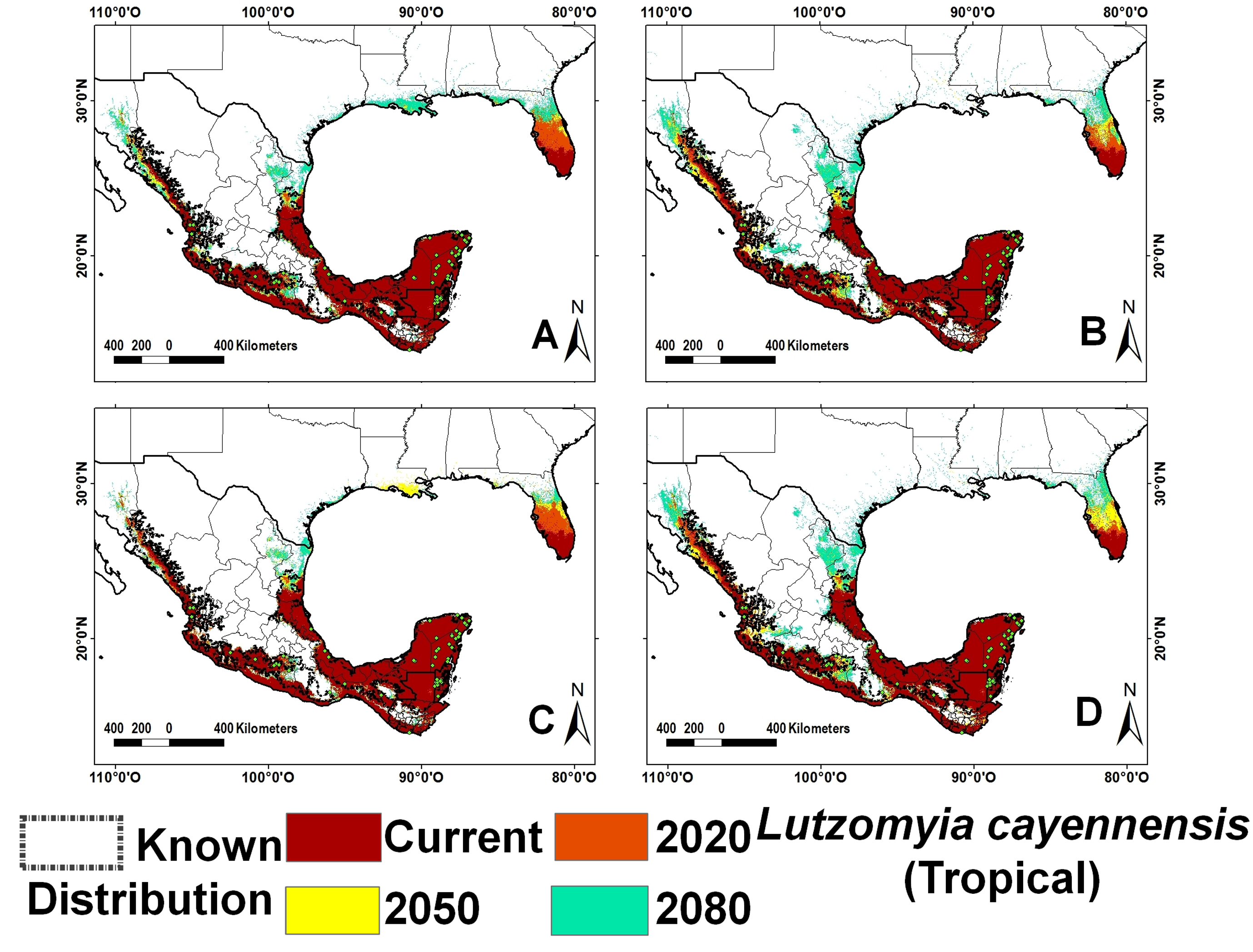

Supplement: Figure S6 — Ecological niche models for Lutzomyia cayennensis (tropical). A) A2 scenario, CSIRO model; B) A2 scenario, HadCM3 model; C) B2 scenario, CSIRO model and D) B2 scenario, HadCM3 model. (TIF) [file pntd.0002421.s006.tif]

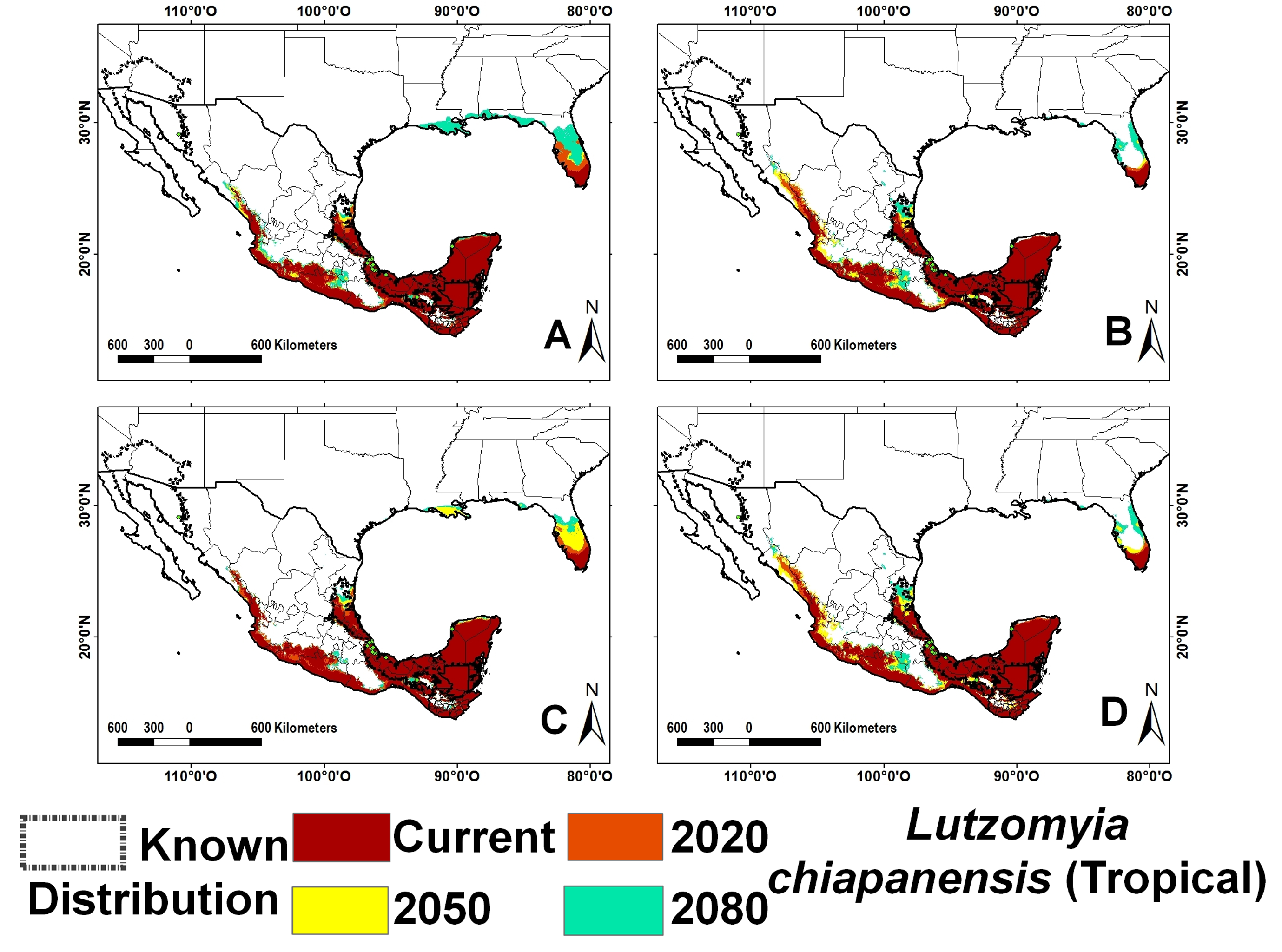

Supplement: Figure S7 — Ecological niche models for Lutzomyia chiapanensis (tropical). A) A2 scenario, CSIRO model; B) A2 scenario, HadCM3 model; C) B2 scenario, CSIRO model and D) B2 scenario, HadCM3 model. (TIF) [file pntd.0002421.s007.tif]

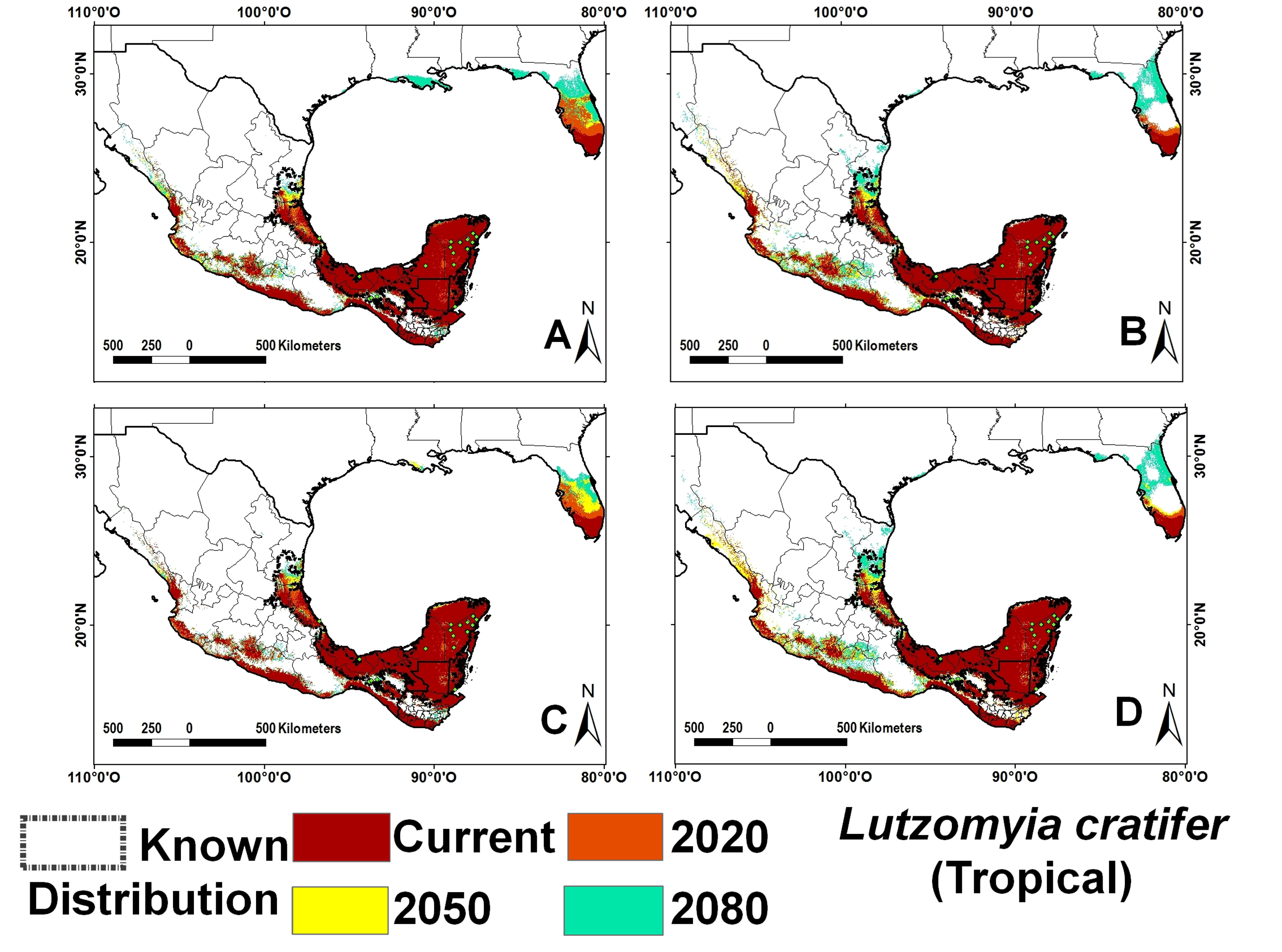

Supplement: Figure S8 — Ecological niche models for Lutzomyia cratifer (tropical). A) A2 scenario, CSIRO model; B) A2 scenario, HadCM3 model; C) B2 scenario, CSIRO model and D) B2 scenario, HadCM3 model. (TIF) [file pntd.0002421.s008.tif]

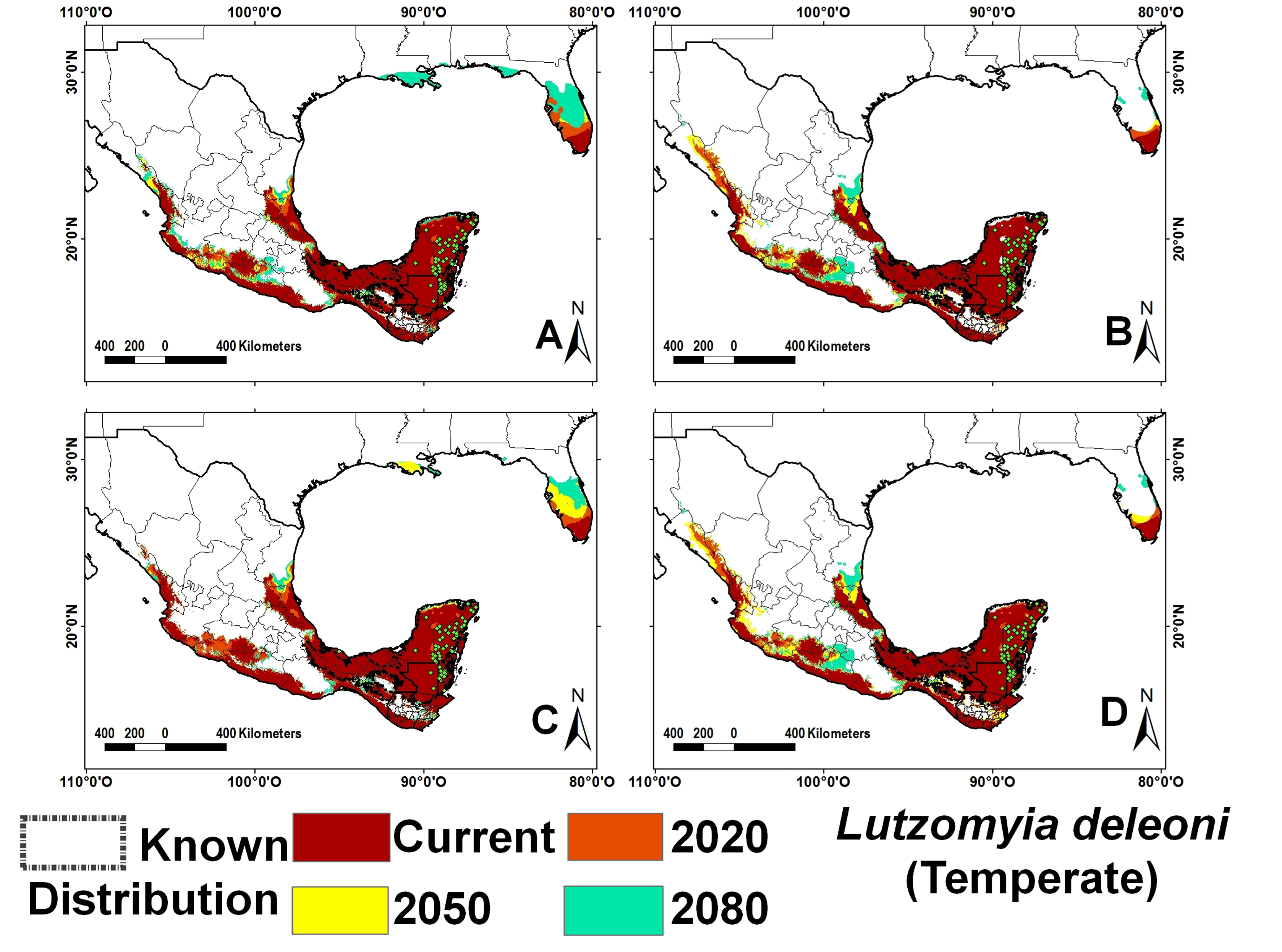

Supplement: Figure S9 — Ecological niche models for Lutzomyia deleoni (tropical). A) A2 scenario, CSIRO model; B) A2 scenario, HadCM3 model; C) B2 scenario, CSIRO model and D) B2 scenario, HadCM3 model. (TIF) [file pntd.0002421.s009.tif]

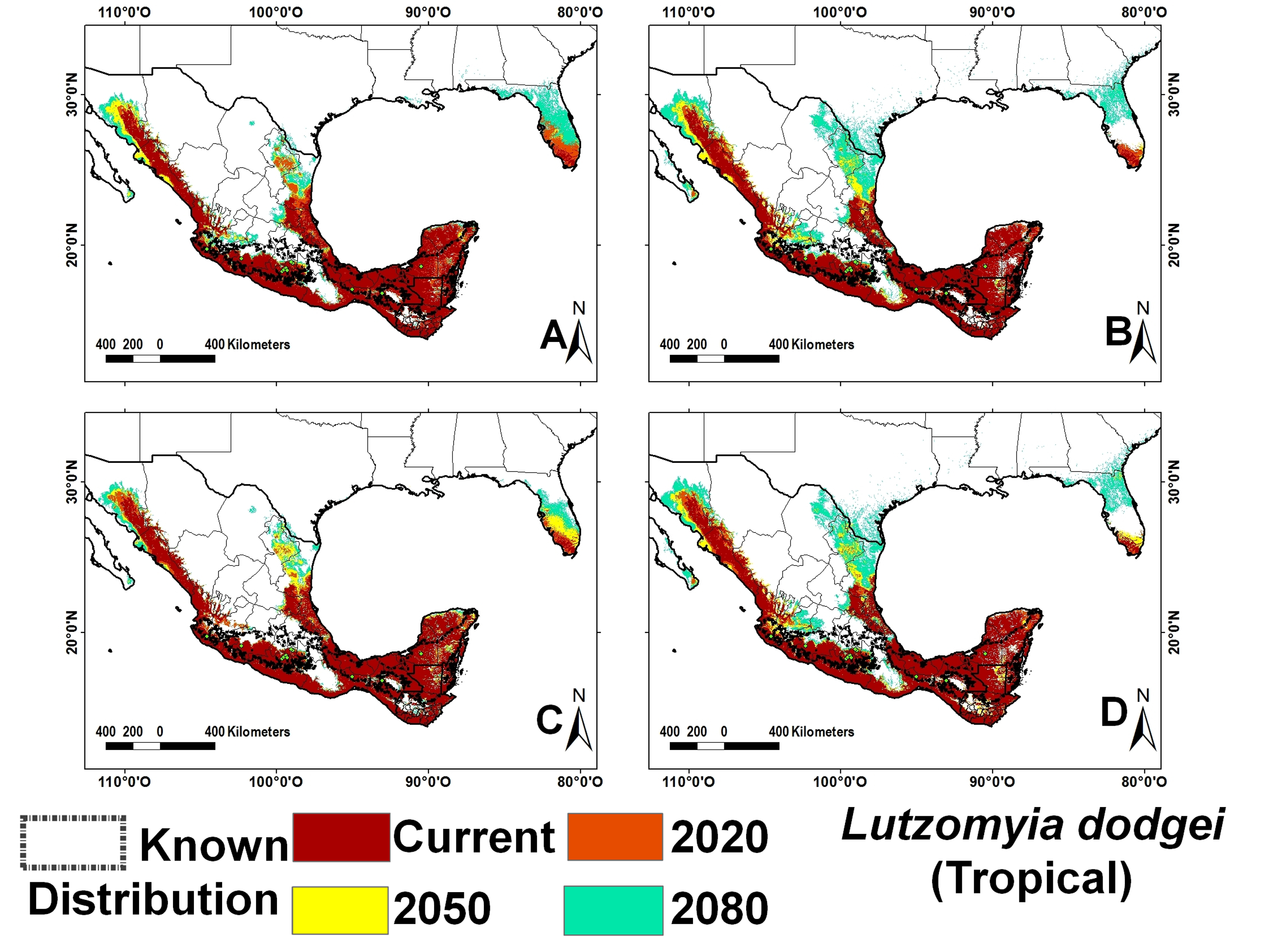

Supplement: Figure S10 — Ecological niche models for Lutzomyia dodgei (tropical). A) A2 scenario, CSIRO model; B) A2 scenario, HadCM3 model; C) B2 scenario, CSIRO model and D) B2 scenario, HadCM3 model. (TIF) [file pntd.0002421.s010.tif]

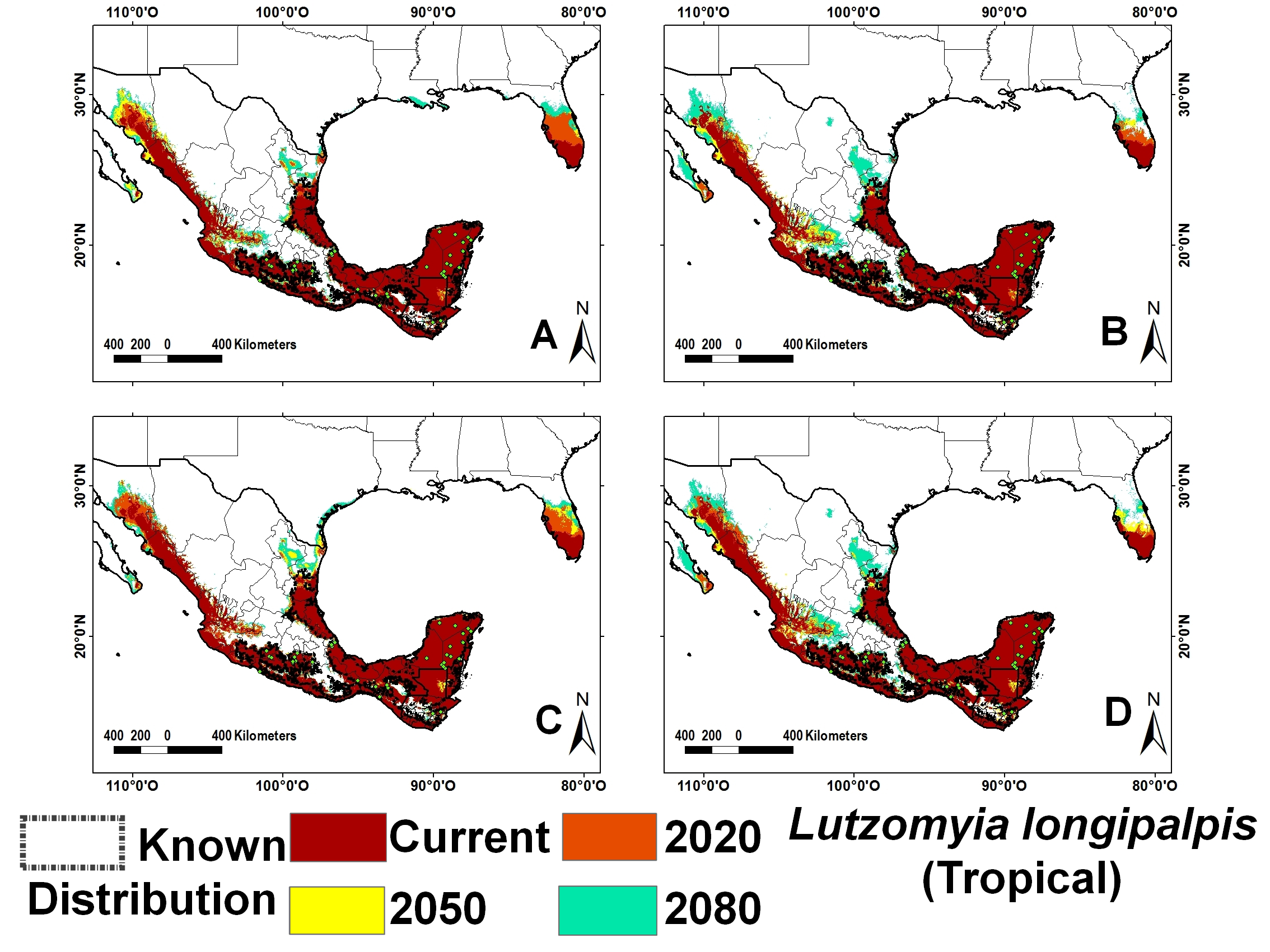

Supplement: Figure S11 — Ecological niche models for Lutzomyia longipalpis (tropical). A) A2 scenario, CSIRO model; B) A2 scenario, HadCM3 model; C) B2 scenario, CSIRO model and D) B2 scenario, HadCM3 model. (TIF) [file pntd.0002421.s011.tif]

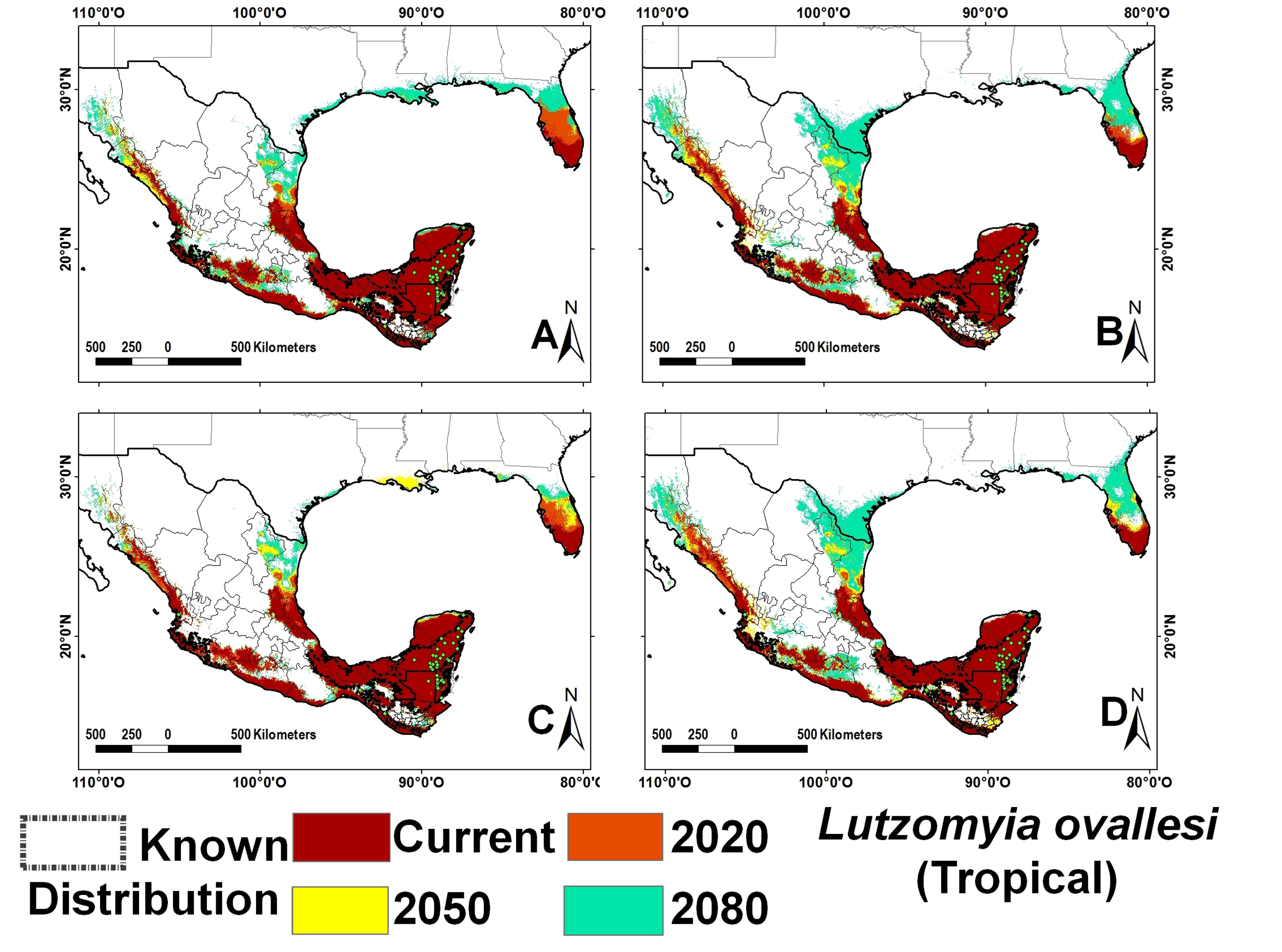

Supplement: Figure S12 — Ecological niche models for Lutzomyia ovallesi (tropical). A) A2 scenario, CSIRO model; B) A2 scenario, HadCM3 model; C) B2 scenario, CSIRO model and D) B2 scenario, HadCM3 model. (TIF) [file pntd.0002421.s012.tif]

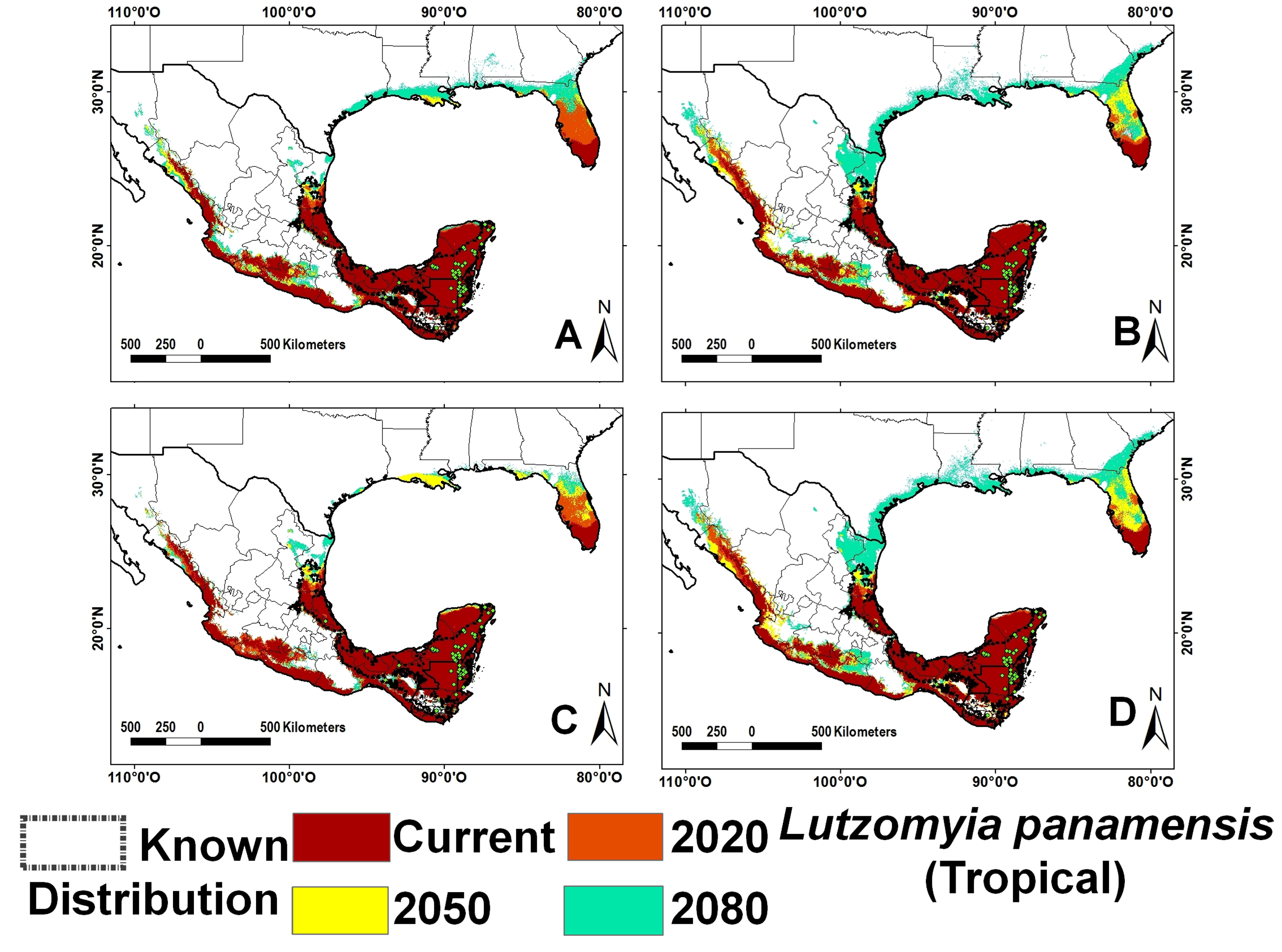

Supplement: Figure S13 — Ecological niche models for Lutzomyia panamensis (tropical). A) A2 scenario, CSIRO model; B) A2 scenario, HadCM3 model; C) B2 scenario, CSIRO model and D) B2 scenario, HadCM3 model. (TIF) [file pntd.0002421.s013.tif]

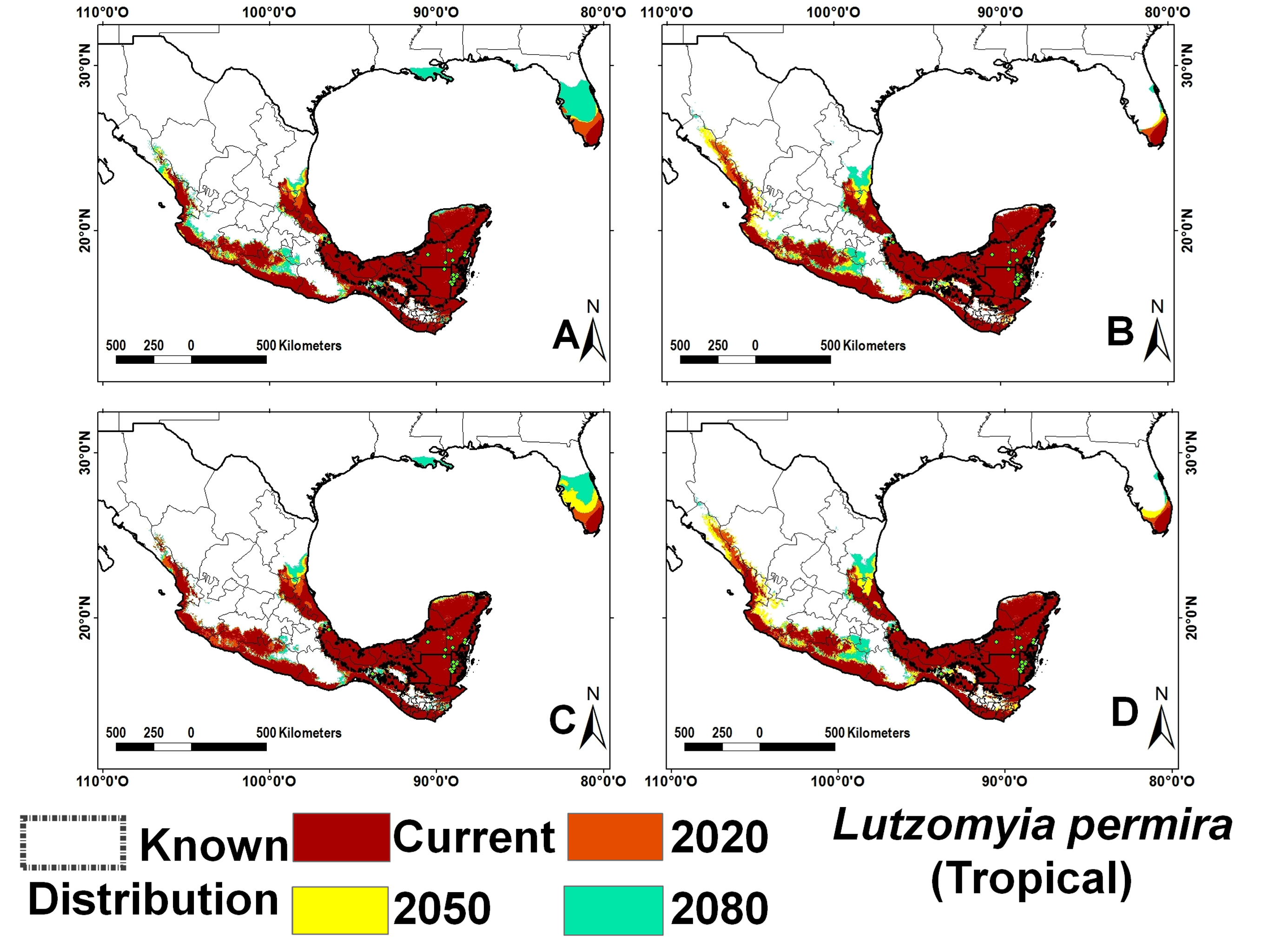

Supplement: Figure S14 — Ecological niche models for Lutzomyia permira (tropical). A) A2 scenario, CSIRO model; B) A2 scenario, HadCM3 model; C) B2 scenario, CSIRO model and D) B2 scenario, HadCM3 model. (TIF) [file pntd.0002421.s014.tif]

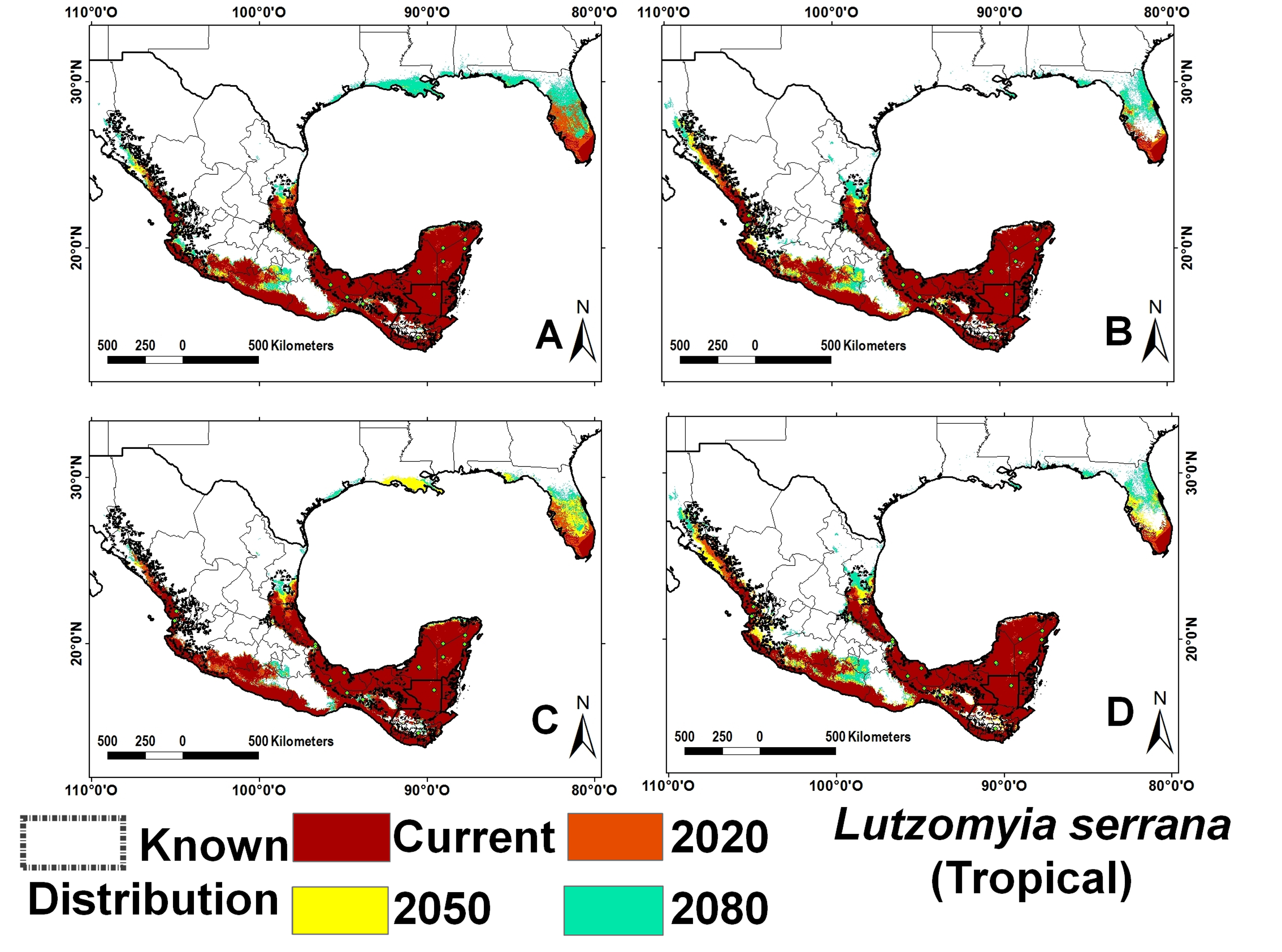

Supplement: Figure S15 — Ecological niche models for Lutzomyia serrana (tropical). A) A2 scenario, CSIRO model; B) A2 scenario, HadCM3 model; C) B2 scenario, CSIRO model and D) B2 scenario, HadCM3 model. (TIF) [file pntd.0002421.s015.tif]

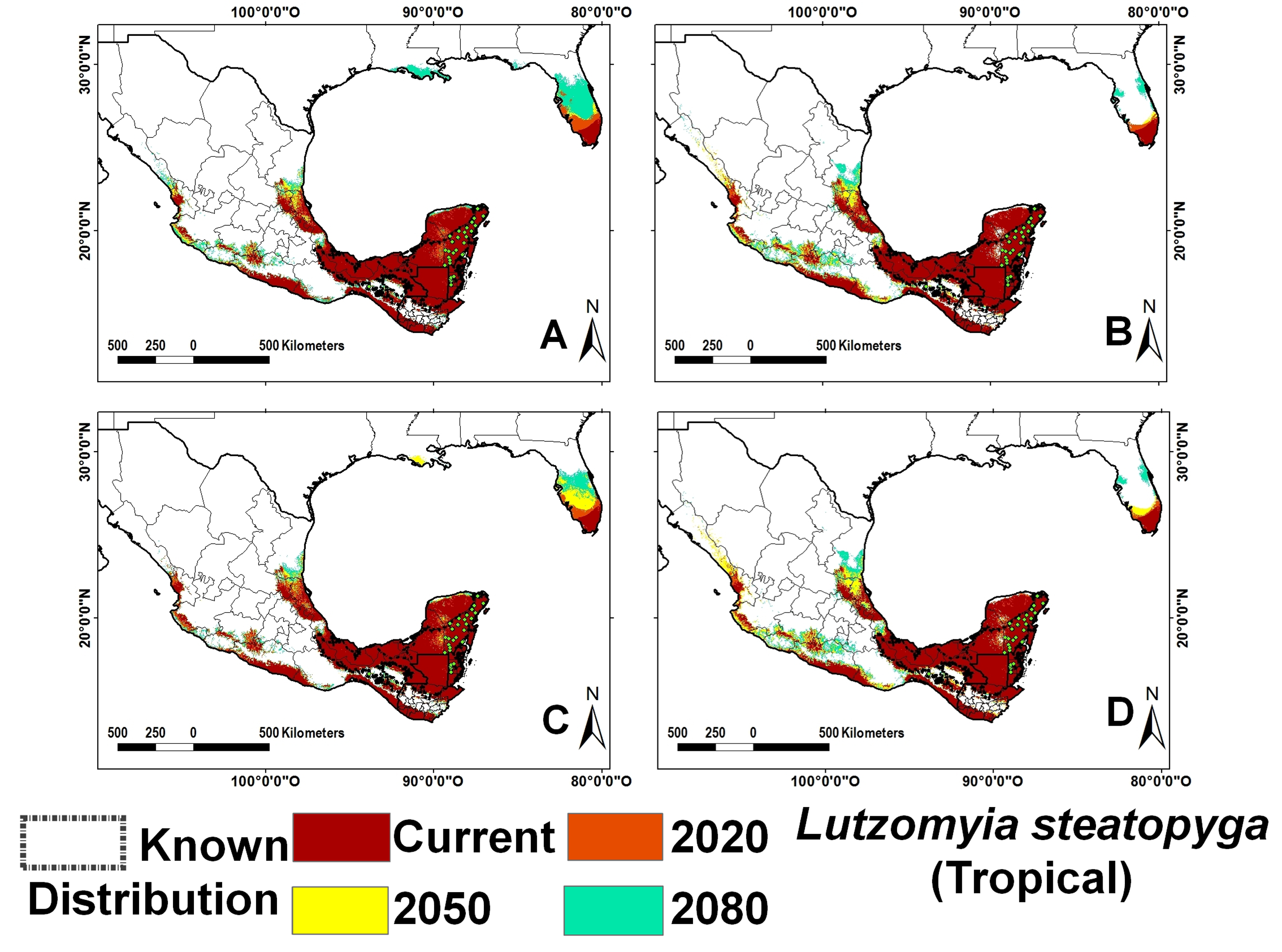

Supplement: Figure S16 — Ecological niche models for Lutzomyia steatopyga (tropical). A) A2 scenario, CSIRO model; B) A2 scenario, HadCM3 model; C) B2 scenario, CSIRO model and D) B2 scenario, HadCM3 model. (TIF) [file pntd.0002421.s016.tif]

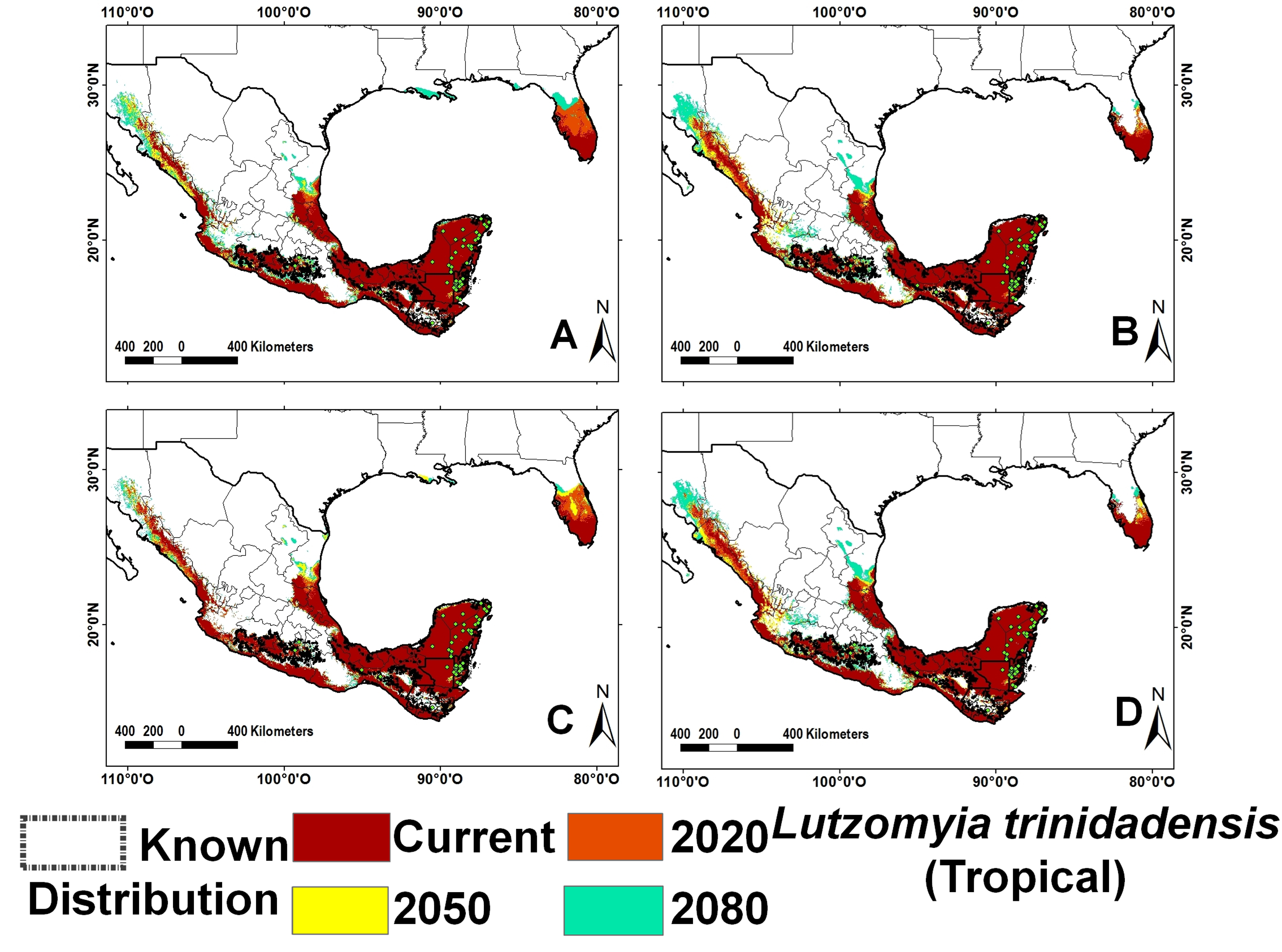

Supplement: Figure S17 — Ecological niche models for Lutzomyia trinidadensis (tropical). A) A2 scenario, CSIRO model; B) A2 scenario, HadCM3 model; C) B2 scenario, CSIRO model and D) B2 scenario, HadCM3 model. (TIF) [file pntd.0002421.s017.tif]

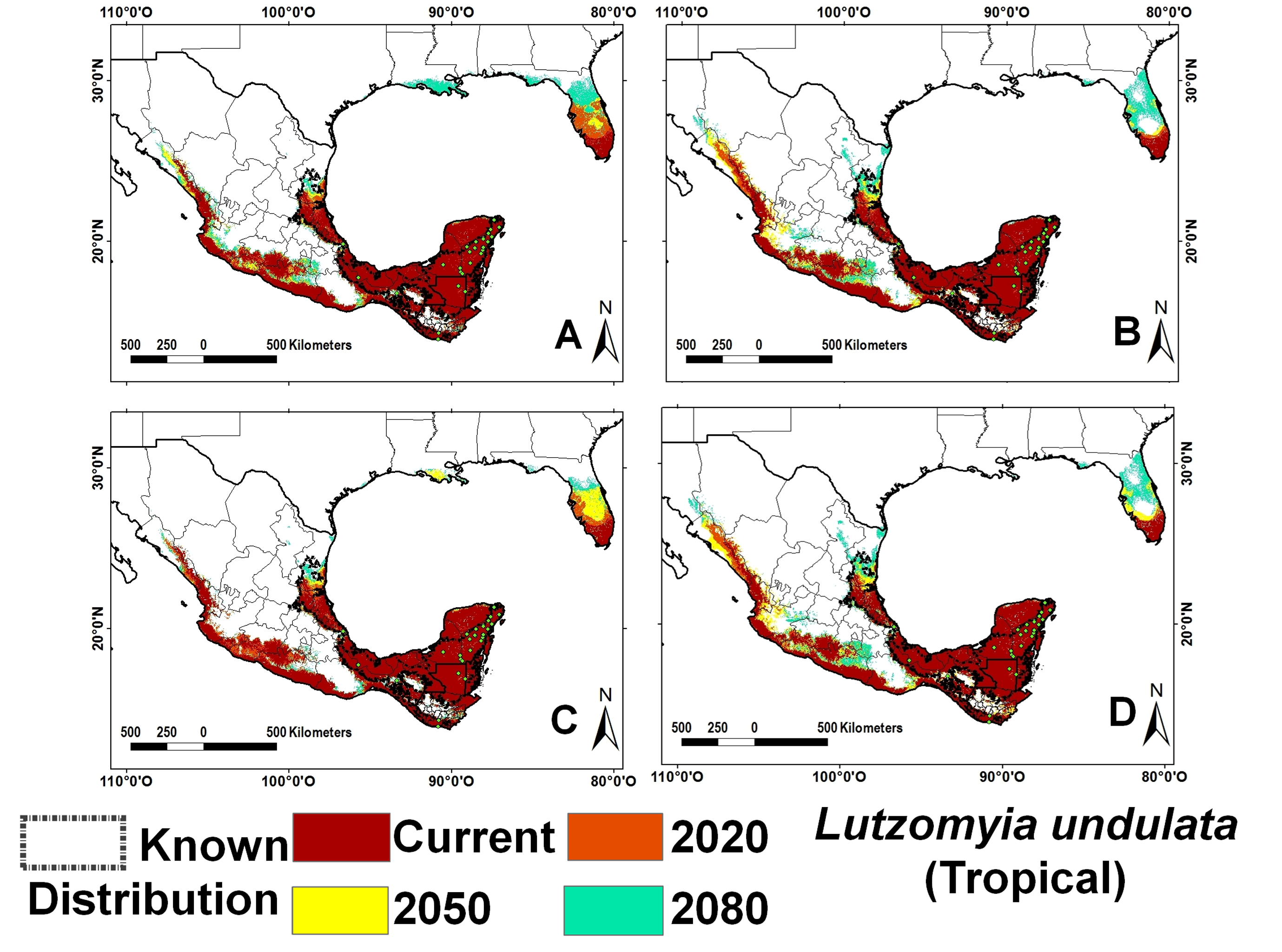

Supplement: Figure S18 — Ecological niche models for Lutzomyia undulata (tropical). A) A2 scenario, CSIRO model; B) A2 scenario, HadCM3 model; C) B2 scenario, CSIRO model and D) B2 scenario, HadCM3 model. (TIF) [file pntd.0002421.s018.tif]

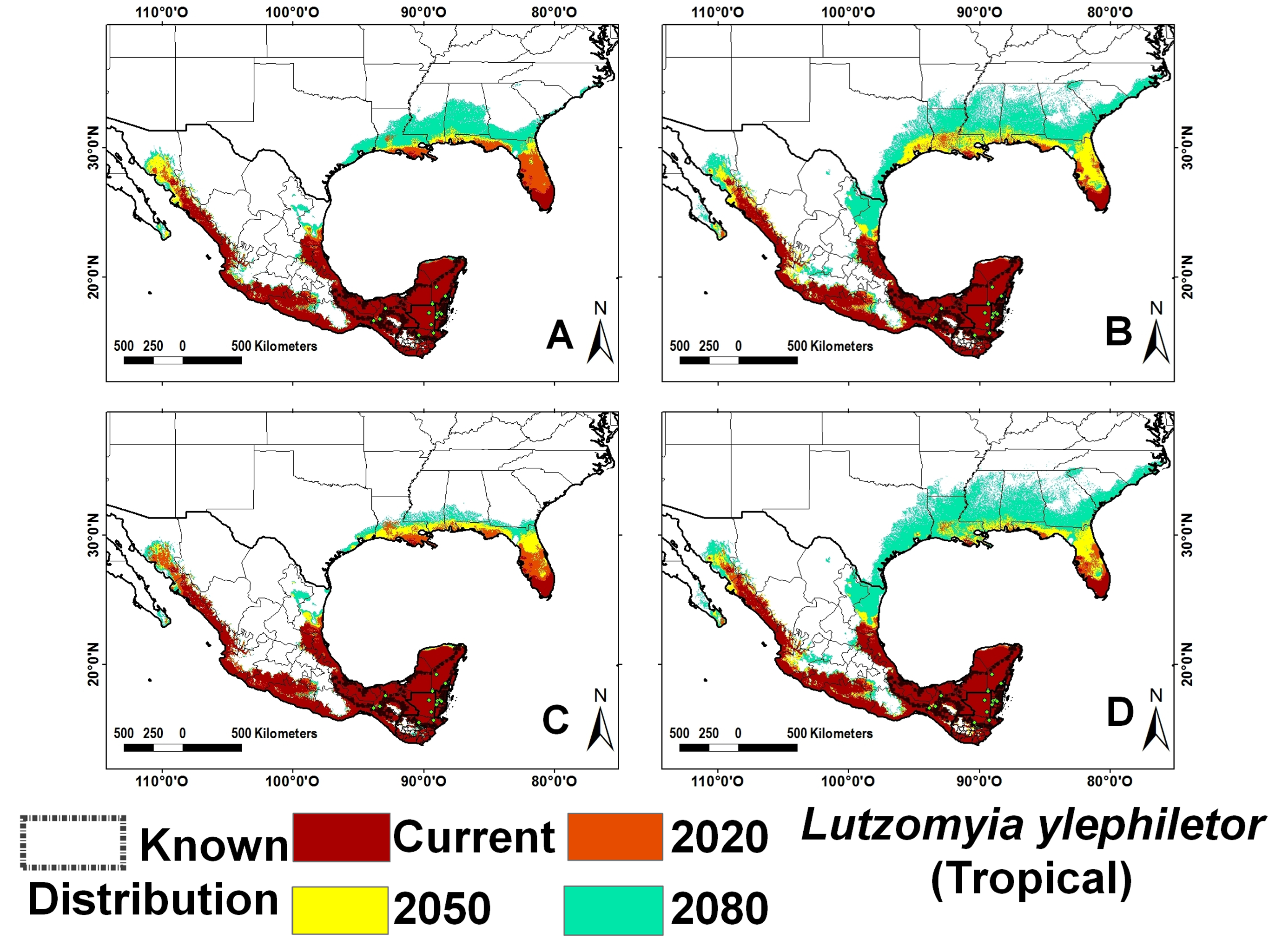

Supplement: Figure S19 — Ecological niche models for Lutzomyia ylephiletor (tropical). A) A2 scenario, CSIRO model; B) A2 scenario, HadCM3 model; C) B2 scenario, CSIRO model and D) B2 scenario, HadCM3 model. (TIF) [file pntd.0002421.s019.tif]

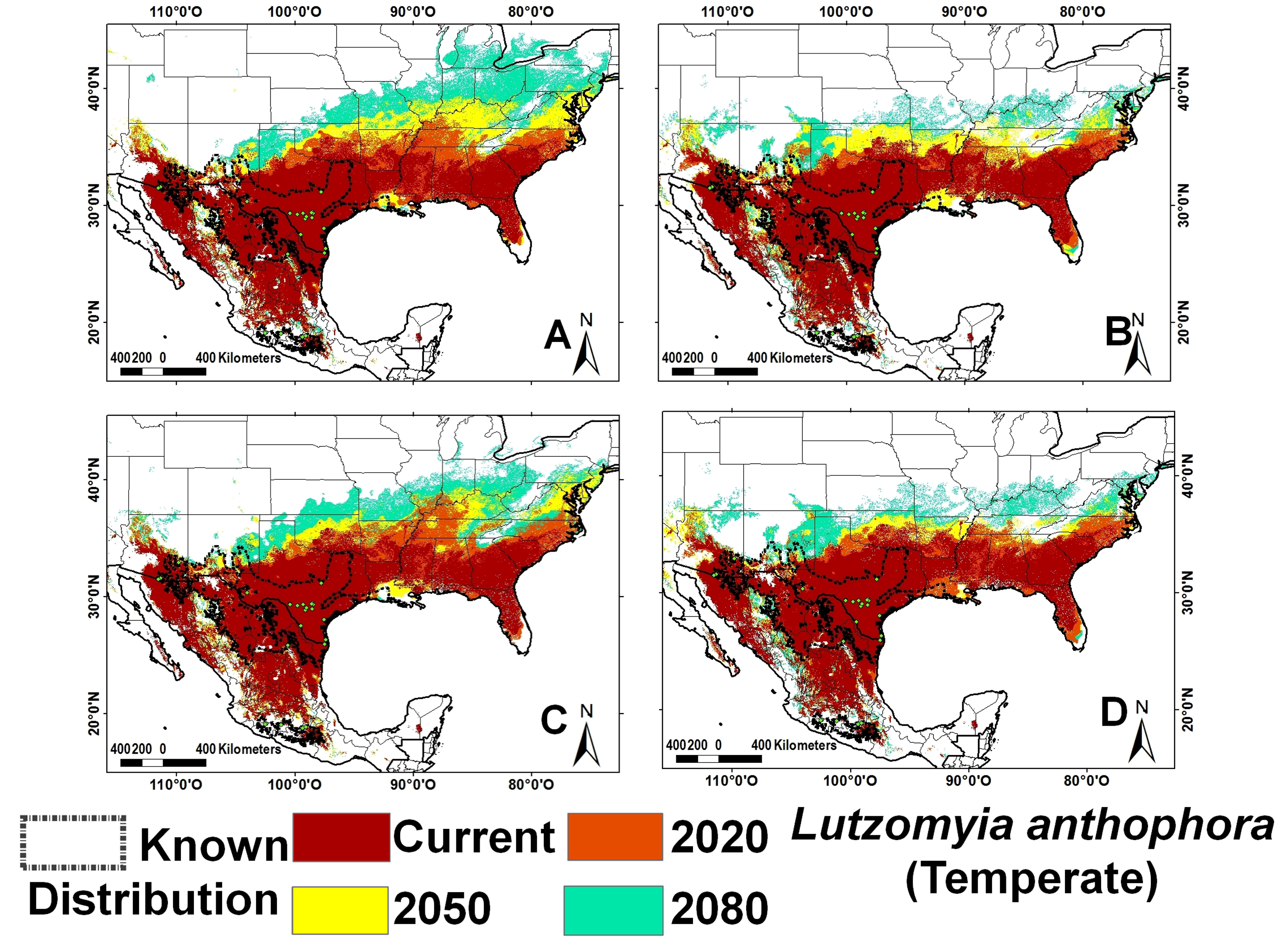

Supplement: Figure S20 — Ecological niche models for Lutzomyia anthophora (temperate). A) A2 scenario, CSIRO model; B) A2 scenario, HadCM3 model; C) B2 scenario, CSIRO model and D) B2 scenario, HadCM3 model. (TIF) [file pntd.0002421.s020.tif]

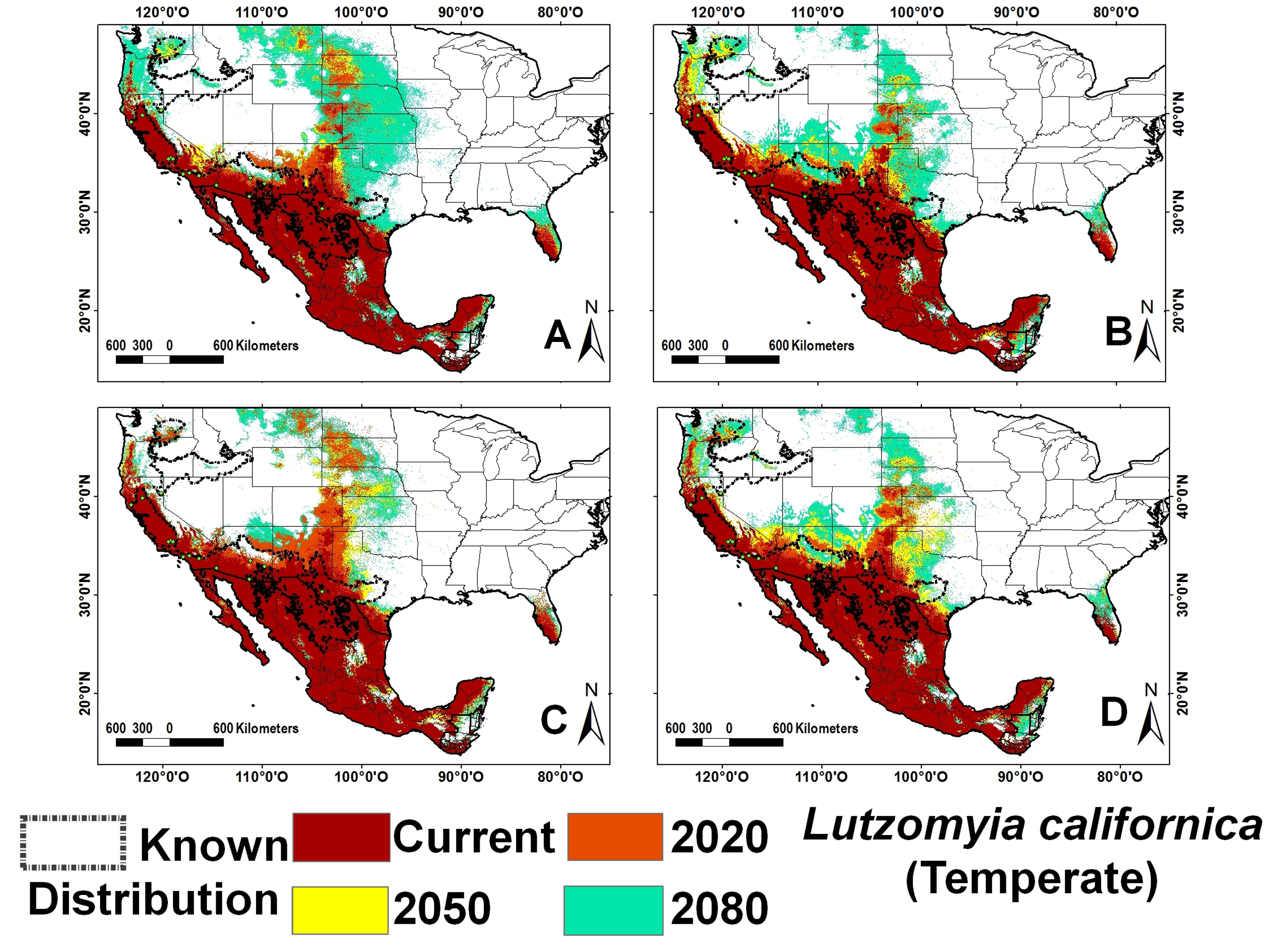

Supplement: Figure S21 — Ecological niche models for Lutzomyia californica (temperate). A) A2 scenario, CSIRO model; B) A2 scenario, HadCM3 model; C) B2 scenario, CSIRO model and D) B2 scenario, HadCM3 model. (TIF) [file pntd.0002421.s021.tif]

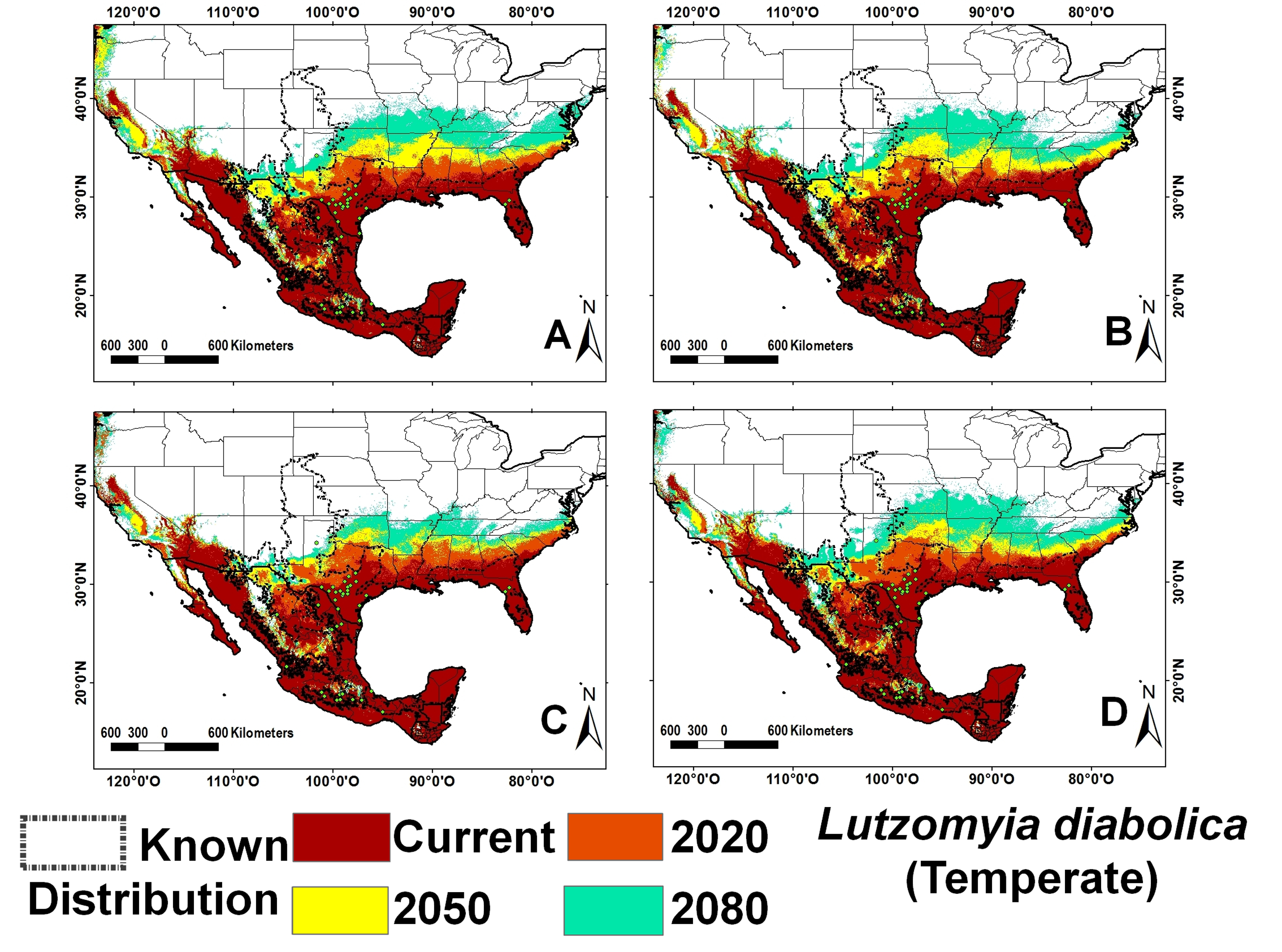

Supplement: Figure S22 — Ecological niche models for Lutzomyia diabolica (temperate). A) A2 scenario, CSIRO model; B) A2 scenario, HadCM3 model; C) B2 scenario, CSIRO model and D) B2 scenario, HadCM3 model. (TIF) [file pntd.0002421.s022.tif]

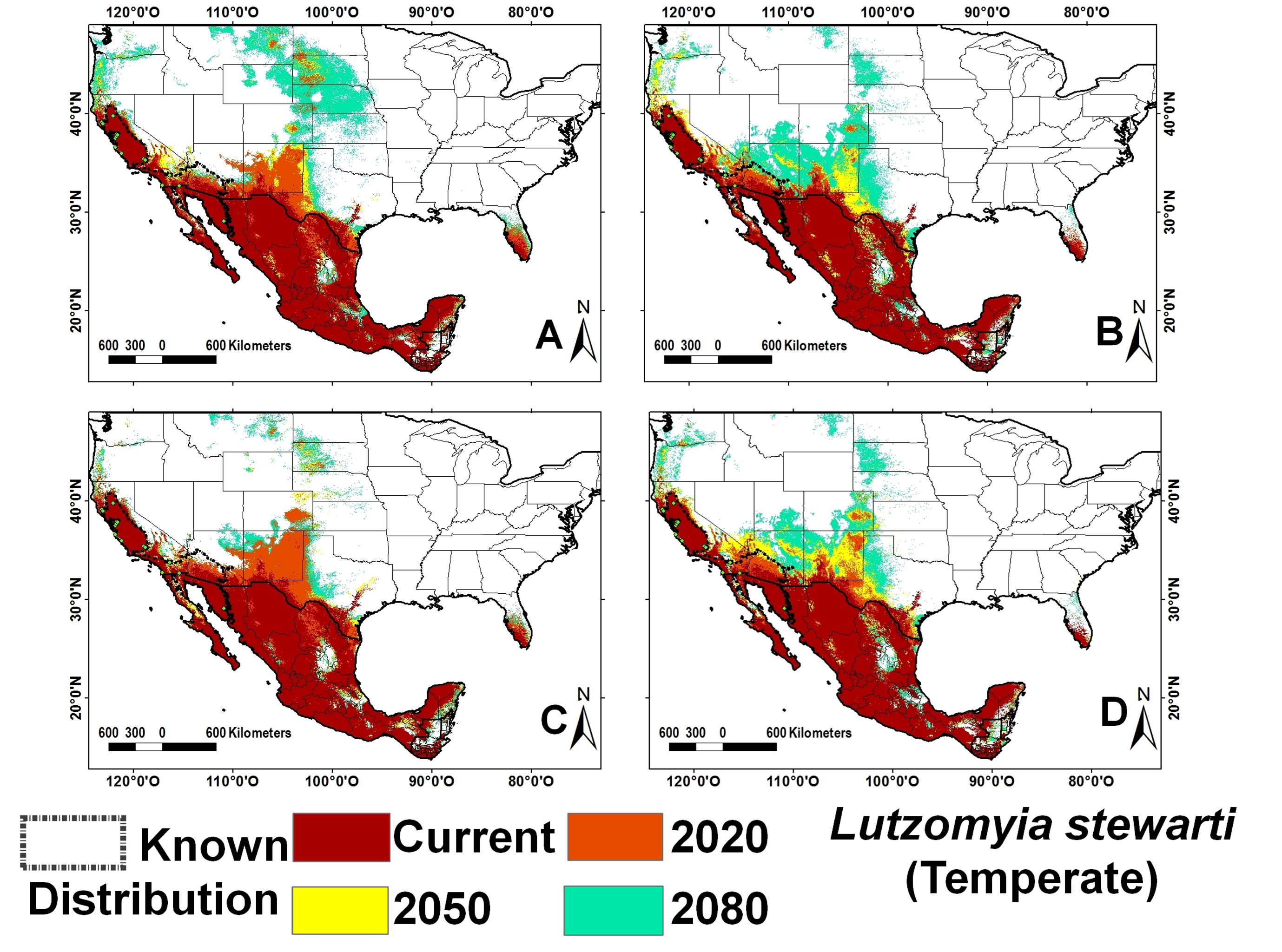

Supplement: Figure S23 — Ecological niche models for Lutzomyia stewarti (temperate). A) A2 scenario, CSIRO model; B) A2 scenario, HadCM3 model; C) B2 scenario, CSIRO model and D) B2 scenario, HadCM3 model. (TIF) [file pntd.0002421.s023.tif]

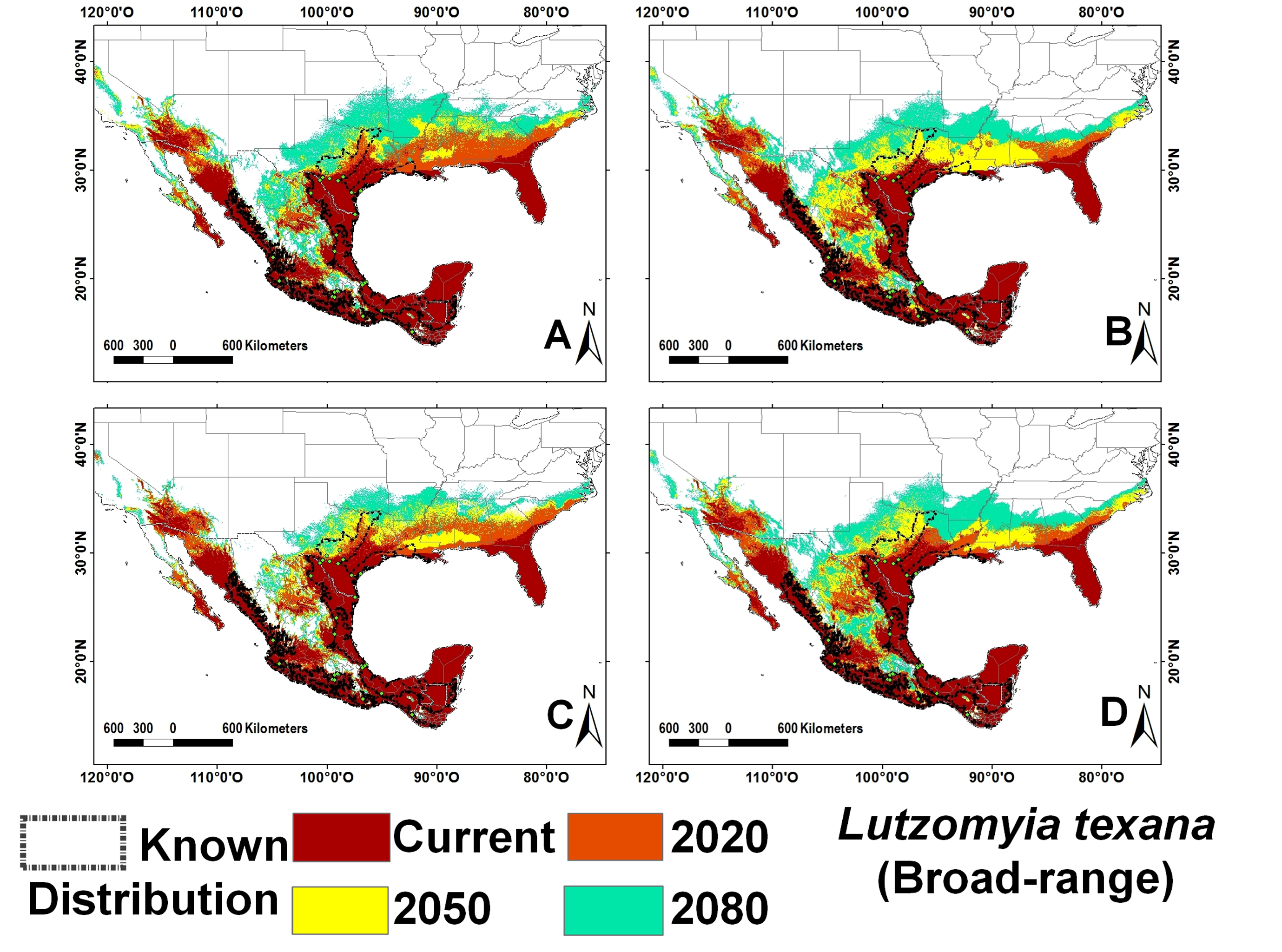

Supplement: Figure S24 — Ecological niche models for Lutzomyia texana (broad-range). A) A2 scenario, CSIRO model; B) A2 scenario, HadCM3 model; C) B2 scenario, CSIRO model and D) B2 scenario, HadCM3 model. (TIF) [file pntd.0002421.s024.tif]

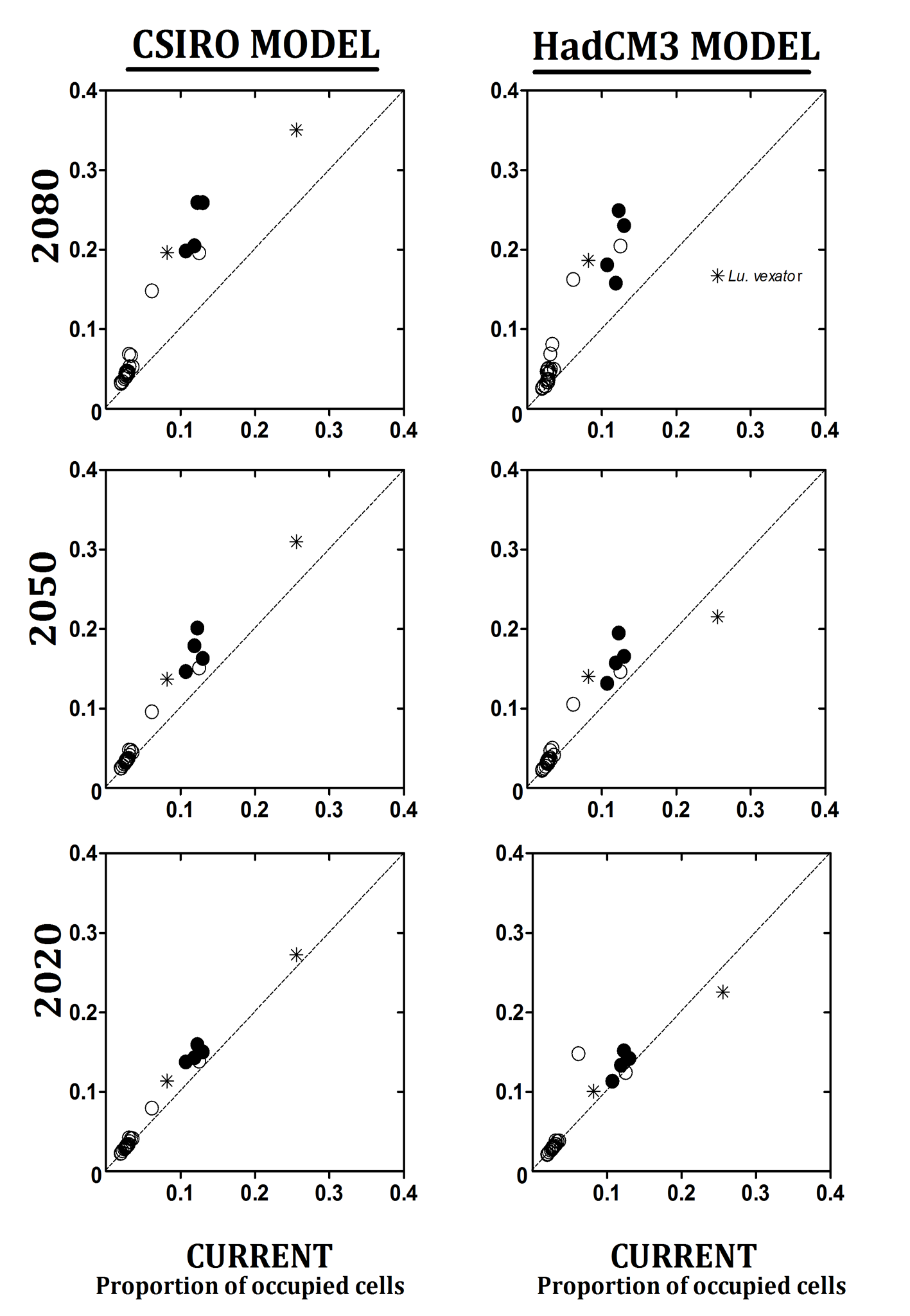

Supplement: Figure S25 — Proportion of pixels for sand fly ENM from current to 2080 in the A2 scenario. Predicted future scenarios using the CSIRO model (left) and HadCM3 model (right). Empty circles = tropical species; solid dot = temperate species; asterisk = broad-range species. (TIF) [file pntd.0002421.s025.tif]

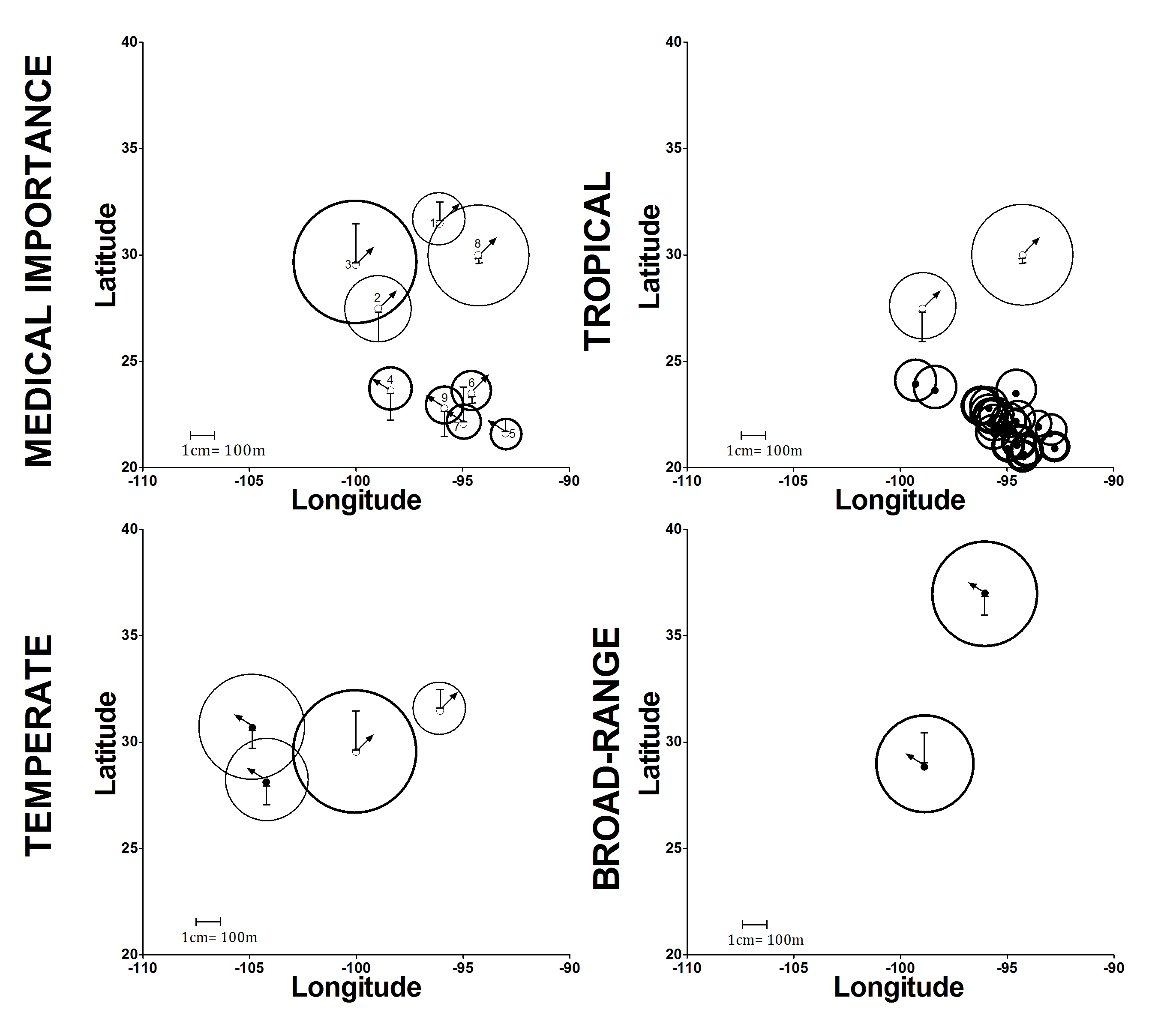

Supplement: Figure S26 — Changes in niche breadth, ENM centroid and elevational range in A2 scenario. The circle size represents the proportional distribution change, while the thickness is proportional to the overlap percentage between current and 2050 projections (border thickness of 0.5 pt = 25–50%; 1 pt = 51–75%; 2 pt = 76–100%). The direction of the arrow represents the direction of change of the centroid position and its size represents its magnitude. The elevational range changes are represented by the bars (bar up = increase; bar below = decrease). Epidemiologically important species are: 1) Lu. anthophora, 2) Lu. cruciata, 3) Lu. diabolica, 4) Lu. longipalpis, 5) Lu. olmeca olmeca, 6) Lu. ovallesi, 7) Lu. panamensis, 8) Lu. shannoni and 9) Lu. ylephiletor. (TIF) [file pntd.0002421.s026.tif]
