# Supplementary material for: Current and Future Niche of North and Central American Sand Flies (Diptera: Psychodidae) in Climate Change Scenarios
Source: PLoS Negl Trop Dis. 2013 Sep 19;7(9):e2421. doi: 10.1371/journal.pntd.0002421 (PMC3777871; doi:10.1371/journal.pntd.0002421)
Supplement: Table S1 — Reference points for sand fly collection records used in ENM modeling of NCA species. (PDF) [file pntd.0002421.s027.pdf]

Table S1. Reference points for sand fly collection records used in ENM modeling of NCA species

Genus	Species	Community	Longitude	Latitude	State	Country	References
Lutzomyia	*longipalpis*	Agua Zarca	-99.3619444444	18.2363888889	Guerrero	MEXICO	20
Lutzomyia	*shannoni*	Aguada La Muñeca	-90.8381250000	18.5999710000	Campeche	MEXICO	5
Lutzomyia	*panamensis*	Aguada La Muñeca	-90.8381250000	18.5999710000	Campeche	MEXICO	4
Lutzomyia	*cruciata*	Aguada La Muñeca	-90.8381250000	18.5999710000	Campeche	MEXICO	4
Lutzomyia	*carpenteri*	Altos de Sevilla	-88.6671014670	18.8409818897	Quintana Roo	MEXICO	58
Lutzomyia	*deleoni*	Altos de Sevilla	-88.6671014670	18.8409818897	Quintana Roo	MEXICO	58
Brumptomyia	*mesai*	Altos de Sevilla	-88.6671014670	18.8409818897	Quintana Roo	MEXICO	58
Lutzomyia	*olmeca*	Altos de Sevilla	-88.6671014670	18.8409818897	Quintana Roo	MEXICO	58
Lutzomyia	*panamensis*	Altos de Sevilla	-88.6671014670	18.8409818897	Quintana Roo	MEXICO	58
Lutzomyia	*shannoni*	Altos de Sevilla	-88.6671014670	18.8409818897	Quintana Roo	MEXICO	58

Lutzomyia	*steatopyga*	Altos de Sevilla	-88.6671014670	18.8409818897	Quintana Roo	MEXICO	58
Lutzomyia	*ovallesi*	Altos de Sevilla	-88.6671014670	18.8409818897	Quintana Roo	MEXICO	58
Lutzomyia	*deleoni*	Altos de Sevilla	-88.6681130443	18.8377203097	Quintana Roo	MEXICO	58
Brumptomyia	*mesai*	Altos de Sevilla	-88.6681130443	18.8377203097	Quintana Roo	MEXICO	58
Lutzomyia	*panamensis*	Altos de Sevilla	-88.6681130443	18.8377203097	Quintana Roo	MEXICO	58
Lutzomyia	*steatopyga*	Altos de Sevilla	-88.6681130443	18.8377203097	Quintana Roo	MEXICO	58
Lutzomyia	*olmeca*	Altos de Sevilla	-88.6681130443	18.8377203097	Quintana Roo	MEXICO	58
Lutzomyia	*shannoni*	Altos de Sevilla	-88.6681130443	18.8377203097	Quintana Roo	MEXICO	58
Lutzomyia	*cruciata*	Altos de Sevilla	-88.6671014670	18.8409818897	Quintana Roo	MEXICO	58
Lutzomyia	*cruciata*	Altos de Sevilla	-88.6681130443	18.8377203097	Quintana Roo	MEXICO	58
Lutzomyia	*vexator*	Alturas	-120.5538972222	41.4870805556	California	USA	8, 31
Lutzomyia	*cruciata*	Anastasia State Park	-81.2823250000	29.8778583333	Florida	USA	104, 105
Lutzomyia	*cayennensis*	Andres Quintana Roo	-86.9066666667	21.1322222222	Quintana Roo	MEXICO	18

Lutzomyia	*deleoni*	Andres Quintana Roo	-86.9066666667	21.1322222222	Quintana Roo	MEXICO	18
Lutzomyia	*shannoni*	Andres Quintana Roo	-86.9066666667	21.1322222222	Quintana Roo	MEXICO	18
Lutzomyia	*trinidadensis*	Andres Quintana Roo	-86.9066666667	21.1322222222	Quintana Roo	MEXICO	18
Lutzomyia	*olmeca*	Andres Quintana Roo	-86.9066666667	21.1322222222	Quintana Roo	MEXICO	18
Lutzomyia	*panamensis*	Andres Quintana Roo	-86.9066666667	21.1322222222	Quintana Roo	MEXICO	18
Lutzomyia	*cruciata*	Andres Quintana Roo	-86.9066666667	21.1322222222	Quintana Roo	MEXICO	18
Lutzomyia	*shannoni*	Annie Pond	-87.6773027778	34.7998111111	Alabama	USA	88
Lutzomyia	*vexator*	Ansley	-92.6929277778	32.3956694444	Louisiana	USA	42
Lutzomyia	*shannoni*	Ansley	-92.6929277778	32.3956694444	Louisiana	USA	87
Lutzomyia	*chiapanensis*	Apazapan	-96.7600361111	19.3323972222	Veracruz Ignacio de la Llave	MEXICO	51
Lutzomyia	*longipalpis*	Apazapan	-96.7600361111	19.3323972222	Veracruz Ignacio de la Llave	MEXICO	51
Lutzomyia	*texana*	Arkansas	-96.9641000000	28.0763361111	Texas	USA	104, 105
Lutzomyia	*anthophora*	Arivaca Crek	-111.3317000000	31.5744472222	Arizona	USA	60, 63

Lutzomyia	*californica*	Arivaca Crek	-111.3317000000	31.5744472222	Arizona	USA	60
Lutzomyia	*chiapanensis*	Arroyo Agrio	-96.4308330000	19.7055560000	Veracruz Ignacio de la Llave	MEXICO	51
Lutzomyia	*texana*	Arroyo Agrio	-96.4308330000	19.7055560000	Veracruz Ignacio de la Llave	MEXICO	51
Lutzomyia	*cruciata*	Arroyo Agrio	-96.4308330000	19.7055560000	Veracruz Ignacio de la Llave	MEXICO	51
Lutzomyia	*panamensis*	Arroyo Expangale	-92.9533250000	17.5505250000	Tabasco	MEXICO	40
Lutzomyia	*cruciata*	Arroyo Expangale	-92.9533250000	17.5505250000	Tabasco	MEXICO	105
Brumptomyia	*mesai*	Arroyo Negro	-89.2472220000	17.8597220000	Campeche	MEXICO	75
Lutzomyia	*carpenteri*	Arroyo Negro	-89.2472220000	17.8597220000	Campeche	MEXICO	75
Lutzomyia	*deleoni*	Arroyo Negro	-89.2472220000	17.8597220000	Campeche	MEXICO	75
Lutzomyia	*longipalpis*	Arroyo Negro	-89.2472220000	17.8597220000	Campeche	MEXICO	75
Lutzomyia	*olmeca*	Arroyo Negro	-89.2472220000	17.8597220000	Campeche	MEXICO	75
Lutzomyia	*panamensis*	Arroyo Negro	-89.2472220000	17.8597220000	Campeche	MEXICO	75
Lutzomyia	*shannoni*	Arroyo Negro	-89.2472220000	17.8597220000	Campeche	MEXICO	75

Lutzomyia	*cruciata*	Arroyo Negro	-89.2472220000	17.8597220000	Campeche	MEXICO	75
Lutzomyia	*cruciata*	Asunción Lachixonase	-95.8250000000	16.0952700000	Oaxaca	MEXICO	38
Lutzomyia	*shannoni*	Asuncion Lachixonase (Rio Hondo)	-95.8250000000	16.4286111111	Oaxaca	MEXICO	38
Lutzomyia	*ylephiletor*	Augustine	-88.9833333333	16.9666666667	Distrito el Cayo	BELIZE	53, 101
Lutzomyia	*trinidadensis*	Augustine	-88.9833333333	16.9666666667	Distrito el Cayo	BELIZE	53, 99, 100, 101
Lutzomyia	*shannoni*	Augustine	-88.9833333333	16.9666666667	Distrito el Cayo	BELIZE	53, 101
Lutzomyia	*steatopyga*	Augustine	-88.9833333333	16.9666666667	Distrito el Cayo	BELIZE	99, 100
Lutzomyia	*beltrani*	Augustine	-88.9833333333	16.9666666667	Distrito el Cayo	BELIZE	98
Lutzomyia	*deleoni*	Augustine	-88.9833333333	16.9666666667	Distrito el Cayo	BELIZE	101, 102
Lutzomyia	*olmeca*	Augustine	-88.9833333333	16.9666666667	Distrito el Cayo	BELIZE	101, 102
Lutzomyia	*panamensis*	Augustine	-88.9833333333	16.9666666667	Distrito el Cayo	BELIZE	101, 102
Lutzomyia	*permira*	Augustine	-88.9833333333	16.9666666667	Distrito el Cayo	BELIZE	101, 102
Brumptomyia	*mesai*	Augustine	-88.9833333333	16.9666666667	Distrito el Cayo	BELIZE	57, 99

Brumptomyia	*hamata*	Augustine	-88.9833333333	16.9666666667	Distrito el Cayo	BELIZE	57, 99
Lutzomyia	*cayennensis*	Augustine	-88.9833333333	16.9666666667	Distrito el Cayo	BELIZE	57, 99
Lutzomyia	*cruciata*	Augustine	-88.9833333333	16.9666666667	Distrito el Cayo	BELIZE	101, 102
Lutzomyia	*diabolica*	Austin	-97.7430611111	30.2665333333	Texas	USA	104, 105
Lutzomyia	*texana*	Autlan de Navarro	-104.3660722222	19.7670972222	Jalisco	MEXICO	38
Lutzomyia	*californica*	Bakersfield	-119.0184722222	35.3710972222	California	USA	8
Lutzomyia	*stewarti*	Bakersfield	-119.0184722222	35.3710972222	California	USA	8
Lutzomyia	*vexator*	Bakersfield	-119.0184722222	35.3710972222	California	USA	8
Lutzomyia	*shannoni*	Baking Pot	-89.0194444000	17.2030556000	Distrito el Cayo	BELIZE	99, 100
Brumptomyia	*mesai*	Baking Pot	-89.0194444000	17.2030556000	Distrito el Cayo	BELIZE	57, 99
Lutzomyia	*permira*	Baking Pot	-89.0194444000	17.2030556000	Distrito el Cayo	BELIZE	57, 99
Lutzomyia	*cayennensis*	Baking Pot	-89.0194444000	17.2030556000	Distrito el Cayo	BELIZE	57, 99
Lutzomyia	*trinidadensis*	Baking Pot	-89.0194444000	17.2030556000	Distrito el Cayo	BELIZE	57, 99

Lutzomyia	*deleoni*	Baking Pot	-89.0194444000	17.2030556000	Distrito el Cayo	BELIZE	57, 99
Lutzomyia	*olmeca*	Baking Pot	-89.0194444000	17.2030556000	Distrito el Cayo	BELIZE	57, 99
Lutzomyia	*ovallesi*	Baking Pot	-89.0194444000	17.2030556000	Distrito el Cayo	BELIZE	57, 99
Lutzomyia	*carpenteri*	Baking Pot	-89.0194444000	17.2030556000	Distrito el Cayo	BELIZE	99, 100
Lutzomyia	*cruciata*	Baking Pot	-89.0194444000	17.2030556000	Distrito el Cayo	BELIZE	57, 99
Lutzomyia	*diabolica*	Barksdale	-100.0339611111	29.7251333333	Texas	USA	104, 105
Lutzomyia	*texana*	Barksdale	-100.0339611111	29.7251333333	Texas	USA	104, 105
Lutzomyia	*shannoni*	Baton Rouge	-91.1403194444	30.4577694444	Louisiana	USA	34
Lutzomyia	*vexator*	Baton Rouge	-91.1403194444	30.4577694444	Louisiana	USA	34
Lutzomyia	*shannoni*	Bay Saint Louis	-89.3333222222	30.3053250000	Mississippi	USA	105
Lutzomyia	*shannoni*	Belmopan	-88.7679444444	17.2514166667	Distrito Belice	BELIZE	57, 99
Lutzomyia	*cruciata*	Belmopan	-88.7679444444	20.0000000000	Distrito Belice	BELIZE	57, 99
Brumptomyia	*hamata*	Bencanche	-89.2169444444	19.8741666667	Yucatan	MEXICO	64, 84

Lutzomyia	*carpenteri*	Bencanche	-89.2169444444	19.8741666667	Yucatan	MEXICO	64, 84
Lutzomyia	*deleoni*	Bencanche	-89.2169444444	19.8741666667	Yucatan	MEXICO	64, 84
Lutzomyia	*olmeca*	Bencanche	-89.2169444444	19.8741666667	Yucatan	MEXICO	64, 84
Lutzomyia	*shannoni*	Bencanche	-89.2169444444	19.8741666667	Yucatan	MEXICO	64, 84
Brumptomyia	*mesai*	Bencanche	-89.2169444444	19.8741666667	Yucatan	MEXICO	64, 84
Lutzomyia	*cruciata*	Bencanche	-89.2169444444	19.8741666667	Yucatan	MEXICO	64, 84
Lutzomyia	*trinidadensis*	Big Falls	-88.5833333000	17.4833333000	Distrito el Cayo	BELIZE	57, 99
Lutzomyia	*olmeca*	Big Falls	-88.5833333000	17.4833333000	Distrito el Cayo	BELIZE	57, 99
Lutzomyia	*shannoni*	Big Falls	-88.5833333000	17.4833333000	Distrito el Cayo	BELIZE	57, 99
Lutzomyia	*cruciata*	Big Falls	-88.5833333000	17.4833333000	Distrito el Cayo	BELIZE	57, 99
Lutzomyia	*californica*	Big Pine Key	-80.8700000000	25.4350000000	Florida	USA	8
Lutzomyia	*shannoni*	Billy's Island	-82.3404027778	30.8050694444	Georgia	USA	105
Lutzomyia	*cruciata*	Billy's Island	-82.3404027778	30.8050694444	Georgia	USA	105

Lutzomyia	*trinidadensis*	Blue Creek (San Antonio)	-89.0430194444	19.1954805556	Distrito Toledo	BELIZE	55
Brumptomyia	*mesai*	Blue Creek (San Antonio)	-89.0430194444	19.1954805556	Distrito Toledo	BELIZE	46
Lutzomyia	*ovallesi*	Blue Creek (San Antonio)	-89.0430194444	19.1954805556	Distrito Toledo	BELIZE	46
Lutzomyia	*serrana*	Blue Creek (San Antonio)	-89.0430194444	19.1954805556	Distrito Toledo	BELIZE	46
Lutzomyia	*deleoni*	Blue Creek (San Antonio)	-89.0430194444	19.1954805556	Distrito Toledo	BELIZE	46
Lutzomyia	*shannoni*	Blue Creek (San Antonio)	-89.0430194444	19.1954805556	Distrito Toledo	BELIZE	45, 46, 57
Lutzomyia	*carpenteri*	Blue Creek (San Antonio)	-89.0430194444	19.1954805556	Distrito Toledo	BELIZE	46
Lutzomyia	*olmeca*	Blue Creek (San Antonio)	-89.0430194444	19.1954805556	Distrito Toledo	BELIZE	46
Lutzomyia	*ylephiletor*	Blue Creek (San Antonio)	-89.0430194444	19.1954805556	Distrito Toledo	BELIZE	46
Lutzomyia	*bispinosa*	Blue Creek (San Antonio)	-89.0430194444	19.1954805556	Distrito Toledo	BELIZE	46
Lutzomyia	*cayennensis*	Blue Creek (San Antonio)	-89.0430194444	19.1954805556	Distrito Toledo	BELIZE	46
Lutzomyia	*cruciata*	Blue Creek (San Antonio)	-89.0430194444	19.1954805556	Distrito Toledo	BELIZE	45, 46, 57
Lutzomyia	*diabolica*	Brackettville	-100.4178555556	29.3104861111	Texas	USA	26

Lutzomyia	*texana*	Brackettville	-100.4178555556	29.3104861111	Texas	USA	26
Lutzomyia	*anthophora*	Brackettville	-100.4178555556	29.3104861111	Texas	USA	26
Lutzomyia	*trinidadensis*	Branch Mouth	-89.0800000000	17.1802778000	Distrito el Cayo	BELIZE	53
Lutzomyia	*permira*	Branch Mouth	-89.0800000000	17.1802778000	Distrito el Cayo	BELIZE	53
Lutzomyia	*shannoni*	Branch Mouth	-89.0800000000	17.1802778000	Distrito el Cayo	BELIZE	53
Lutzomyia	*cayennensis*	Branch Mouth	-89.0800000000	17.1802778000	Distrito el Cayo	BELIZE	53, 57, 99
Lutzomyia	*anthophora*	Brooks Air Base	-98.4426638889	29.3411805556	Texas	USA	59, 78
Lutzomyia	*anthophora*	Brownsville	-97.4974833333	25.9016083333	Texas	USA	27, 104, 105
Lutzomyia	*shannoni*	Brownsville	-82.3878722222	28.5527222222	Florida	USA	104, 105
Lutzomyia	*texana*	Brownsville	-97.4974833333	25.9016083333	Texas	USA	25, 104, 105
Lutzomyia	*cruciata*	Brownsville	-97.4974833333	25.9016083333	Texas	USA	26
Lutzomyia	*shannoni*	Buenavista	-94.9313888889	18.2300000000	Veracruz Ignacio de la Llave	MEXICO	51
Lutzomyia	*shannoni*	Buenavista	-94.3583333333	17.7980555555	Veracruz Ignacio de la Llave	MEXICO	51

Lutzomyia	*cruciata*	Buenavista	-94.8466666667	17.5413888880	Veracruz Ignacio de la Llave	MEXICO	38
Lutzomyia	*dodgei*	Buenavista	-99.4076166667	18.4589194444	Guerrero	MEXICO	46
Lutzomyia	*anthophora*	Buenos Aires National Wildlife Refuge	-111.5421916667	31.4886055556	Arizona	USA	63
Lutzomyia	*panamensis*	Bullet Tre Falls	-89.1127778000	17.1722222000	Distrito el Cayo	BELIZE	57, 99
Lutzomyia	*olmeca*	Bullet Tre Falls	-89.1127778000	17.1722222000	Distrito el Cayo	BELIZE	57, 99
Lutzomyia	*shannoni*	Bullet Tre Falls	-89.1127778000	17.1722222000	Distrito el Cayo	BELIZE	57, 99
Lutzomyia	*carpenteri*	Bullet Tre Falls	-89.1127778000	17.1722222000	Distrito el Cayo	BELIZE	99, 100
Lutzomyia	*cruciata*	Bullet Tre Falls	-89.1127778000	17.1722222000	Distrito el Cayo	BELIZE	57, 99
Lutzomyia	*californica*	Buttonwillow	-119.4695611111	35.4004694444	California	USA	8
Lutzomyia	*stewarti*	Byron	-121.6380222222	37.8671361111	California	USA	8
Lutzomyia	*vexator*	Byron	-121.6380222222	37.8671361111	California	USA	8
Lutzomyia	*cruciata*	Cabañas	-92.2450000000	15.3641666667	Chiapas	MEXICO	43
Lutzomyia	*cayennensis*	Cafetal Ojo de Agua	-105.1416666667	21.9519027778	Nayarit	MEXICO	38

Lutzomyia	*californica*	Cahuilla Hills	-116.4164000000	33.6887222222	California	USA	105
Lutzomyia	*stewarti*	Calaveras Creek	-121.8477527778	37.5205250000	California	USA	8, 56
Lutzomyia	*diabolica*	Calderas de Cofrados	-104.7147200000	21.6230600000	Nayarit	MEXICO	40
Lutzomyia	*shannoni*	Calderas de Cofrados	-104.7147200000	21.6230600000	Nayarit	MEXICO	40
Lutzomyia	*cruciata*	Calderas de Cofrados	-104.7147200000	21.6230600000	Nayarit	MEXICO	40
Lutzomyia	*stewarti*	Caliente Creek	-118.8339861111	35.2796944444	California	USA	8
Lutzomyia	*cruciata*	Camalote	-96.6852777778	15.9777777778	Oaxaca	MEXICO	38
Lutzomyia	*diabolica*	Camp Bullis	-98.5501277778	29.6747027778	Texas	USA	104, 105
Lutzomyia	*cruciata*	Campestre Las Bajadas	-96.1944444444	19.1505555556	Veracruz Ignacio de la Llave	MEXICO	38
Lutzomyia	*deleoni*	Canchacan	-89.4188888889	16.2733333333	Peten	GUATEMALA	45, 46, 57
Lutzomyia	*shannoni*	Caney Lake	-93.6090388889	30.7157000000	Louisiana	USA	87
Lutzomyia	*diabolica*	Canyon Lake	-98.2622722222	29.8753222222	Texas	USA	104, 105
Lutzomyia	*diabolica*	Cañada del Zapote	-97.1272222222	18.2352777778	Puebla	MEXICO	38

Lutzomyia	*diabolica*	Cañon de los Lobos	-99.1166944443	18.8764722216	Morelos	MEXICO	32
Lutzomyia	*stewarti*	Cañon Guadalupe	-115.4453416667	32.6455583333	Baja California Norte	MEXICO	48
Lutzomyia	*deleoni*	Caobas	-89.1104835807	18.3843231325	Quintana Roo	MEXICO	58
Lutzomyia	*olmeca*	Caobas	-89.1104835807	18.3843231325	Quintana Roo	MEXICO	58
Lutzomyia	*ovallesi*	Caobas	-89.1104835807	18.3843231325	Quintana Roo	MEXICO	58
Lutzomyia	*panamensis*	Caobas	-89.1104835807	18.3843231325	Quintana Roo	MEXICO	58
Lutzomyia	*shannoni*	Caobas	-89.1104835807	18.3843231325	Quintana Roo	MEXICO	58
Lutzomyia	*steatopyga*	Caobas	-89.1104835807	18.3843231325	Quintana Roo	MEXICO	58
Lutzomyia	*ovallesi*	Caobas	-89.1116224669	18.3846085913	Quintana Roo	MEXICO	58
Lutzomyia	*steatopyga*	Caobas	-89.1116224669	18.3846085913	Quintana Roo	MEXICO	58
Lutzomyia	*deleoni*	Caobas	-89.1116224669	18.3846085913	Quintana Roo	MEXICO	58
Lutzomyia	*olmeca*	Caobas	-89.1116224669	18.3846085913	Quintana Roo	MEXICO	58
Lutzomyia	*shannoni*	Caobas	-89.1116224669	18.3846085913	Quintana Roo	MEXICO	58

Lutzomyia	*cruciata*	Caobas	-89.1104835807	18.3843231325	Quintana Roo	MEXICO	58
Lutzomyia	*cruciata*	Caobas	-89.1116224669	18.3846085913	Quintana Roo	MEXICO	58
Lutzomyia	*stewarti*	Capay	-122.0828388889	39.7937611111	California	USA	8
Lutzomyia	*vexator*	Capay	-122.0828388889	39.7937611111	California	USA	8
Lutzomyia	*californica*	Capay	-122.0828388889	39.7937611111	California	USA	8
Lutzomyia	*cruciata*	Caracoles Estrella	-96.4283333333	17.9469444444	Oaxaca	MEXICO	40
Lutzomyia	*longipalpis*	Caracuaro de Morelos	-101.1203861111	19.0163666667	Michoacan de Ocampo	MEXICO	43
Lutzomyia	*olmeca*	Cardenas	-93.3763888889	18.0001111111	Tabasco	MEXICO	40
Lutzomyia	*texana*	Cascada El Salto	-99.3301388882	22.5223999933	San Luis Potosi	MEXICO	105
Lutzomyia	*panamensis*	Caves Branch	-88.7000000000	17.1333333000	Distrito el Cayo	BELIZE	57, 99
Lutzomyia	*trinidadensis*	Caves Branch	-88.7000000000	17.1333333000	Distrito el Cayo	BELIZE	57, 99
Lutzomyia	*shannoni*	Caves Branch	-88.7000000000	17.1333333000	Distrito el Cayo	BELIZE	57, 99
Lutzomyia	*ovallesi*	Caves Branch	-88.7000000000	17.1333333000	Distrito el Cayo	BELIZE	57, 99

Lutzomyia	*cruciata*	Caves Branch	-88.7000000000	17.1333333000	Distrito el Cayo	BELIZE	57, 99
Lutzomyia	*anthophora*	Cenobio Moreno	-102.5041666667	19.0955555556	Michoacan de Ocampo	MEXICO	38
Lutzomyia	*cayennensis*	Cenobio Moreno	-102.5041666667	19.0955555556	Michoacan de Ocampo	MEXICO	38
Lutzomyia	*cruciata*	Cenote Azul	-87.3022200000	20.4877800000	Quintana Roo	MEXICO	105
Lutzomyia	*permira*	Central Farm	-89.0000000000	17.3186555556	Distrito el Cayo	BELIZE	99, 100
Lutzomyia	*olmeca*	Central Farm	-89.0000000000	17.3186555556	Distrito el Cayo	BELIZE	99, 100
Brumptomyia	*mesai*	Central Farm	-89.0000000000	17.3186555556	Distrito el Cayo	BELIZE	57
Brumptomyia	*hamata*	Central Farm	-89.0000000000	17.3186555556	Distrito el Cayo	BELIZE	57
Lutzomyia	*panamensis*	Central Farm	-89.0000000000	17.3186555556	Distrito el Cayo	BELIZE	57
Lutzomyia	*cayennensis*	Central Farm	-89.0000000000	17.3186555556	Distrito el Cayo	BELIZE	57
Lutzomyia	*trinidadensis*	Central Farm	-89.0000000000	17.3186555556	Distrito el Cayo	BELIZE	57
Lutzomyia	*beltrani*	Central Farm	-89.0000000000	17.3186555556	Distrito el Cayo	BELIZE	57
Lutzomyia	*deleoni*	Central Farm	-89.0000000000	17.3186555556	Distrito el Cayo	BELIZE	57

Lutzomyia	*shannoni*	Central Farm	-89.0000000000	17.3186555556	Distrito el Cayo	BELIZE	57
Lutzomyia	*ovallesi*	Central Farm	-89.0000000000	17.3186555556	Distrito el Cayo	BELIZE	57
Lutzomyia	*carpenteri*	Central Farm	-89.0000000000	17.3186555556	Distrito el Cayo	BELIZE	99, 100
Lutzomyia	*cruciata*	Central Farm	-89.0000000000	17.3186555556	Distrito el Cayo	BELIZE	99, 100
Lutzomyia	*carpenteri*	Central Vallarta	-87.0661297625	20.8638944689	Quintana Roo	MEXICO	58
Lutzomyia	*cayennensis*	Central Vallarta	-87.0661297625	20.8638944689	Quintana Roo	MEXICO	58
Lutzomyia	*deleoni*	Central Vallarta	-87.0661297625	20.8638944689	Quintana Roo	MEXICO	58
Lutzomyia	*olmeca*	Central Vallarta	-87.0661297625	20.8638944689	Quintana Roo	MEXICO	58
Lutzomyia	*shannoni*	Central Vallarta	-87.0661297625	20.8638944689	Quintana Roo	MEXICO	58
Lutzomyia	*steatopyga*	Central Vallarta	-87.0661297625	20.8638944689	Quintana Roo	MEXICO	58
Lutzomyia	*trinidadensis*	Central Vallarta	-87.0661297625	20.8638944689	Quintana Roo	MEXICO	58
Brumptomyia	*mesai*	Central Vallarta	-87.0661297625	20.8638944689	Quintana Roo	MEXICO	58
Lutzomyia	*undulata*	Central Vallarta	-87.0661297625	20.8638944689	Quintana Roo	MEXICO	58

Brumptomyia	*mesai*	Central Vallarta	-87.0783913080	20.8727985729	Quintana Roo	MEXICO	58
Lutzomyia	*steatopyga*	Central Vallarta	-87.0783913080	20.8727985729	Quintana Roo	MEXICO	58
Lutzomyia	*undulata*	Central Vallarta	-87.0783913080	20.8727985729	Quintana Roo	MEXICO	58
Lutzomyia	*carpenteri*	Central Vallarta	-87.0783913080	20.8727985729	Quintana Roo	MEXICO	58
Lutzomyia	*cayennensis*	Central Vallarta	-87.0783913080	20.8727985729	Quintana Roo	MEXICO	58
Lutzomyia	*olmeca*	Central Vallarta	-87.0783913080	20.8727985729	Quintana Roo	MEXICO	58
Lutzomyia	*shannoni*	Central Vallarta	-87.0783913080	20.8727985729	Quintana Roo	MEXICO	58
Lutzomyia	*cruciata*	Central Vallarta	-87.0783913080	20.8727985729	Quintana Roo	MEXICO	58
Lutzomyia	*diabolica*	Chaparral Wildlife Management Area	-99.8124944444	28.4116500000	Texas	USA	59
Brumptomyia	*mesai*	Chavarillo	-96.7913890000	19.4252780000	Veracruz Ignacio de la Llave	MEXICO	51
Lutzomyia	*longipalpis*	Chavarillo	-96.7913890000	19.4252780000	Veracruz Ignacio de la Llave	MEXICO	51
Lutzomyia	*texana*	Chavarillo	-96.7913890000	19.4252780000	Veracruz Ignacio de la Llave	MEXICO	51
Lutzomyia	*cruciata*	Chavarillo	-96.7913890000	19.4252780000	Veracruz Ignacio de la Llave	MEXICO	51

Lutzomyia	*shannoni*	Chemin-A-Haut State Park	-91.8491888889	32.9083861111	Louisiana	USA	87
Lutzomyia	*cruciata*	Chetumal	-88.3034500000	18.5025861111	Quintana Roo	MEXICO	43
Brumptomyia	*mesai*	Chetumal - N1	-88.3278888889	18.5340305556	Quintana Roo	MEXICO	89, 90
Lutzomyia	*deleoni*	Chetumal - N1	-88.3278888889	18.5340305556	Quintana Roo	MEXICO	89, 90
Lutzomyia	*olmeca*	Chetumal - N1	-88.3278888889	18.5340305556	Quintana Roo	MEXICO	89, 90
Lutzomyia	*shannoni*	Chetumal - N1	-88.3278888889	18.5340305556	Quintana Roo	MEXICO	89, 90
Lutzomyia	*cruciata*	Chetumal - N1	-88.3278888889	18.5340305556	Quintana Roo	MEXICO	89, 90
Lutzomyia	*deleoni*	Chetumal - N10	-88.2983138856	18.5462638856	Quintana Roo	MEXICO	89, 90
Brumptomyia	*mesai*	Chetumal - N10	-88.2983138856	18.5462638856	Quintana Roo	MEXICO	89, 90
Lutzomyia	*olmeca*	Chetumal - N10	-88.2983138856	18.5462638856	Quintana Roo	MEXICO	89, 90
Lutzomyia	*shannoni*	Chetumal - N10	-88.2983138856	18.5462638856	Quintana Roo	MEXICO	89, 90
Lutzomyia	*steatopyga*	Chetumal - N10	-88.2983138856	18.5462638856	Quintana Roo	MEXICO	89, 90
Lutzomyia	*cruciata*	Chetumal - N10	-88.2983138856	18.5462638856	Quintana Roo	MEXICO	89, 90

Lutzomyia	*deleoni*	Chetumal - N11	-88.2556277778	18.5933333327	Quintana Roo	MEXICO	89, 90
Brumptomyia	*mesai*	Chetumal - N11	-88.2556277778	18.5933333327	Quintana Roo	MEXICO	89, 90
Lutzomyia	*steatopyga*	Chetumal - N11	-88.2556277778	18.5933333327	Quintana Roo	MEXICO	89, 90
Lutzomyia	*olmeca*	Chetumal - N11	-88.2556277778	18.5933333327	Quintana Roo	MEXICO	89, 90
Lutzomyia	*shannoni*	Chetumal - N11	-88.2556277778	18.5933333327	Quintana Roo	MEXICO	89, 90
Lutzomyia	*cruciata*	Chetumal - N11	-88.2556277778	18.5933333327	Quintana Roo	MEXICO	89, 90
Brumptomyia	*mesai*	Chetumal - N12	-88.3889577778	18.5448250000	Quintana Roo	MEXICO	89, 90
Lutzomyia	*olmeca*	Chetumal - N12	-88.3889577778	18.5448250000	Quintana Roo	MEXICO	89, 90
Lutzomyia	*shannoni*	Chetumal - N12	-88.3889577778	18.5448250000	Quintana Roo	MEXICO	89, 90
Lutzomyia	*carpenteri*	Chetumal - N12	-88.3889577778	18.5448250000	Quintana Roo	MEXICO	89, 90
Lutzomyia	*deleoni*	Chetumal - N12	-88.3889577778	18.5448250000	Quintana Roo	MEXICO	89, 90
Brumptomyia	*hamata*	Chetumal - N12	-88.3889577778	18.5448250000	Quintana Roo	MEXICO	89, 90
Lutzomyia	*cruciata*	Chetumal - N12	-88.3889577778	18.5448250000	Quintana Roo	MEXICO	89, 90

Brumptomyia	*mesai*	Chetumal - N2	-88.3227888889	18.5464416667	Quintana Roo	MEXICO	89, 90
Lutzomyia	*ovallesi*	Chetumal - N2	-88.3227888889	18.5464416667	Quintana Roo	MEXICO	89, 90
Lutzomyia	*deleoni*	Chetumal - N2	-88.3227888889	18.5464416667	Quintana Roo	MEXICO	89, 90
Lutzomyia	*olmeca*	Chetumal - N2	-88.3227888889	18.5464416667	Quintana Roo	MEXICO	89, 90
Lutzomyia	*shannoni*	Chetumal - N2	-88.3227888889	18.5464416667	Quintana Roo	MEXICO	89, 90
Lutzomyia	*cruciata*	Chetumal - N2	-88.3227888889	18.5464416667	Quintana Roo	MEXICO	89, 90
Brumptomyia	*mesai*	Chetumal - N3	-88.3237888889	18.5477500000	Quintana Roo	MEXICO	89, 90
Lutzomyia	*ovallesi*	Chetumal - N3	-88.3237888889	18.5477500000	Quintana Roo	MEXICO	89, 90
Lutzomyia	*deleoni*	Chetumal - N3	-88.3237888889	18.5477500000	Quintana Roo	MEXICO	89, 90
Lutzomyia	*olmeca*	Chetumal - N3	-88.3237888889	18.5477500000	Quintana Roo	MEXICO	89, 90
Lutzomyia	*shannoni*	Chetumal - N3	-88.3237888889	18.5477500000	Quintana Roo	MEXICO	89, 90
Lutzomyia	*cruciata*	Chetumal - N3	-88.3237888889	18.5477500000	Quintana Roo	MEXICO	89, 90
Brumptomyia	*mesai*	Chetumal - N4	-88.3160583333	18.5647777778	Quintana Roo	MEXICO	89, 90

Lutzomyia	*carpenteri*	Chetumal - N4	-88.3160583333	18.5647777778	Quintana Roo	MEXICO	89, 90
Lutzomyia	*ovallesi*	Chetumal - N4	-88.3160583333	18.5647777778	Quintana Roo	MEXICO	89, 90
Lutzomyia	*deleoni*	Chetumal - N4	-88.3160583333	18.5647777778	Quintana Roo	MEXICO	89, 90
Lutzomyia	*olmeca*	Chetumal - N4	-88.3160583333	18.5647777778	Quintana Roo	MEXICO	89, 90
Lutzomyia	*shannoni*	Chetumal - N4	-88.3160583333	18.5647777778	Quintana Roo	MEXICO	89, 90
Lutzomyia	*cruciata*	Chetumal - N4	-88.3160583333	18.5647777778	Quintana Roo	MEXICO	89, 90
Brumptomyia	*mesai*	Chetumal - N5	-88.3215805556	18.5400555556	Quintana Roo	MEXICO	89, 90
Lutzomyia	*ovallesi*	Chetumal - N5	-88.3215805556	18.5400555556	Quintana Roo	MEXICO	89, 90
Lutzomyia	*deleoni*	Chetumal - N5	-88.3215805556	18.5400555556	Quintana Roo	MEXICO	89, 90
Lutzomyia	*olmeca*	Chetumal - N5	-88.3215805556	18.5400555556	Quintana Roo	MEXICO	89, 90
Lutzomyia	*shannoni*	Chetumal - N5	-88.3215805556	18.5400555556	Quintana Roo	MEXICO	89, 90
Lutzomyia	*cruciata*	Chetumal - N5	-88.3215805556	18.5400555556	Quintana Roo	MEXICO	89, 90
Brumptomyia	*mesai*	Chetumal - N6	-88.3967361111	18.5696777778	Quintana Roo	MEXICO	89, 90

Lutzomyia	*ovallesi*	Chetumal - N6	-88.3967361111	18.5696777778	Quintana Roo	MEXICO	89, 90
Lutzomyia	*deleoni*	Chetumal - N6	-88.3967361111	18.5696777778	Quintana Roo	MEXICO	89, 90
Lutzomyia	*olmeca*	Chetumal - N6	-88.3967361111	18.5696777778	Quintana Roo	MEXICO	89, 90
Lutzomyia	*shannoni*	Chetumal - N6	-88.3967361111	18.5696777778	Quintana Roo	MEXICO	89, 90
Lutzomyia	*cruciata*	Chetumal - N6	-88.3967361111	18.5696777778	Quintana Roo	MEXICO	89, 90
Lutzomyia	*deleoni*	Chetumal - N7	-88.3216972222	18.5479750000	Quintana Roo	MEXICO	89, 90
Lutzomyia	*olmeca*	Chetumal - N7	-88.3216972222	18.5479750000	Quintana Roo	MEXICO	89, 90
Lutzomyia	*shannoni*	Chetumal - N7	-88.3216972222	18.5479750000	Quintana Roo	MEXICO	89, 90
Lutzomyia	*cruciata*	Chetumal - N7	-88.3216972222	18.5479750000	Quintana Roo	MEXICO	89, 90
Lutzomyia	*deleoni*	Chetumal - N8	-88.2675416667	18.5356055222	Quintana Roo	MEXICO	89, 90
Lutzomyia	*ovallesi*	Chetumal - N8	-88.2675416667	18.5356055222	Quintana Roo	MEXICO	89, 90
Lutzomyia	*cruciata*	Chetumal - N8	-88.2675416667	18.5356055222	Quintana Roo	MEXICO	89, 90
Brumptomyia	*mesai*	Chetumal - N9	-88.2686332660	18.5742333267	Quintana Roo	MEXICO	89, 90

Lutzomyia	*shannoni*	Chetumal - N9	-88.2686332660	18.5742333267	Quintana Roo	MEXICO	89, 90
Lutzomyia	*deleoni*	Chetumal - N9	-88.2686332660	18.5742333267	Quintana Roo	MEXICO	89, 90
Lutzomyia	*olmeca*	Chetumal - N9	-88.2686332660	18.5742333267	Quintana Roo	MEXICO	89, 90
Lutzomyia	*cruciata*	Chetumal - N9	-88.2686332660	18.5742333267	Quintana Roo	MEXICO	89, 90
Brumptomyia	*mesai*	Chetumal - S1	-88.3228888889	18.5064861111	Quintana Roo	MEXICO	89, 90
Lutzomyia	*ovallesi*	Chetumal - S1	-88.3228888889	18.5064861111	Quintana Roo	MEXICO	89, 90
Lutzomyia	*deleoni*	Chetumal - S1	-88.3228888889	18.5064861111	Quintana Roo	MEXICO	89, 90
Lutzomyia	*olmeca*	Chetumal - S1	-88.3228888889	18.5064861111	Quintana Roo	MEXICO	89, 90
Lutzomyia	*shannoni*	Chetumal - S1	-88.3228888889	18.5064861111	Quintana Roo	MEXICO	89, 90
Lutzomyia	*cruciata*	Chetumal - S1	-88.3228888889	18.5064861111	Quintana Roo	MEXICO	89, 90
Brumptomyia	*mesai*	Chetumal - S2	-88.3227888889	18.5071388889	Quintana Roo	MEXICO	89, 90
Lutzomyia	*cayennensis*	Chetumal - S2	-88.3227888889	18.5071388889	Quintana Roo	MEXICO	89, 90
Lutzomyia	*deleoni*	Chetumal - S2	-88.3227888889	18.5071388889	Quintana Roo	MEXICO	89, 90

Lutzomyia	*longipalpis*	Chetumal - S2	-88.3227888889	18.5071388889	Quintana Roo	MEXICO	89, 90
Lutzomyia	*olmeca*	Chetumal - S2	-88.3227888889	18.5071388889	Quintana Roo	MEXICO	89, 90
Lutzomyia	*permira*	Chetumal - S2	-88.3227888889	18.5071388889	Quintana Roo	MEXICO	89, 90
Lutzomyia	*shannoni*	Chetumal - S2	-88.3227888889	18.5071388889	Quintana Roo	MEXICO	89, 90
Lutzomyia	*cruciata*	Chetumal - S2	-88.3227888889	18.5071388889	Quintana Roo	MEXICO	89, 90
Brumptomyia	*mesai*	Chetumal - S3	-88.3227888889	18.5120527778	Quintana Roo	MEXICO	89, 90
Lutzomyia	*ovallesi*	Chetumal - S3	-88.3227888889	18.5120527778	Quintana Roo	MEXICO	89, 90
Lutzomyia	*deleoni*	Chetumal - S3	-88.3227888889	18.5120527778	Quintana Roo	MEXICO	89, 90
Lutzomyia	*olmeca*	Chetumal - S3	-88.3227888889	18.5120527778	Quintana Roo	MEXICO	89, 90
Lutzomyia	*shannoni*	Chetumal - S3	-88.3227888889	18.5120527778	Quintana Roo	MEXICO	89, 90
Lutzomyia	*cruciata*	Chetumal - S3	-88.3227888889	18.5120527778	Quintana Roo	MEXICO	89, 90
Lutzomyia	*deleoni*	Chetumal - S4	-88.3380000000	18.4877416633	Quintana Roo	MEXICO	89, 90
Brumptomyia	*mesai*	Chetumal - S4	-88.3380000000	18.4877416633	Quintana Roo	MEXICO	89, 90

Lutzomyia	*olmeca*	Chetumal - S4	-88.3380000000	18.4877416633	Quintana Roo	MEXICO	89, 90
Lutzomyia	*trinidadensis*	Chetumal - S4	-88.3380000000	18.4877416633	Quintana Roo	MEXICO	89, 90
Lutzomyia	*cruciata*	Chetumal - S4	-88.3380000000	18.4877416633	Quintana Roo	MEXICO	89, 90
Lutzomyia	*deleoni*	Chetumal - S5	-88.3471638889	18.5095305556	Quintana Roo	MEXICO	89, 90
Lutzomyia	*ylephiletor*	Chetumal - S5	-88.3471638889	18.5095305556	Quintana Roo	MEXICO	89, 90
Lutzomyia	*olmeca*	Chetumal - S5	-88.3471638889	18.5095305556	Quintana Roo	MEXICO	89, 90
Lutzomyia	*shannoni*	Chetumal - S5	-88.3471638889	18.5095305556	Quintana Roo	MEXICO	89, 90
Lutzomyia	*cruciata*	Chetumal - S5	-88.3471638889	18.5095305556	Quintana Roo	MEXICO	89, 90
Lutzomyia	*deleoni*	Chetumal - S6	-88.3366166666	18.4975694441	Quintana Roo	MEXICO	89, 90
Lutzomyia	*olmeca*	Chetumal - S6	-88.3366166666	18.4975694441	Quintana Roo	MEXICO	89, 90
Lutzomyia	*cruciata*	Chetumal - S6	-88.3366166666	18.4975694441	Quintana Roo	MEXICO	89, 90
Lutzomyia	*deleoni*	Chetumal - S7	-88.3359750000	18.4880861111	Quintana Roo	MEXICO	89, 90
Lutzomyia	*olmeca*	Chetumal - S7	-88.3359750000	18.4880861111	Quintana Roo	MEXICO	89, 90

Lutzomyia	*cruciata*	Chetumal - S7	-88.3359750000	18.4880861111	Quintana Roo	MEXICO	89, 90
Lutzomyia	*shannoni*	Chewacla State Park	-85.4727250000	32.5511000000	Alabama	USA	105
Lutzomyia	*chiapanensis*	Chiapa de Corzo	-93.0138888889	16.7069444444	Chiapas	MEXICO	28
Lutzomyia	*longipalpis*	Chichen Itza	-88.5686444444	20.6826027778	Yucatan	MEXICO	36
Lutzomyia	*trinidadensis*	Chichen Itza	-88.5686444444	20.6826027778	Yucatan	MEXICO	37
Lutzomyia	*shannoni*	Chiltoyac	-96.8641666667	19.5747222222	Veracruz Ignacio de la Llave	MEXICO	51
Lutzomyia	*texana*	Chiltoyac	-96.8641666667	19.5747222222	Veracruz Ignacio de la Llave	MEXICO	51
Lutzomyia	*cruciata*	Chiltoyac	-96.8641666667	19.5747222222	Veracruz Ignacio de la Llave	MEXICO	51
Lutzomyia	*steatopyga*	Chiquibul	-88.9695694444	17.1691194444	Distrito el Cayo	BELIZE	99, 100
Lutzomyia	*trinidadensis*	Chiquibul	-88.9695694444	17.1691194444	Distrito el Cayo	BELIZE	99, 100
Lutzomyia	*shannoni*	Chiquibul	-88.9695694444	17.1691194444	Distrito el Cayo	BELIZE	99, 100
Lutzomyia	*ovallesi*	Chiquibul	-88.9695694444	17.1691194444	Distrito el Cayo	BELIZE	99, 100
Lutzomyia	*panamensis*	Chiquibul	-88.9695694444	17.1691194444	Distrito el Cayo	BELIZE	99, 100

Lutzomyia	*olmeca*	Chiquibul	-88.9695694444	17.1691194444	Distrito el Cayo	BELIZE	99, 100
Lutzomyia	*deleoni*	Chiquibul	-88.9695694444	17.1691194444	Distrito el Cayo	BELIZE	99, 100
Lutzomyia	*ylephiletor*	Chiquibul	-88.9695694444	17.1691194444	Distrito el Cayo	BELIZE	99, 100
Lutzomyia	*carpenteri*	Chiquibul	-88.9695694444	17.1691194444	Distrito el Cayo	BELIZE	99, 100
Lutzomyia	*permira*	Chiquibul	-88.9695694444	17.1691194444	Distrito el Cayo	BELIZE	99, 100
Brumptomyia	*mesai*	Chiquibul	-88.9695694444	17.1691194444	Distrito el Cayo	BELIZE	57
Lutzomyia	*bispinosa*	Chiquibul	-88.9695694444	17.1691194444	Distrito el Cayo	BELIZE	57
Lutzomyia	*cayennensis*	Chiquibul	-88.9695694444	17.1691194444	Distrito el Cayo	BELIZE	57
Lutzomyia	*beltrani*	Chiquibul	-88.9695694444	17.1691194444	Distrito el Cayo	BELIZE	57
Lutzomyia	*cruciata*	Chiquibul	-88.9695694444	17.1691194444	Distrito el Cayo	BELIZE	99, 100
Lutzomyia	*shannoni*	Christian	-87.4603972222	36.8410583333	Kentucky	USA	67
Lutzomyia	*vexator*	Christian	-87.4603972222	36.8410583333	Kentucky	USA	67
Lutzomyia	*cayennensis*	Chumpom	-87.7661329078	19.9611396269	Quintana Roo	MEXICO	58

Lutzomyia	*deleoni*	Chumpom	-87.7661329078	19.9611396269	Quintana Roo	MEXICO	58
Lutzomyia	*shannoni*	Chumpom	-87.7661329078	19.9611396269	Quintana Roo	MEXICO	58
Lutzomyia	*steatopyga*	Chumpom	-87.7661329078	19.9611396269	Quintana Roo	MEXICO	58
Lutzomyia	*trinidadensis*	Chumpom	-87.7661329078	19.9611396269	Quintana Roo	MEXICO	58
Lutzomyia	*olmeca*	Chumpom	-87.7661329078	19.9611396269	Quintana Roo	MEXICO	58
Lutzomyia	*cratifer*	Chumpom	-87.7661329078	19.9611396269	Quintana Roo	MEXICO	58
Lutzomyia	*undulata*	Chumpom	-87.7661329078	19.9611396269	Quintana Roo	MEXICO	58
Lutzomyia	*cratifer*	Chumpom	-87.7701552139	19.9648541270	Quintana Roo	MEXICO	58
Brumptomyia	*mesai*	Chumpom	-87.7701552139	19.9648541270	Quintana Roo	MEXICO	58
Lutzomyia	*cayennensis*	Chumpom	-87.7701552139	19.9648541270	Quintana Roo	MEXICO	58
Lutzomyia	*olmeca*	Chumpom	-87.7701552139	19.9648541270	Quintana Roo	MEXICO	58
Lutzomyia	*ovallesi*	Chumpom	-87.7701552139	19.9648541270	Quintana Roo	MEXICO	58
Lutzomyia	*serrana*	Chumpom	-87.7701552139	19.9648541270	Quintana Roo	MEXICO	58

Lutzomyia	*shannoni*	Chumpom	-87.7701552139	19.9648541270	Quintana Roo	MEXICO	58
Lutzomyia	*steatopyga*	Chumpom	-87.7701552139	19.9648541270	Quintana Roo	MEXICO	58
Lutzomyia	*undulata*	Chumpom	-87.7701552139	19.9648541270	Quintana Roo	MEXICO	58
Lutzomyia	*cruciata*	Chumpom	-87.7661329078	19.9611396269	Quintana Roo	MEXICO	58
Lutzomyia	*cruciata*	Chumpom	-87.7701552139	19.9648541270	Quintana Roo	MEXICO	58
Lutzomyia	*trinidadensis*	Chum-Yaxche	-87.6144444444	20.0763888889	Quintana Roo	MEXICO	18
Lutzomyia	*cayennensis*	Chum-Yaxche	-87.6144444444	20.0763888889	Quintana Roo	MEXICO	18
Lutzomyia	*shannoni*	Chum-Yaxche	-87.6144444444	20.0763888889	Quintana Roo	MEXICO	18
Lutzomyia	*deleoni*	Chum-Yaxche	-87.6144444444	20.0763888889	Quintana Roo	MEXICO	18
Lutzomyia	*olmeca*	Chum-Yaxche	-87.6144444444	20.0763888889	Quintana Roo	MEXICO	18
Lutzomyia	*panamensis*	Chum-Yaxche	-87.6144444444	20.0763888889	Quintana Roo	MEXICO	18
Lutzomyia	*cruciata*	Chum-Yaxche	-87.6144444444	20.0763888889	Quintana Roo	MEXICO	18
Lutzomyia	*trinidadensis*	Chunhuas	-88.2105555556	19.6319444444	Quintana Roo	MEXICO	18

Lutzomyia	*cayennensis*	Chunhuas	-88.2105555556	19.6319444444	Quintana Roo	MEXICO	18
Lutzomyia	*shannoni*	Chunhuas	-88.2105555556	19.6319444444	Quintana Roo	MEXICO	18
Lutzomyia	*olmeca*	Chunhuas	-88.2105555556	19.6319444444	Quintana Roo	MEXICO	18
Lutzomyia	*panamensis*	Chunhuas	-88.2105555556	19.6319444444	Quintana Roo	MEXICO	18
Lutzomyia	*deleoni*	Chunhuas	-88.2105555556	19.6319444444	Quintana Roo	MEXICO	18
Lutzomyia	*cruciata*	Chunhuas	-88.2105555556	19.6319444444	Quintana Roo	MEXICO	18
Lutzomyia	*deleoni*	Chunhuhub	-88.5536796354	19.5783123783	Quintana Roo	MEXICO	58
Brumptomyia	*mesai*	Chunhuhub	-88.5536796354	19.5783123783	Quintana Roo	MEXICO	58
Brumptomyia	*hamata*	Chunhuhub	-88.5536796354	19.5783123783	Quintana Roo	MEXICO	58
Lutzomyia	*shannoni*	Chunhuhub	-88.5536796354	19.5783123783	Quintana Roo	MEXICO	58
Lutzomyia	*steatopyga*	Chunhuhub	-88.5536796354	19.5783123783	Quintana Roo	MEXICO	58
Lutzomyia	*olmeca*	Chunhuhub	-88.5536796354	19.5783123783	Quintana Roo	MEXICO	58
Lutzomyia	*undulata*	Chunhuhub	-88.5536796354	19.5783123783	Quintana Roo	MEXICO	58

Lutzomyia	*deleoni*	Chunhuhub	-88.5907666667	19.5889444444	Quintana Roo	MEXICO	70
Lutzomyia	*olmeca*	Chunhuhub	-88.5907666667	19.5889444444	Quintana Roo	MEXICO	70
Lutzomyia	*ovallesi*	Chunhuhub	-88.5907666667	19.5889444444	Quintana Roo	MEXICO	70
Lutzomyia	*steatopyga*	Chunhuhub	-88.5907666667	19.5889444444	Quintana Roo	MEXICO	70
Lutzomyia	*shannoni*	Chunhuhub	-88.5907666667	19.5889444444	Quintana Roo	MEXICO	70
Lutzomyia	*carpenteri*	Chunhuhub	-88.5638410151	19.5872409680	Quintana Roo	MEXICO	58
Brumptomyia	*mesai*	Chunhuhub	-88.5638410151	19.5872409680	Quintana Roo	MEXICO	58
Brumptomyia	*hamata*	Chunhuhub	-88.5638410151	19.5872409680	Quintana Roo	MEXICO	58
Lutzomyia	*shannoni*	Chunhuhub	-88.5638410151	19.5872409680	Quintana Roo	MEXICO	58
Lutzomyia	*steatopyga*	Chunhuhub	-88.5638410151	19.5872409680	Quintana Roo	MEXICO	58
Lutzomyia	*deleoni*	Chunhuhub	-88.5638410151	19.5872409680	Quintana Roo	MEXICO	58
Lutzomyia	*olmeca*	Chunhuhub	-88.5638410151	19.5872409680	Quintana Roo	MEXICO	58
Lutzomyia	*trinidadensis*	Chunhuhub	-88.5638410151	19.5872409680	Quintana Roo	MEXICO	58

Lutzomyia	*undulata*	Chunhuhub	-88.5638410151	19.5872409680	Quintana Roo	MEXICO	58
Lutzomyia	*cruciata*	Chunhuhub	-88.5536796354	19.5783123783	Quintana Roo	MEXICO	58
Lutzomyia	*cruciata*	Chunhuhub	-88.5907666667	19.5889444444	Quintana Roo	MEXICO	70
Lutzomyia	*cruciata*	Chunhuhub	-88.5638410151	19.5872409680	Quintana Roo	MEXICO	58
Lutzomyia	*serrana*	Chuxnaban	-95.7572222222	17.7647222222	Oaxaca	MEXICO	38
Lutzomyia	*shannoni*	Chuxnaban	-95.7572222222	17.7647222222	Oaxaca	MEXICO	38
Lutzomyia	*cruciata*	Chuxnaban	-95.8300000000	17.0177777778	Oaxaca	MEXICO	40
Lutzomyia	*deleoni*	Cinco de Mayo	-89.1267444102	18.7748866814	Quintana Roo	MEXICO	58
Brumptomyia	*mesai*	Cinco de Mayo	-89.1267444102	18.7748866814	Quintana Roo	MEXICO	58
Lutzomyia	*ovallesi*	Cinco de Mayo	-89.1267444102	18.7748866814	Quintana Roo	MEXICO	58
Lutzomyia	*permira*	Cinco de Mayo	-89.1267444102	18.7748866814	Quintana Roo	MEXICO	58
Lutzomyia	*shannoni*	Cinco de Mayo	-89.1267444102	18.7748866814	Quintana Roo	MEXICO	58
Lutzomyia	*panamensis*	Cinco de Mayo	-89.1267444102	18.7748866814	Quintana Roo	MEXICO	58

Lutzomyia	*steatopyga*	Cinco de Mayo	-89.1267444102	18.7748866814	Quintana Roo	MEXICO	58
Lutzomyia	*cayennensis*	Cinco de Mayo	-89.1266244690	18.7759087686	Quintana Roo	MEXICO	58
Lutzomyia	*deleoni*	Cinco de Mayo	-89.1266244690	18.7759087686	Quintana Roo	MEXICO	58
Lutzomyia	*longipalpis*	Cinco de Mayo	-89.1266244690	18.7759087686	Quintana Roo	MEXICO	58
Lutzomyia	*olmeca*	Cinco de Mayo	-89.1266244690	18.7759087686	Quintana Roo	MEXICO	58
Lutzomyia	*ovallesi*	Cinco de Mayo	-89.1266244690	18.7759087686	Quintana Roo	MEXICO	58
Lutzomyia	*panamensis*	Cinco de Mayo	-89.1266244690	18.7759087686	Quintana Roo	MEXICO	58
Lutzomyia	*shannoni*	Cinco de Mayo	-89.1266244690	18.7759087686	Quintana Roo	MEXICO	58
Lutzomyia	*cruciata*	Cinco de Mayo	-89.1267444102	18.7748866814	Quintana Roo	MEXICO	58
Lutzomyia	*cruciata*	Cinco de Mayo	-89.1266244690	18.7759087686	Quintana Roo	MEXICO	58
Lutzomyia	*shannoni*	Clinton Hids	-90.3217591667	32.3415833333	Mississippi	USA	88
Lutzomyia	*shannoni*	Coatzacoalcos	-94.4633333333	18.1358333333	Veracruz Ignacio de la Llave	MEXICO	40
Lutzomyia	*cayennensis*	Coba	-87.7098331209	20.4967340282	Quintana Roo	MEXICO	18, 58

Brumptomyia	*mesai*	Coba	-87.7098331209	20.4967340282	Quintana Roo	MEXICO	58
Lutzomyia	*shannoni*	Coba	-87.7098331209	20.4967340282	Quintana Roo	MEXICO	58
Lutzomyia	*steatopyga*	Coba	-87.7098331209	20.4967340282	Quintana Roo	MEXICO	58
Lutzomyia	*trinidadensis*	Coba	-87.7098331209	20.4967340282	Quintana Roo	MEXICO	58
Lutzomyia	*olmeca*	Coba	-87.7098331209	20.4967340282	Quintana Roo	MEXICO	18, 58
Lutzomyia	*cratifer*	Coba	-87.7098331209	20.4967340282	Quintana Roo	MEXICO	58
Lutzomyia	*undulata*	Coba	-87.7098331209	20.4967340282	Quintana Roo	MEXICO	58
Lutzomyia	*cayennensis*	Coba	-87.7306129576	20.5046443636	Quintana Roo	MEXICO	58
Lutzomyia	*cratifer*	Coba	-87.7306129576	20.5046443636	Quintana Roo	MEXICO	58
Lutzomyia	*deleoni*	Coba	-87.7306129576	20.5046443636	Quintana Roo	MEXICO	58
Lutzomyia	*longipalpis*	Coba	-87.7306129576	20.5046443636	Quintana Roo	MEXICO	58
Brumptomyia	*mesai*	Coba	-87.7306129576	20.5046443636	Quintana Roo	MEXICO	58
Lutzomyia	*olmeca*	Coba	-87.7306129576	20.5046443636	Quintana Roo	MEXICO	58

Lutzomyia	*ovallesi*	Coba	-87.7306129576	20.5046443636	Quintana Roo	MEXICO	58
Lutzomyia	*serrana*	Coba	-87.7306129576	20.5046443636	Quintana Roo	MEXICO	58
Lutzomyia	*steatopyga*	Coba	-87.7306129576	20.5046443636	Quintana Roo	MEXICO	58
Lutzomyia	*undulata*	Coba	-87.7306129576	20.5046443636	Quintana Roo	MEXICO	58
Lutzomyia	*deleoni*	Coba	-87.7098331209	20.4967340282	Quintana Roo	MEXICO	18
Lutzomyia	*shannoni*	Coba	-87.7098331209	20.4967340282	Quintana Roo	MEXICO	18
Lutzomyia	*panamensis*	Coba	-87.7352777778	20.4947222222	Quintana Roo	MEXICO	18
Lutzomyia	*cruciata*	Coba	-87.7098331209	20.4967340282	Quintana Roo	MEXICO	58
Lutzomyia	*cruciata*	Coba	-87.7306129576	20.5046443636	Quintana Roo	MEXICO	58
Lutzomyia	*cruciata*	Coba	-87.7098331209	20.4967340282	Quintana Roo	MEXICO	18
Lutzomyia	*ylephiletor*	Coban	-90.3715361111	15.4710666667	Alta Verapaz	GUATEMALA	47
Lutzomyia	*cruciata*	Coban	-90.3715361111	15.4710666667	Alta Verapaz	GUATEMALA	43, 57
Lutzomyia	*diabolica*	Cocula	-99.6636111111	18.2366666600	Guerrero	MEXICO	43, 96, 97

Lutzomyia	*dodgei*	Cocula	-99.6636111111	18.2366666600	Guerrero	MEXICO	96, 97
Lutzomyia	*cayennensis*	Cocula	-99.6636111111	18.2366666600	Guerrero	MEXICO	38
Lutzomyia	*shannoni*	Cocula	-99.6636111111	18.2366666600	Guerrero	MEXICO	40
Lutzomyia	*cruciata*	Cocula	-99.6636111111	18.2366666600	Guerrero	MEXICO	40
Lutzomyia	*diabolica*	Cola de Caballo	-100.1612222222	25.3846444440	Nuevo Leon	MEXICO	32
Lutzomyia	*cruciata*	Cola de Caballo	-100.1612222222	25.3846444440	Nuevo Leon	MEXICO	40
Lutzomyia	*olmeca*	Comacalco	-93.2247222222	18.2716666666	Tabasco	MEXICO	40
Lutzomyia	*diabolica*	Comstock	-101.1734388889	29.6843805556	Texas	USA	104, 105
Lutzomyia	*vexator*	Connecticut	-73.1004833333	41.6032055556	Connecticut	USA	2
Lutzomyia	*panamensis*	Cool Shade	-88.9666667000	17.1666667000	Distrito el Cayo	BELIZE	53
Lutzomyia	*bispinosa*	Cool Shade	-88.9666667000	17.1666667000	Distrito el Cayo	BELIZE	53
Lutzomyia	*shannoni*	Cool Shade	-88.9666667000	17.1666667000	Distrito el Cayo	BELIZE	53
Lutzomyia	*ovallesi*	Cool Shade	-88.9666667000	17.1666667000	Distrito el Cayo	BELIZE	53

Lutzomyia	*permira*	Cool Shade	-88.9666667000	17.1666667000	Distrito el Cayo	BELIZE	57, 99
Lutzomyia	*olmeca*	Cool Shade	-88.9666667000	17.1666667000	Distrito el Cayo	BELIZE	57, 99
Lutzomyia	*cruciata*	Cool Shade	-88.9666667000	17.1666667000	Distrito el Cayo	BELIZE	53
Lutzomyia	*shannoni*	Copiah County Game Management Area	-90.3736666667	31.8990833333	Mississippi	USA	39
Lutzomyia	*shannoni*	Corkscrew Swamp	-90.4974361111	35.9904111111	Arkansas	USA	105
Lutzomyia	*cruciata*	Corozal Pacifico	-96.5250000000	15.8836111111	Oaxaca	MEXICO	40
Lutzomyia	*diabolica*	Corpus Christi	-97.3963805556	27.8005833333	Texas	USA	104, 105
Lutzomyia	*shannoni*	Cossoleacaque	-94.6369400000	17.9952800000	Veracruz Ignacio de la Llave	MEXICO	50
Lutzomyia	*cruciata*	COSOMALOAPAN	-95.7908333333	18.3766666667	Veracruz Ignacio de la Llave	MEXICO	40
Lutzomyia	*olmeca*	Coyolar	-94.3688890000	17.9205560000	Veracruz Ignacio de la Llave	MEXICO	47
Lutzomyia	*californica*	Coyote Creek	-118.0127138889	33.8593222222	California	USA	105
Lutzomyia	*dodgei*	Cuautla	-98.9548555556	18.8126166666	Morelos	MEXICO	45, 96, 97
Lutzomyia	*texana*	Cuautla	-98.9548555556	18.8126166666	Morelos	MEXICO	105

Lutzomyia	*stewarti*	Culp Valley	-117.1570166667	32.7167388889	California	USA	8
Lutzomyia	*olmeca*	Cunduacan	-93.1755555556	18.0672222222	Tabasco	MEXICO	40
Lutzomyia	*cruciata*	Cunduacan	-93.1755555556	18.0672222222	Tabasco	MEXICO	40
Lutzomyia	*anthophora*	D'Anis Site 1	-99.2797638889	29.3304722222	Texas	USA	104, 105
Lutzomyia	*anthophora*	Del Rio	-100.3958666667	29.3707305556	Texas	USA	104, 105
Lutzomyia	*californica*	Del Rio	-100.3958666667	29.3707305556	Texas	USA	104, 105
Lutzomyia	*texana*	Del Rio	-100.3958666667	29.3707305556	Texas	USA	25, 104, 105
Lutzomyia	*diabolica*	D'Anis Site 1	-99.2797638889	29.3304722222	Texas	USA	104, 105
Lutzomyia	*anthophora*	D'Anis Site 2	-99.0350000000	29.0350000000	Texas	USA	61
Lutzomyia	*diabolica*	D'Anis Site 2	-99.0350000000	29.0350000000	Texas	USA	61
Lutzomyia	*texana*	D'Anis Site 2	-99.0350000000	29.0350000000	Texas	USA	61
Lutzomyia	*cruciata*	Dimas Lopez	-97.6363888855	20.1605555555	Puebla	MEXICO	43
Lutzomyia	*cruciata*	Dos Arroyo	-96.6919444444	19.8668416667	Veracruz Ignacio de la Llave	MEXICO	50

Lutzomyia	*permira*	Dos Lagunas	-89.5321111111	17.6796611111	Peten	GUATEMALA	45, 46, 57
Brumptomyia	*mesai*	Dos Lagunas Sur	-89.3366666667	17.9274999999	Campeche	MEXICO	73
Brumptomyia	*hamata*	Dos Lagunas Sur	-89.3366666667	17.9274999999	Campeche	MEXICO	73
Lutzomyia	*carpenteri*	Dos Lagunas Sur	-89.3366666667	17.9274999999	Campeche	MEXICO	73
Lutzomyia	*deleoni*	Dos Lagunas Sur	-89.3366666667	17.9274999999	Campeche	MEXICO	73
Lutzomyia	*olmeca*	Dos Lagunas Sur	-89.3366666667	17.9274999999	Campeche	MEXICO	73
Lutzomyia	*ovallesi*	Dos Lagunas Sur	-89.3366666667	17.9274999999	Campeche	MEXICO	73
Lutzomyia	*panamensis*	Dos Lagunas Sur	-89.3366666667	17.9274999999	Campeche	MEXICO	73
Lutzomyia	*shannoni*	Dos Lagunas Sur	-89.3366666667	17.9274999999	Campeche	MEXICO	73
Lutzomyia	*trinidadensis*	Dos Lagunas Sur	-89.3366666667	17.9274999999	Campeche	MEXICO	73
Lutzomyia	*ylephiletor*	Dos Lagunas Sur	-89.3366666667	17.9274999999	Campeche	MEXICO	73
Lutzomyia	*steatopyga*	Dos Lagunas Sur	-89.3366666667	17.9274999999	Campeche	MEXICO	73
Lutzomyia	*cruciata*	Dos Lagunas Sur	-89.3366666667	17.9274999999	Campeche	MEXICO	73

Brumptomyia	*mesai*	Dos Naciones	-89.3456666667	17.9833333333	Campeche	MEXICO	80, 83
Brumptomyia	*hamata*	Dos Naciones	-89.3456666667	17.9833333333	Campeche	MEXICO	80, 83
Lutzomyia	*deleoni*	Dos Naciones	-89.3456666667	17.9833333333	Campeche	MEXICO	80, 83
Lutzomyia	*carpenteri*	Dos Naciones	-89.3456666667	17.9833333333	Campeche	MEXICO	80, 83
Lutzomyia	*longipalpis*	Dos Naciones	-89.3456666667	17.9833333333	Campeche	MEXICO	80, 83
Lutzomyia	*shannoni*	Dos Naciones	-89.3456666667	17.9833333333	Campeche	MEXICO	80, 83
Lutzomyia	*undulata*	Dos Naciones	-89.3456666667	17.9833333333	Campeche	MEXICO	80, 83
Lutzomyia	*ylephiletor*	Dos Naciones	-89.3456666667	17.9833333333	Campeche	MEXICO	80, 83
Lutzomyia	*ovallesi*	Dos Naciones	-89.3456666667	17.9833333333	Campeche	MEXICO	80, 83
Lutzomyia	*panamensis*	Dos Naciones	-89.3456666667	17.9833333333	Campeche	MEXICO	80, 83
Lutzomyia	*trinidadensis*	Dos Naciones	-89.3456666667	17.9833333333	Campeche	MEXICO	80, 83
Lutzomyia	*olmeca*	Dos Naciones	-89.3456666667	17.9833333333	Campeche	MEXICO	80, 83
Lutzomyia	*cruciata*	Dos Naciones	-89.3456666667	17.9833333333	Campeche	MEXICO	80, 83

Lutzomyia	*shannoni*	Dumac (Ducks Unlimited de Mexico)	-90.3833333333	20.5833333333	Yucatan	MEXICO	40
Lutzomyia	*olmeca*	Dzibalché	-89.7313900000	19.4583300000	Campeche	MEXICO	83
Lutzomyia	*shannoni*	Dzibalché	-89.7313900000	19.4583300000	Campeche	MEXICO	83
Lutzomyia	*cruciata*	Dzibalché	-89.7313900000	19.4583300000	Campeche	MEXICO	83
Lutzomyia	*shannoni*	Ecoparaiso	-90.3702777778	20.9408333333	Yucatan	MEXICO	40
Lutzomyia	*shannoni*	Edith	-82.5512861111	30.6785638889	Georgia	USA	105
Lutzomyia	*panamensis*	El Ajengibre	-97.6786111111	20.4380555556	Puebla	MEXICO	40
Lutzomyia	*shannoni*	El Ajengibre	-97.6786111111	20.4380555556	Puebla	MEXICO	40
Lutzomyia	*cruciata*	El Ajengibre	-97.6786111111	20.4380555556	Puebla	MEXICO	40
Lutzomyia	*cayennensis*	El Carrizal	-97.2523250000	20.5987971889	Veracruz Ignacio de la Llave	MEXICO	48, 94
Lutzomyia	*cruciata*	El Coyul	-95.9063888889	16.5011111000	Oaxaca	MEXICO	38
Lutzomyia	*cayennensis*	El Cuyo	-87.6794444444	21.5125000000	Yucatan	MEXICO	38
Lutzomyia	*shannoni*	El Dorado	-92.6662666667	33.2076305556	Arkansas	USA	105

Lutzomyia	*shannoni*	El Gavilan	-90.7468060000	18.5872310000	Campeche	Mexico	3, 4
Lutzomyia	*panamensis*	El Gavilan	-90.7468060000	18.5872310000	Campeche	Mexico	3, 4
Lutzomyia	*cruciata*	El Gavilan	-90.7468060000	18.5872310000	Campeche	Mexico	3, 4
Lutzomyia	*shannoni*	El Lechugal	-90.7333330000	18.5638840000	Campeche	MEXICO	4
Lutzomyia	*panamensis*	El Lechugal	-90.7333330000	18.5638840000	Campeche	MEXICO	4
Lutzomyia	*cruciata*	El Lechugal	-90.6794430000	18.6510680000	Campeche	MEXICO	4
Lutzomyia	*shannoni*	El Limon	-94.4138888889	17.9627777777	Veracruz Ignacio de la Llave	MEXICO	38
Lutzomyia	*cratifer*	El Limon	-94.4138888889	17.9627777777	Veracruz Ignacio de la Llave	MEXICO	51
Lutzomyia	*shannoni*	El Nigromante	-95.7572200000	17.7647200000	Veracruz Ignacio de la Llave	MEXICO	50
Lutzomyia	*undulata*	El Nigromante	-95.7572200000	17.7647200000	Veracruz Ignacio de la Llave	MEXICO	50
Lutzomyia	*serrana*	El Nigromante	-95.7572200000	17.7647200000	Veracruz Ignacio de la Llave	MEXICO	38
Lutzomyia	*ovallesi*	Palenque	-93.8419444444	16.6313888889	Chiapas	MEXICO	32
Lutzomyia	*longipalpis*	Palenque	-93.8419444444	16.6313888889	Chiapas	MEXICO	40

Lutzomyia	*olmeca*	Palenque	-93.8419444444	16.6313888889	Chiapas	MEXICO	40
Lutzomyia	*panamensis*	Palenque	-93.8419444444	16.6313888889	Chiapas	MEXICO	40
Lutzomyia	*shannoni*	Palenque	-93.8419444444	16.6313888889	Chiapas	MEXICO	40
Lutzomyia	*ylephiletor*	Palenque	-93.8419444444	16.6313888889	Chiapas	MEXICO	40
Lutzomyia	*beltrani*	Palenque	-93.8419444444	16.6313888889	Chiapas	MEXICO	46
Lutzomyia	*permira*	El Palmar	-96.4405777778	19.2834722222	Veracruz Ignacio de la Llave	MEXICO	51
Lutzomyia	*carpenteri*	El Palmar	-96.4405777778	19.2834722222	Veracruz Ignacio de la Llave	MEXICO	51
Lutzomyia	*cruciata*	El Pensamiento	-96.6941944411	19.8390444411	Veracruz Ignacio de la Llave	MEXICO	50
Lutzomyia	*shannoni*	El Pital	-90.7523860000	18.5999990000	Campeche	MEXICO	4
Lutzomyia	*panamensis*	El Pital	-90.7523860000	18.5999990000	Campeche	MEXICO	4
Lutzomyia	*cruciata*	El Pital	-90.7523860000	18.5999990000	Campeche	MEXICO	4
Lutzomyia	*cruciata*	El Porvenir	-94.1944777778	16.3694000000	Oaxaca	MEXICO	38
Lutzomyia	*shannoni*	El Rifle	-90.7333330000	18.5638840000	Campeche	MEXICO	3, 4

Lutzomyia	*panamensis*	El Rifle	-90.7333330000	18.5638840000	Campeche	MEXICO	3, 4
Lutzomyia	*cruciata*	El Rifle	-90.7333330000	18.5638840000	Campeche	MEXICO	3, 4
Lutzomyia	*serrana*	El Tacahuite	-96.6594444444	19.8233333333	Veracruz Ignacio de la Llave	MEXICO	50
Lutzomyia	*shannoni*	El Tacahuite	-96.6594444444	19.8233333333	Veracruz Ignacio de la Llave	MEXICO	38
Lutzomyia	*cruciata*	El Tacahuite	-96.6594444444	19.8233333333	Veracruz Ignacio de la Llave	MEXICO	50
Lutzomyia	*cayennensis*	El Tepozal	-105.0661100000	21.3872200000	Nayarit	MEXICO	38
Lutzomyia	*ovallesi*	El Tepozal	-105.0661100000	21.3872200000	Nayarit	MEXICO	38
Lutzomyia	*shannoni*	El Tormento	-90.8095460000	18.5999850000	Campeche	MEXICO	3, 4
Lutzomyia	*panamensis*	El Tormento	-90.8095460000	18.5999850000	Campeche	MEXICO	3, 4
Lutzomyia	*cruciata*	El Tormento	-90.8095460000	18.5999850000	Campeche	MEXICO	3, 4
Lutzomyia	*shannoni*	El Trueno	-90.7872240000	18.6510680000	Campeche	MEXICO	4
Lutzomyia	*panamensis*	El Trueno	-90.7872240000	18.6510680000	Campeche	MEXICO	4
Lutzomyia	*cruciata*	El Trueno	-90.7872240000	18.6510680000	Campeche	MEXICO	4

Lutzomyia	*serrana*	El Zapotal	-96.6744444444	19.9550000000	Veracruz Ignacio de la Llave	MEXICO	50
Lutzomyia	*shannoni*	El Zapotal	-96.6744444444	19.9550000000	Veracruz Ignacio de la Llave	MEXICO	50
Lutzomyia	*cruciata*	El Zapotal	-96.6744444444	19.9550000000	Veracruz Ignacio de la Llave	MEXICO	50
Lutzomyia	*chiapanensis*	El Zapote	-96.1233333333	18.7683333333	Veracruz Ignacio de la Llave	MEXICO	38
Lutzomyia	*texana*	El Zopilote	-104.9511027777	21.9671194444	Nayarit	MEXICO	47, 93
Lutzomyia	*shannoni*	El Zopilote	-104.9511027777	21.9671194444	Nayarit	MEXICO	47, 93
Lutzomyia	*serrana*	El Zopilote	-104.9511027777	21.9671194444	Nayarit	MEXICO	38
Lutzomyia	*cayennensis*	El Zopilote	-104.9511027777	21.9671194444	Nayarit	MEXICO	38, 93
Lutzomyia	*cruciata*	El Zopilote	-104.9511027777	21.9671194444	Nayarit	MEXICO	43, 93
Lutzomyia	*shannoni*	Emiliano Zapata	-94.3686111111	17.9527777778	Veracruz Ignacio de la Llave	MEXICO	38
Lutzomyia	*olmeca*	Emiliano Zapata	-94.3686111111	17.9527777778	Veracruz Ignacio de la Llave	MEXICO	47
Lutzomyia	*cayennensis*	Escuintla	-92.6576333333	16.3221361110	Chiapas	MEXICO	98
Lutzomyia	*undulata*	Escuintla	-90.7869444444	14.2977194444	Escuintla	GUATEMALA	29

Brumptomyia	*mesai*	Esperanza	-89.1563889000	16.8936111000	Distrito el Cayo	BELIZE	57
Lutzomyia	*permira*	Esperanza	-89.1563889000	16.8936111000	Distrito el Cayo	BELIZE	57
Lutzomyia	*cayennensis*	Esperanza	-89.1563889000	16.8936111000	Distrito el Cayo	BELIZE	57
Lutzomyia	*trinidadensis*	Esperanza	-89.1563889000	16.8936111000	Distrito el Cayo	BELIZE	57
Lutzomyia	*beltrani*	Esperanza	-89.1563889000	16.8936111000	Distrito el Cayo	BELIZE	57
Lutzomyia	*shannoni*	Esperanza	-89.1563889000	16.8936111000	Distrito el Cayo	BELIZE	57
Lutzomyia	*undulata*	Esperanza	-89.1563889000	16.8936111000	Distrito el Cayo	BELIZE	57
Lutzomyia	*carpenteri*	Esperanza	-89.1563889000	16.8936111000	Distrito el Cayo	BELIZE	99, 100
Lutzomyia	*cruciata*	Esperanza	-89.1563889000	16.8936111000	Distrito el Cayo	BELIZE	57
Lutzomyia	*chiapanensis*	Espinal de Santa Barbara	-96.4197222222	19.1280555556	Veracruz Ignacio de la Llave	MEXICO	38
Lutzomyia	*shannoni*	Estancia de Morelos	-95.8911111111	17.0519444444	Oaxaca	MEXICO	38
Lutzomyia	*cruciata*	Estancia de Morelos	-95.8911111111	17.0519444444	Oaxaca	MEXICO	38
Lutzomyia	*shannoni*	Everglades National Park	-91.8318333333	35.2026138889	Arkansas	USA	105

Lutzomyia	*vexator*	Fairfax	-122.5888694444	37.9870277778	California	USA	8
Lutzomyia	*chiapanensis*	Farallon	-96.4102777778	19.6375000000	Veracruz Ignacio de la Llave	MEXICO	51
Lutzomyia	*shannoni*	Fayette Site1	-84.4801361111	38.0960361111	Kentucky	USA	67
Lutzomyia	*shannoni*	Fayette Site 2	-84.4802611111	37.9544458333	Ohio	USA	67
Lutzomyia	*deleoni*	Felipe Carrillo Puerto	-88.0454055556	19.5775594444	Quintana Roo	MEXICO	21, 45
Lutzomyia	*shannoni*	Felipe Carrillo Puerto	-88.0454055556	19.5775594444	Quintana Roo	MEXICO	20, 47
Lutzomyia	*trinidadensis*	Felipe Carrillo Puerto	-88.0454055556	19.5775594444	Quintana Roo	MEXICO	38
Lutzomyia	*cratifer*	Felipe Carrillo Puerto	-88.0454055556	19.5775594444	Quintana Roo	MEXICO	21
Lutzomyia	*longipalpis*	Felipe Carrillo Puerto	-88.0454055556	19.5775594444	Quintana Roo	MEXICO	20
Lutzomyia	*olmeca*	Felipe Carrillo Puerto	-88.0454055556	19.5775594444	Quintana Roo	MEXICO	5, 21
Lutzomyia	*ovallesi*	Felipe Carrillo Puerto	-88.0454055556	19.5775594444	Quintana Roo	MEXICO	5
Lutzomyia	*panamensis*	Felipe Carrillo Puerto	-88.0454055556	19.5775594444	Quintana Roo	MEXICO	5
Lutzomyia	*cruciata*	Felipe Carrillo Puerto	-88.0454055556	19.5775594444	Quintana Roo	MEXICO	43

Lutzomyia	*vexator*	Fillmore	-118.9181527778	34.3990888889	California	USA	8, 31
Lutzomyia	*carpenteri*	Finca Argovia	-92.2947777778	15.1246388889	Chiapas	MEXICO	66
Lutzomyia	*texana*	Finca Argovia	-92.2947777778	15.1246388889	Chiapas	MEXICO	66
Lutzomyia	*cruciata*	Finca Argovia	-92.2947777778	15.1246388889	Chiapas	MEXICO	66
Lutzomyia	*cruciata*	Finca Zapote	-90.5666666667	14.4166666667	Escuintla	GUATEMALA	105
Lutzomyia	*carpenteri*	Finca Guadalupe Saju	-92.2858333333	15.1569444444	Chiapas	MEXICO	66
Lutzomyia	*shannoni*	Finca Guadalupe Saju	-92.2858333333	15.1569444444	Chiapas	MEXICO	66
Lutzomyia	*ovallesi*	Finca Guadalupe Saju	-92.2858333333	15.1569444444	Chiapas	MEXICO	66
Lutzomyia	*cruciata*	Finca Guadalupe Saju	-92.2858333333	15.1569444444	Chiapas	MEXICO	66, 105
Lutzomyia	*cruciata*	Finca La Chiripa	-92.3063888889	15.1580555556	Chiapas	MEXICO	43
Lutzomyia	*longipalpis*	Finca Los Horcones	-93.6055555556	15.9555555556	Chiapas	MEXICO	43
Lutzomyia	*cruciata*	Finca San Basilio	-91.3167000000	14.4667000000	Suchitepequez	GUATEMALA	105
Lutzomyia	*shannoni*	Fork State Park	-84.1857111111	39.0928444444	Ohio	USA	67

Lutzomyia	*shannoni*	Fort Bragg	-78.9991666666	35.1391666667	North Caroline	USA	10, 34, 105
Lutzomyia	*vexator*	Fort Bragg	-78.9991666666	35.1391666667	North Caroline	USA	10, 34
Lutzomyia	*shannoni*	Fort Campbell	-87.4667333333	36.6499638889	Kentucky	USA	10, 11, 34, 35, 67
Lutzomyia	*vexator*	Fort Campbell	-87.4667333333	36.6499638889	Kentucky	USA	10, 34, 67
Lutzomyia	*shannoni*	Fort Hood	-97.7800000000	31.1297444444	Texas	USA	10
Lutzomyia	*vexator*	Fort Hood	-97.7800000000	31.1297444444	Texas	USA	10
Lutzomyia	*diabolica*	Fort Hood	-97.7800000000	31.1297444444	Texas	USA	10
Lutzomyia	*anthophora*	Fort Hood	-97.7800000000	31.1297444444	Texas	USA	10
Lutzomyia	*stewarti*	Fort Hunter Liggett	-121.2489750000	35.9767805556	California	USA	105
Lutzomyia	*shannoni*	Fort Johnson	-81.1637250000	33.8313138889	South Caroline	USA	105
Lutzomyia	*shannoni*	Fort McCoy	-81.9670083333	29.3649416667	Florida	USA	104, 105
Lutzomyia	*shannoni*	Fort Rucker	-85.7151416667	31.3433500000	Alabama	USA	34, 35, 105
Lutzomyia	*californica*	Fort Yuma	-114.6192027778	32.6957055556	California	USA	8, 105

Lutzomyia	*carpenteri*	Francisco I. Madero	-88.0419053624	20.2158719746	Quintana Roo	MEXICO	58
Lutzomyia	*cayennensis*	Francisco I. Madero	-88.0419053624	20.2158719746	Quintana Roo	MEXICO	58
Lutzomyia	*deleoni*	Francisco I. Madero	-88.0419053624	20.2158719746	Quintana Roo	MEXICO	58
Brumptomyia	*mesai*	Francisco I. Madero	-88.0419053624	20.2158719746	Quintana Roo	MEXICO	58
Brumptomyia	*hamata*	Francisco I. Madero	-88.0419053624	20.2158719746	Quintana Roo	MEXICO	58
Lutzomyia	*longipalpis*	Francisco I. Madero	-88.0419053624	20.2158719746	Quintana Roo	MEXICO	58
Lutzomyia	*shannoni*	Francisco I. Madero	-88.0419053624	20.2158719746	Quintana Roo	MEXICO	58
Lutzomyia	*steatopyga*	Francisco I. Madero	-88.0419053624	20.2158719746	Quintana Roo	MEXICO	58
Lutzomyia	*undulata*	Francisco I. Madero	-88.0419053624	20.2158719746	Quintana Roo	MEXICO	58
Lutzomyia	*cratifer*	Francisco I. Madero	-88.0419053624	20.2158719746	Quintana Roo	MEXICO	58
Lutzomyia	*olmeca*	Francisco I. Madero	-88.0419053624	20.2158719746	Quintana Roo	MEXICO	58
Lutzomyia	*carpenteri*	Francisco I. Madero	-88.0478438134	20.1606488075	Quintana Roo	MEXICO	58
Lutzomyia	*longipalpis*	Francisco I. Madero	-88.0478438134	20.1606488075	Quintana Roo	MEXICO	58

Lutzomyia	*cayennensis*	Francisco I. Madero	-88.0478438134	20.1606488075	Quintana Roo	MEXICO	58
Lutzomyia	*cratifer*	Francisco I. Madero	-88.0478438134	20.1606488075	Quintana Roo	MEXICO	58
Lutzomyia	*deleoni*	Francisco I. Madero	-88.0478438134	20.1606488075	Quintana Roo	MEXICO	58
Brumptomyia	*mesai*	Francisco I. Madero	-88.0478438134	20.1606488075	Quintana Roo	MEXICO	58
Lutzomyia	*shannoni*	Francisco I. Madero	-88.0478438134	20.1606488075	Quintana Roo	MEXICO	58
Lutzomyia	*steatopyga*	Francisco I. Madero	-88.0478438134	20.1606488075	Quintana Roo	MEXICO	58
Lutzomyia	*undulata*	Francisco I. Madero	-88.0478438134	20.1606488075	Quintana Roo	MEXICO	58
Lutzomyia	*olmeca*	Francisco I. Madero	-93.1589166667	18.1748055556	Tabasco	MEXICO	64
Lutzomyia	*cruciata*	Francisco I. Madero	-88.0419053624	20.2158719746	Quintana Roo	MEXICO	58
Lutzomyia	*cruciata*	Francisco I. Madero	-88.0478438134	20.1606488075	Quintana Roo	MEXICO	58
Lutzomyia	*diabolica*	Fredericksburg	-98.8719833330	30.2697611111	Texas	USA	104, 105
Lutzomyia	*texana*	Fredericksburg	-98.8719833330	30.2697611111	Texas	USA	104, 105
Lutzomyia	*anthophora*	Fresno Wash	-109.8275833333	32.8962000000	Arizona	USA	60

Lutzomyia	*californica*	Fresno Wash	-109.8275833333	32.8962000000	Arizona	USA	60
Lutzomyia	*shannoni*	Gainsville	-82.3248500000	29.6516333333	Florida	USA	27
Lutzomyia	*diabolica*	Gainsville	-82.3248500000	29.6516333333	Florida	USA	52
Lutzomyia	*cruciata*	Gainsville	-82.3248500000	29.6516333333	Florida	USA	27, 104, 105
Lutzomyia	*longipalpis*	Galecio Narcia	-93.0187333333	16.5698333333	Chiapas	MEXICO	49
Lutzomyia	*cruciata*	Galecio Narcia	-93.0187333333	16.5698333333	Chiapas	MEXICO	49
Lutzomyia	*panamensis*	Gallo Jug	-89.0518055556	17.5601017778	Orange Walk	BELIZE	57
Lutzomyia	*bispinosa*	Gallo Jug	-89.0518055556	17.5601017778	Orange Walk	BELIZE	57
Lutzomyia	*deleoni*	Gallo Jug	-89.0518055556	17.5601017778	Orange Walk	BELIZE	57
Lutzomyia	*olmeca*	Gallo Jug	-89.0518055556	17.5601017778	Orange Walk	BELIZE	57
Lutzomyia	*shannoni*	Gallo Jug	-89.0518055556	17.5601017778	Orange Walk	BELIZE	57
Lutzomyia	*ovallesi*	Gallo Jug	-89.0518055556	17.5601017778	Orange Walk	BELIZE	57
Lutzomyia	*cruciata*	Gallo Jug	-89.0518055556	17.5601017778	Orange Walk	BELIZE	57

Lutzomyia	*anthophora*	Garcia	-100.5975777778	25.8089083333	Nuevo Leon	MEXICO	105
Lutzomyia	*diabolica*	Garner State Park	-99.7861666667	29.2097111111	Texas	USA	104, 105
Lutzomyia	*texana*	Garner State Park	-99.7861666667	29.2097111111	Texas	USA	104, 105
Lutzomyia	*shannoni*	Garner State Park	-99.7861666667	29.2097111111	Texas	USA	52
Lutzomyia	*cruciata*	General Lazaro Cardenas	-95.8925000000	17.8402777778	Oaxaca	MEXICO	40
Lutzomyia	*shannoni*	Gral. Manuel Castilla Brito	-89.4315111111	18.4503500000	Campeche	MEXICO	78
Lutzomyia	*cruciata*	Gral. Manuel Castilla Brito	-89.4315111111	18.4503500000	Campeche	MEXICO	78
Lutzomyia	*longipalpis*	Granjas Merida	-99.0569444444	18.8641666667	Morelos	MEXICO	20, 71
Lutzomyia	*anthophora*	Granjas Merida	-99.0569444444	18.8641666667	Morelos	MEXICO	71
Lutzomyia	*cayennensis*	Granjas Merida	-99.0569444444	18.8641666667	Morelos	MEXICO	71
Lutzomyia	*diabolica*	Granjas Merida	-99.0569444444	18.8641666667	Morelos	MEXICO	71
Lutzomyia	*dodgei*	Granjas Merida	-99.0569444444	18.8641666667	Morelos	MEXICO	71
Lutzomyia	*texana*	Granjas Merida	-99.0569444444	18.8641666667	Morelos	MEXICO	71

Lutzomyia	*trinidadensis*	Granjas Merida	-99.0569444444	18.8641666667	Morelos	MEXICO	71
Lutzomyia	*shannoni*	Great Smoky Mountains National Park	-83.5345580556	35.6918444444	Tennessee	USA	41
Lutzomyia	*bispinosa*	Guacamallo	-89.0332805556	16.8665388889	Distrito el Cayo	BELIZE	53
Lutzomyia	*panamensis*	Guacamallo	-89.0332805556	16.8665388889	Distrito el Cayo	BELIZE	53
Lutzomyia	*ylephiletor*	Guacamallo	-89.0332805556	16.8665388889	Distrito el Cayo	BELIZE	53
Brumptomyia	*hamata*	Guacamallo	-89.0332805556	16.8665388889	Distrito el Cayo	BELIZE	53
Lutzomyia	*trinidadensis*	Guacamallo	-89.0332805556	16.8665388889	Distrito el Cayo	BELIZE	53
Lutzomyia	*shannoni*	Guacamallo	-89.0332805556	16.8665388889	Distrito el Cayo	BELIZE	53
Lutzomyia	*ovallesi*	Guacamallo	-89.0332805556	16.8665388889	Distrito el Cayo	BELIZE	53
Brumptomyia	*mesai*	Guacamallo	-89.0332805556	16.8665388889	Distrito el Cayo	BELIZE	57, 99
Lutzomyia	*deleoni*	Guacamallo	-89.0332805556	16.8665388889	Distrito el Cayo	BELIZE	103
Lutzomyia	*beltrani*	Guacamallo	-89.0332805556	16.8665388889	Distrito el Cayo	BELIZE	103
Lutzomyia	*cruciata*	Guacamallo	-89.0332805556	16.8665388889	Distrito el Cayo	BELIZE	53

Lutzomyia	*cruciata*	Guadalupe Miramar	-91.2078888889	16.1980555556	Chiapas	MEXICO	74
Lutzomyia	*shannoni*	Guadalupe Victoria	-95.8888888889	16.0160000000	Oaxaca	MEXICO	38
Lutzomyia	*cruciata*	Guadalupe Victoria	-95.8888888889	16.0160000000	Oaxaca	MEXICO	38
Lutzomyia	*longipalpis*	Guastatoya	-90.0666666667	14.8500138889	El Progreso	GUATEMALA	57
Lutzomyia	*panamensis*	Guatemala	-90.5352777778	14.6134472222		GUATEMALA	48
Lutzomyia	*trinidadensis*	Guatemala	-90.5352777778	14.6134472222	Guatemala	GUATEMALA	48
Lutzomyia	*serrana*	Guatemala	-90.5352777778	14.6134472222	Guatemala	GUATEMALA	44
Lutzomyia	*shannoni*	Gulf Hammock	-82.7309444444	29.2530083333	Florida	USA	104, 105
Lutzomyia	*vexator*	Gulf Hammock	-82.7309444444	29.2530083333	Florida	USA	27
Lutzomyia	*diabolica*	Gulf Hammock	-82.7309444444	29.2530083333	Florida	USA	52
Lutzomyia	*stewarti*	Hasting Reservation	-121.8946750000	36.6001888889	California	USA	8
Lutzomyia	*vexator*	Hasting Reservation	-121.8946750000	36.6001888889	California	USA	8
Lutzomyia	*chiapanensis*	Hermosillo	-110.9613083333	29.0890694444	Sonora	MEXICO	38

Lutzomyia	*stewarti*	Hermosillo	-110.9613083333	29.0890694444	Sonora	MEXICO	48
Lutzomyia	*vexator*	Hermosillo	-110.9613083333	29.0890694444	Sonora	MEXICO	48
Lutzomyia	*diabolica*	Hidalgo del Parral	-105.6668333333	26.9323166660	Chihuahua	MEXICO	105
Lutzomyia	*shannoni*	Hinds	-90.3748361111	32.2652388889	Mississippi	USA	39
Brumptomyia	*hamata*	Hobonil	-89.0416670000	20.0016670000	Yucatan	MEXICO	69
Lutzomyia	*beltrani*	Hobonil	-89.0416670000	20.0016670000	Yucatan	MEXICO	69
Lutzomyia	*cayennensis*	Hobonil	-89.0416670000	20.0016670000	Yucatan	MEXICO	69
Lutzomyia	*cratifer*	Hobonil	-89.0416670000	20.0016670000	Yucatan	MEXICO	69
Lutzomyia	*deleoni*	Hobonil	-89.0416670000	20.0016670000	Yucatan	MEXICO	69
Lutzomyia	*serrana*	Hobonil	-89.0416670000	20.0016670000	Yucatan	MEXICO	69
Lutzomyia	*trinidadensis*	Hobonil	-89.0416670000	20.0016670000	Yucatan	MEXICO	69
Lutzomyia	*shannoni*	Honey Island Woods	-89.9253222222	30.4357416667	Louisiana	USA	87
Lutzomyia	*vexator*	Hooper	-122.1616722222	41.2679333333	California	USA	31

Lutzomyia	*shannoni*	Hot Springs	-88.2022888889	30.7629000000	Alabama	USA	105
Lutzomyia	*diabolica*	Huetamo	-100.9066305556	18.6329722222	Michoacan de Ocampo	MEXICO	43
Lutzomyia	*longipalpis*	Huetamo	-100.9066305556	18.6329722222	Michoacan de Ocampo	MEXICO	43
Lutzomyia	*cayennensis*	Huetamo	-100.9066305556	18.6329722222	Michoacan de Ocampo	MEXICO	38
Lutzomyia	*cruciata*	Huetamo	-100.9066305556	18.6329722222	Michoacan de Ocampo	MEXICO	38
Lutzomyia	*olmeca*	Huimanguillo	-93.3913888889	17.8302777778	Tabasco	MEXICO	40
Lutzomyia	*longipalpis*	Huitzuco	-99.3317400000	18.3012900000	Guerrero	MEXICO	20, 71
Lutzomyia	*cayennensis*	Huitzuco	-99.3317400000	18.3012900000	Guerrero	MEXICO	71
Lutzomyia	*diabolica*	Huitzuco	-99.3317400000	18.3012900000	Guerrero	MEXICO	71
Lutzomyia	*dodgei*	Huitzuco	-99.3317400000	18.3012900000	Guerrero	MEXICO	71
Lutzomyia	*texana*	Huitzuco	-99.3317400000	18.3012900000	Guerrero	MEXICO	71
Lutzomyia	*trinidadensis*	Huitzuco	-99.3317400000	18.3012900000	Guerrero	MEXICO	71
Lutzomyia	*cruciata*	Huitzuco	-99.3317400000	18.3012900000	Guerrero	MEXICO	40

Brumptomyia	*mesai*	Humminbird Highway	-88.7131555556	17.1520944444	Distrito el Cayo	BELIZE	57
Lutzomyia	*panamensis*	Humminbird Highway	-88.7131555556	17.1520944444	Distrito el Cayo	BELIZE	57
Lutzomyia	*cayennensis*	Humminbird Highway	-88.7131555556	17.1520944444	Distrito el Cayo	BELIZE	57
Lutzomyia	*trinidadensis*	Humminbird Highway	-88.7131555556	17.1520944444	Distrito el Cayo	BELIZE	57
Lutzomyia	*deleoni*	Humminbird Highway	-88.7131555556	17.1520944444	Distrito el Cayo	BELIZE	57
Lutzomyia	*olmeca*	Humminbird Highway	-88.7131555556	17.1520944444	Distrito el Cayo	BELIZE	57
Lutzomyia	*ovallesi*	Humminbird Highway	-88.7131555556	17.1520944444	Distrito el Cayo	BELIZE	57
Lutzomyia	*cruciata*	Humminbird Highway	-88.7131555556	17.1520944444	Distrito el Cayo	BELIZE	57
Lutzomyia	*cayennensis*	Iguala de la Independencia	-99.5333333333	18.3558388883	Guerrero	MEXICO	95
Lutzomyia	*dodgei*	Iguala de la Independencia	-99.5333333333	18.3558388883	Guerrero	MEXICO	45, 96, 97
Lutzomyia	*texana*	Iguala de la Independencia	-99.5333333333	18.3558388883	Guerrero	MEXICO	105
Brumptomyia	*mesai*	Iguana Creek	-89.0000000000	17.3333805555	Distrito el Cayo	BELIZE	57, 99
Lutzomyia	*permira*	Iguana Creek	-89.0000000000	17.3333805555	Distrito el Cayo	BELIZE	57, 99

Lutzomyia	*cayennensis*	Iguana Creek	-89.0000000000	17.3333805555	Distrito el Cayo	BELIZE	57, 99
Lutzomyia	*trinidadensis*	Iguana Creek	-89.0000000000	17.3333805555	Distrito el Cayo	BELIZE	57, 99
Lutzomyia	*cruciata*	Iguana Creek	-89.0000000000	17.3333805555	Distrito el Cayo	BELIZE	57, 99
Lutzomyia	*vexator*	Institute of Ecosystem Studies	-73.6940444444	41.7850722222	New York	USA	72
Lutzomyia	*carpenteri*	Ixil	-89.4816611111	21.1509583333	Yucatan	MEXICO	84
Lutzomyia	*cayennensis*	Ixil	-89.4816611111	21.1509583333	Yucatan	MEXICO	84
Lutzomyia	*cruciata*	Ixil	-89.4816611111	21.1509583333	Yucatan	MEXICO	84
Lutzomyia	*longipalpis*	Ixtapa	-101.5511111111	17.6411111111	Guerrero	MEXICO	43
Lutzomyia	*cruciata*	Ixtapa	-101.5511111111	17.6411111111	Guerrero	MEXICO	43
Lutzomyia	*shannoni*	Jacksonville	-77.4302416667	34.7540527778	North Caroline	USA	105
Lutzomyia	*olmeca*	Jalpa de Mendez	-93.0625000000	18.1763888889	Tabasco	MEXICO	40
Lutzomyia	*texana*	Jimenez	-100.6771861111	29.0693555556	Coahuila de Zaragoza	MEXICO	38
Lutzomyia	*deleoni*	Jose Maria Morelos	-88.7208143841	19.7541928932	Quintana Roo	MEXICO	70

Brumptomyia	*mesai*	Jose Maria Morelos	-88.7208143841	19.7541928932	Quintana Roo	MEXICO	70
Brumptomyia	*hamata*	Jose Maria Morelos	-88.7208143841	19.7541928932	Quintana Roo	MEXICO	70
Lutzomyia	*shannoni*	Jose Maria Morelos	-88.7208143841	19.7541928932	Quintana Roo	MEXICO	70
Lutzomyia	*panamensis*	Jose Maria Morelos	-88.7208143841	19.7541928932	Quintana Roo	MEXICO	70
Lutzomyia	*cruciata*	Jose Maria Morelos	-88.7208143841	19.7541928932	Quintana Roo	MEXICO	70
Lutzomyia	*anthophora*	Jourdanton	-98.5465833333	28.9180277778	Texas	USA	59
Lutzomyia	*cruciata*	Juan Diaz	-97.8525000000	21.4097222222	Veracruz Ignacio de la Llave	MEXICO	40
Lutzomyia	*cruciata*	Juan Diaz Covarrubias	-95.1844444444	18.1586111111	Veracruz Ignacio de la Llave	MEXICO	38
Lutzomyia	*cruciata*	Juchique de Ferrer	-96.6942499997	19.8390499967	Veracruz Ignacio de la Llave	MEXICO	50
Lutzomyia	*shannoni*	Kenner	-90.2417416667	29.9940916667	Louisiana	USA	105
Lutzomyia	*shannoni*	Knox Site 1	-83.9371805556	36.1272277778	Tennessee	USA	41
Lutzomyia	*shannoni*	Knox Site 10	-83.5030777777	35.6922388889	Tennessee	USA	41
Lutzomyia	*shannoni*	Knox Site 11	-83.5640638889	36.1651388888	Tennessee	USA	41

Lutzomyia	*shannoni*	Knox Site 2	-83.9806083333	36.1018194444	Tennessee	USA	41
Lutzomyia	*shannoni*	Knox Site 3	-83.9593416667	36.0379666667	Tennessee	USA	41
Lutzomyia	*shannoni*	Knox Site 4	-83.8988472222	36.0097555556	Tennessee	USA	41
Lutzomyia	*shannoni*	Knox Site 5	-84.1189194444	35.9564666667	Tennessee	USA	41
Lutzomyia	*shannoni*	Knox Site 6	-84.1107750000	35.8993555556	Tennessee	USA	41
Lutzomyia	*shannoni*	Knox Site 7	-83.9008444444	35.8701611111	Tennessee	USA	41
Lutzomyia	*shannoni*	Knox Site 8	-83.4750861111	35.4891638889	Tennessee	USA	41
Lutzomyia	*vexator*	Knox Site 8	-83.4750861111	35.4891638889	Tennessee	USA	41
Lutzomyia	*shannoni*	Knox Site 9	-83.8829361111	36.1708722222	Tennessee	USA	41
Lutzomyia	*vexator*	Knox Site 9	-83.8829361111	36.1708722222	Tennessee	USA	41
Lutzomyia	*shannoni*	La Bolita	-104.9308333333	21.9016666667	Nayarit	MEXICO	38
Lutzomyia	*cruciata*	La Bolita	-104.9308333333	21.9016666667	Nayarit	MEXICO	38
Lutzomyia	*cruciata*	La Cieneguita	-104.9552800000	20.7622200000	Jalisco	MEXICO	40

Lutzomyia	*cruciata*	La Granja	-92.5695561111	15.2127250000	Chiapas	MEXICO	105
Lutzomyia	*deleoni*	La Guadalupe	-89.4748333333	18.3373333333	Campeche	MEXICO	65, 80, 83
Lutzomyia	*ovallesi*	La Guadalupe	-89.4748333333	18.3373333333	Campeche	MEXICO	65, 80, 83
Lutzomyia	*panamensis*	La Guadalupe	-89.4748333333	18.3373333333	Campeche	MEXICO	65, 80, 83
Lutzomyia	*shannoni*	La Guadalupe	-89.4748333333	18.3373333333	Campeche	MEXICO	65, 80, 83
Lutzomyia	*undulata*	La Guadalupe	-89.4748333333	18.3373333333	Campeche	MEXICO	65, 80, 83
Brumptomyia	*mesai*	La Guadalupe	-89.4748333333	18.3373333333	Campeche	MEXICO	65, 80, 83
Lutzomyia	*carpenteri*	La Guadalupe	-89.4748333333	18.3373333333	Campeche	MEXICO	65, 80, 83
Lutzomyia	*trinidadensis*	La Guadalupe	-89.4748333333	18.3373333333	Campeche	MEXICO	65, 80, 83
Lutzomyia	*olmeca*	La Guadalupe	-89.4748333333	18.3373333333	Campeche	MEXICO	65, 80, 83
Lutzomyia	*cruciata*	La Guadalupe	-89.4748333333	18.3373333333	Campeche	MEXICO	65, 80, 83
Brumptomyia	*mesai*	La LIbertad	-90.4648333333	18.5266666667	Campeche	MEXICO	83
Brumptomyia	*hamata*	La LIbertad	-90.4648333333	18.5266666667	Campeche	MEXICO	43, 83

Lutzomyia	*carpenteri*	La LIbertad	-90.4648333333	18.5266666667	Campeche	MEXICO	83
Lutzomyia	*cayennensis*	La LIbertad	-90.4648333333	18.5266666667	Campeche	MEXICO	83
Lutzomyia	*deleoni*	La LIbertad	-90.4648333333	18.5266666667	Campeche	MEXICO	83
Lutzomyia	*longipalpis*	La LIbertad	-90.4648333333	18.5266666667	Campeche	MEXICO	83
Lutzomyia	*olmeca*	La LIbertad	-90.4648333333	18.5266666667	Campeche	MEXICO	83
Lutzomyia	*panamensis*	La LIbertad	-90.4648333333	18.5266666667	Campeche	MEXICO	83
Lutzomyia	*shannoni*	La LIbertad	-90.4648333333	18.5266666667	Campeche	MEXICO	83
Lutzomyia	*ovallesi*	La LIbertad	-90.4648333333	18.5266666667	Campeche	MEXICO	83
Lutzomyia	*serrana*	La LIbertad	-90.4648333333	18.5266666667	Campeche	MEXICO	83
Lutzomyia	*cayennensis*	La LIbertad	-90.5132222222	18.5675333333	Campeche	MEXICO	76
Lutzomyia	*carpenteri*	La LIbertad	-90.5132222222	18.5675333333	Campeche	MEXICO	79
Lutzomyia	*cratifer*	La LIbertad	-90.5132222222	18.5675333333	Campeche	MEXICO	79
Lutzomyia	*deleoni*	La LIbertad	-90.5132222222	18.5675333333	Campeche	MEXICO	79

Lutzomyia	*dodgei*	La LIbertad	-90.5132222222	18.5675333333	Campeche	MEXICO	79
Lutzomyia	*olmeca*	La LIbertad	-90.5132222222	18.5675333333	Campeche	MEXICO	79
Lutzomyia	*ovallesi*	La LIbertad	-90.5132222222	18.5675333333	Campeche	MEXICO	79
Lutzomyia	*panamensis*	La LIbertad	-90.5132222222	18.5675333333	Campeche	MEXICO	79
Lutzomyia	*permira*	La LIbertad	-90.5132222222	18.5675333333	Campeche	MEXICO	79
Lutzomyia	*serrana*	La LIbertad	-90.5132222222	18.5675333333	Campeche	MEXICO	79
Lutzomyia	*shannoni*	La LIbertad	-90.5132222222	18.5675333333	Campeche	MEXICO	79
Lutzomyia	*trinidadensis*	La LIbertad	-90.5132222222	18.5675333333	Campeche	MEXICO	79
Lutzomyia	*undulata*	La LIbertad	-90.5132222222	18.5675333333	Campeche	MEXICO	79
Lutzomyia	*cruciata*	La LIbertad	-90.5132222222	18.5675333333	Campeche	MEXICO	79
Lutzomyia	*cruciata*	La LIbertad	-90.4648333333	18.5266666667	Campeche	MEXICO	81, 82, 83
Lutzomyia	*longipalpis*	La Mina	-99.1794444444	18.3541666667	Guerrero	MEXICO	20
Lutzomyia	*shannoni*	La Mina	-104.9347222222	21.9600000000	Nayarit	MEXICO	38

Lutzomyia	*shannoni*	La Paz Camalote	-94.2686111111	17.9527777778	Veracruz Ignacio de la Llave	MEXICO	38
Lutzomyia	*diabolica*	La Pechera	-99.6250000000	25.7161111111	Nuevo Leon	MEXICO	43
Lutzomyia	*cruciata*	La Playita	-93.2588900000	18.1630600000	Tabasco	MEXICO	43
Lutzomyia	*panamensis*	La Porfia	-90.7333330000	18.5097100000	Campeche	MEXICO	4
Lutzomyia	*shannoni*	La Porfia	-90.7333330000	18.5097100000	Campeche	MEXICO	4
Lutzomyia	*cruciata*	La Porfia	-90.7333330000	18.5097100000	Campeche	MEXICO	4
Lutzomyia	*diabolica*	La Salle	-96.6691444444	28.8664444444	Texas	USA	59, 78
Lutzomyia	*shannoni*	La Sirena	-96.6525000000	15.9713888889	Oaxaca	MEXICO	40
Lutzomyia	*cruciata*	La Toma	-96.6691666667	17.0869444444	Oaxaca	MEXICO	40
Lutzomyia	*chiapanensis*	La Tranca	-96.2550000000	18.7669444444	Veracruz Ignacio de la Llave	MEXICO	38
Lutzomyia	*carpenteri*	La Virgencita	-89.3100000000	18.2397220000	Campeche	MEXICO	65
Lutzomyia	*deleoni*	La Virgencita	-89.3100000000	18.2397220000	Campeche	MEXICO	65
Lutzomyia	*longipalpis*	La Virgencita	-89.3100000000	18.2397220000	Campeche	MEXICO	65

Lutzomyia	*olmeca*	La Virgencita	-89.3100000000	18.2397220000	Campeche	MEXICO	65
Lutzomyia	*ovallesi*	La Virgencita	-89.3100000000	18.2397220000	Campeche	MEXICO	65
Lutzomyia	*panamensis*	La Virgencita	-89.3100000000	18.2397220000	Campeche	MEXICO	65
Lutzomyia	*shannoni*	La Virgencita	-89.3100000000	18.2397220000	Campeche	MEXICO	65
Lutzomyia	*trinidadensis*	La Virgencita	-89.3100000000	18.2397220000	Campeche	MEXICO	65
Brumptomyia	*mesai*	La Virgencita	-89.3100000000	18.2397220000	Campeche	MEXICO	65
Lutzomyia	*cayennensis*	La Virgencita	-89.3100000000	18.2397220000	Campeche	MEXICO	65
Lutzomyia	*cruciata*	La Virgencita	-89.3100000000	18.2397220000	Campeche	MEXICO	65
Lutzomyia	*anthophora*	Lackland	-98.5811111111	29.3841527778	Texas	USA	61
Lutzomyia	*diabolica*	Lackland	-98.5811111111	29.3841527778	Texas	USA	61
Lutzomyia	*texana*	Lackland	-98.5811111111	29.3841527778	Texas	USA	61
Lutzomyia	*anthophora*	Laguna Atascosa Wildlife Refuge	-97.3853055556	26.2869694444	Texas	USA	104, 105
Lutzomyia	*diabolica*	Laguna Atascosa Wildlife Refuge	-97.3853055556	26.2869694444	Texas	USA	104, 105

Lutzomyia	*beltrani*	Laguna Encantada	-95.2012250000	18.4446111111	Veracruz Ignacio de la Llave	MEXICO	94
Lutzomyia	*shannoni*	Laguna Encantada	-95.2012250000	18.4446111111	Veracruz Ignacio de la Llave	MEXICO	40
Lutzomyia	*shannoni*	Laguna Guerrero	-88.2982318160	18.6839885440	Quintana Roo	MEXICO	58
Lutzomyia	*carpenteri*	Laguna Guerrero	-88.2982318160	18.6839885440	Quintana Roo	MEXICO	58
Lutzomyia	*cayennensis*	Laguna Guerrero	-88.2982318160	18.6839885440	Quintana Roo	MEXICO	58
Lutzomyia	*deleoni*	Laguna Guerrero	-88.2982318160	18.6839885440	Quintana Roo	MEXICO	58
Lutzomyia	*olmeca*	Laguna Guerrero	-88.2982318160	18.6839885440	Quintana Roo	MEXICO	58
Lutzomyia	*permira*	Laguna Guerrero	-88.2982318160	18.6839885440	Quintana Roo	MEXICO	58
Lutzomyia	*deleoni*	Laguna Guerrero	-88.3087426237	18.6809787922	Quintana Roo	MEXICO	58
Lutzomyia	*olmeca*	Laguna Guerrero	-88.3087426237	18.6809787922	Quintana Roo	MEXICO	58
Lutzomyia	*shannoni*	Laguna Guerrero	-88.3087426237	18.6809787922	Quintana Roo	MEXICO	58
Lutzomyia	*cruciata*	Laguna Guerrero	-88.2982318160	18.6839885440	Quintana Roo	MEXICO	58
Lutzomyia	*cruciata*	Laguna Guerrero	-88.3087426237	18.6809787922	Quintana Roo	MEXICO	58

Lutzomyia	*deleoni*	Lagunitas	-88.3052777778	18.5036111111	Quintana Roo	MEXICO	38
Lutzomyia	*olmeca*	Lagunitas	-88.3052777778	18.5036111111	Quintana Roo	MEXICO	40
Lutzomyia	*diabolica*	Lake Buchanan	-98.4293888889	30.7938861111	Texas	USA	104, 105
Lutzomyia	*anthophora*	Laredo	-99.5075416667	27.5061944444	Texas	USA	38
Lutzomyia	*diabolica*	Laredo	-99.5075416667	27.5061944444	Texas	USA	38
Lutzomyia	*diabolica*	Las Adjuntas	-100.3194500000	25.4168055550	Nuevo Leon	MEXICO	78
Lutzomyia	*diabolica*	Las Bajadas	-96.1867750000	19.1450722222	Veracruz Ignacio de la Llave	MEXICO	23
Lutzomyia	*cruciata*	Las Bajadas	-96.1867750000	19.1450722222	Veracruz Ignacio de la Llave	MEXICO	40
Lutzomyia	*dodgei*	Las Colonias	-102.3544555555	19.1071916666	Michoacan de Ocampo	MEXICO	45, 96, 97
Lutzomyia	*shannoni*	Las Laminas	-96.3625000000	16.2588888800	Oaxaca	MEXICO	40
Lutzomyia	*cruciata*	Las Laminas	-96.3625000000	16.2588888800	Oaxaca	MEXICO	38
Lutzomyia	*chiapanensis*	Las Limas	-96.3741666667	18.8383333333	Veracruz Ignacio de la Llave	MEXICO	38, 51
Lutzomyia	*shannoni*	Las Limas	-96.3741666667	18.8383333333	Veracruz Ignacio de la Llave	MEXICO	38

Lutzomyia	*anthophora*	Las Majadas	-102.4071222222	19.1250194444	Michoacan de Ocampo	MEXICO	46
Lutzomyia	*shannoni*	Las Pilas	-94.7888888889	17.0450000000	Nayarit	MEXICO	38, 40
Lutzomyia	*serrana*	Las Pilas	-94.7888888889	17.0450000000	Nayarit	MEXICO	38
Lutzomyia	*cruciata*	Las Pilas	-94.7888888889	17.0450000000	Nayarit	MEXICO	38
Lutzomyia	*shannoni*	Leake	-89.4742166667	32.8073500000	Mississippi	USA	39
Lutzomyia	*deleoni*	Leona Vicario	-87.2000000000	20.9902777778	Quintana Roo	MEXICO	18
Lutzomyia	*olmeca*	Leona Vicario	-87.2000000000	20.9902777778	Quintana Roo	MEXICO	18
Lutzomyia	*panamensis*	Leona Vicario	-87.2000000000	20.9902777778	Quintana Roo	MEXICO	18
Lutzomyia	*cayennensis*	Leona Vicario	-87.2000000000	20.9902777778	Quintana Roo	MEXICO	18
Lutzomyia	*shannoni*	Leona Vicario	-87.2000000000	20.9902777778	Quintana Roo	MEXICO	18
Lutzomyia	*trinidadensis*	Leona Vicario	-87.2000000000	20.9902777778	Quintana Roo	MEXICO	18
Lutzomyia	*cruciata*	Leona Vicario	-87.2000000000	20.9902777778	Quintana Roo	MEXICO	18
Lutzomyia	*olmeca*	Libertad	-93.1712552778	18.0719555556	Tabasco	MEXICO	64

Lutzomyia	*shannoni*	Little Rock Air Force Base	-92.1468583333	34.9115916667	Arkansas	USA	59
Lutzomyia	*vexator*	Livermore	-121.7680083333	37.6818277778	California	USA	8, 56
Lutzomyia	*stewarti*	Livermore	-121.7680083333	37.6818277778	California	USA	8, 56
Lutzomyia	*cruciata*	Llano Ciruelo	-96.3041666667	15.7977777770	Oaxaca	MEXICO	38
Lutzomyia	*longipalpis*	Llano Grande	-95.0592861111	17.0465972221	Puebla	MEXICO	20, 71
Lutzomyia	*cayennensis*	Llano Grande	-95.0592861111	17.0465972221	Puebla	MEXICO	71
Lutzomyia	*diabolica*	Llano Grande	-95.0592861111	17.0465972221	Puebla	MEXICO	71
Lutzomyia	*dodgei*	Llano Grande	-95.0592861111	17.0465972221	Puebla	MEXICO	71
Lutzomyia	*texana*	Llano Grande	-95.0592861111	17.0465972221	Puebla	MEXICO	71
Lutzomyia	*trinidadensis*	Llano Grande	-95.0592861111	17.0465972221	Puebla	MEXICO	71
Lutzomyia	*cruciata*	Llano Juarez	-96.3198972222	15.8301472222	Oaxaca	MEXICO	43
Lutzomyia	*cruciata*	Loma Bonita	-91.2078888889	16.1980555556	Chiapas	MEXICO	74
Lutzomyia	*cruciata*	Loma de San Nicolas	-96.6941944444	19.8428472222	Veracruz Ignacio de la Llave	MEXICO	50

Lutzomyia	*cruciata*	Lomas de los Ingleses	-95.1741666667	18.1408333333	Veracruz Ignacio de la Llave	MEXICO	38
Lutzomyia	*shannoni*	Longfellow-Evangeline State Park	-91.8218638889	30.1363638889	Louisiana	USA	87
Lutzomyia	*diabolica*	Los Rodriguez	-101.5177000000	27.8754888882	Coahuila de Zaragoza	MEXICO	23, 43
Lutzomyia	*texana*	Los Rodriguez	-101.5177000000	27.8754888882	Coahuila de Zaragoza	MEXICO	38
Lutzomyia	*beltrani*	Macario Gomez	-87.5381327963	20.3103244916	Quintana Roo	MEXICO	58
Lutzomyia	*cayennensis*	Macario Gomez	-87.5381327963	20.3103244916	Quintana Roo	MEXICO	58
Lutzomyia	*cratifer*	Macario Gomez	-87.5381327963	20.3103244916	Quintana Roo	MEXICO	58
Lutzomyia	*deleoni*	Macario Gomez	-87.5381327963	20.3103244916	Quintana Roo	MEXICO	58
Brumptomyia	*mesai*	Macario Gomez	-87.5381327963	20.3103244916	Quintana Roo	MEXICO	58
Lutzomyia	*olmeca*	Macario Gomez	-87.5381327963	20.3103244916	Quintana Roo	MEXICO	58
Lutzomyia	*ovallesi*	Macario Gomez	-87.5381327963	20.3103244916	Quintana Roo	MEXICO	58
Lutzomyia	*shannoni*	Macario Gomez	-87.5381327963	20.3103244916	Quintana Roo	MEXICO	58
Lutzomyia	*steatopyga*	Macario Gomez	-87.5381327963	20.3103244916	Quintana Roo	MEXICO	58

Lutzomyia	*trinidadensis*	Macario Gomez	-87.5381327963	20.3103244916	Quintana Roo	MEXICO	58
Lutzomyia	*undulata*	Macario Gomez	-87.5381327963	20.3103244916	Quintana Roo	MEXICO	58
Lutzomyia	*cayennensis*	Macario Gomez	-87.5337053878	20.3096784193	Quintana Roo	MEXICO	58
Lutzomyia	*deleoni*	Macario Gomez	-87.5337053878	20.3096784193	Quintana Roo	MEXICO	58
Lutzomyia	*steatopyga*	Macario Gomez	-87.5337053878	20.3096784193	Quintana Roo	MEXICO	58
Lutzomyia	*undulata*	Macario Gomez	-87.5337053878	20.3096784193	Quintana Roo	MEXICO	58
Lutzomyia	*cratifer*	Macario Gomez	-87.5337053878	20.3096784193	Quintana Roo	MEXICO	58
Brumptomyia	*mesai*	Macario Gomez	-87.5337053878	20.3096784193	Quintana Roo	MEXICO	58
Lutzomyia	*longipalpis*	Macario Gomez	-87.5337053878	20.3096784193	Quintana Roo	MEXICO	58
Lutzomyia	*olmeca*	Macario Gomez	-87.5337053878	20.3096784193	Quintana Roo	MEXICO	58
Lutzomyia	*ovallesi*	Macario Gomez	-87.5337053878	20.3096784193	Quintana Roo	MEXICO	58
Lutzomyia	*shannoni*	Macario Gomez	-87.5337053878	20.3096784193	Quintana Roo	MEXICO	58
Lutzomyia	*cruciata*	Macario Gomez	-87.5381327963	20.3103244916	Quintana Roo	MEXICO	58

Lutzomyia	*cruciata*	Macario Gomez	-87.5337053878	20.3096784193	Quintana Roo	MEXICO	58
Brumptomyia	*mesai*	Veinte de Noviembre	-89.3002777778	18.8144444444	Campeche	MEXICO	73
Lutzomyia	*carpenteri*	Veinte de Noviembre	-89.3002777778	18.8144444444	Campeche	MEXICO	73
Lutzomyia	*olmeca*	Veinte de Noviembre	-89.3002777778	18.8144444444	Campeche	MEXICO	73
Lutzomyia	*ovallesi*	Veinte de Noviembre	-89.3002777778	18.8144444444	Campeche	MEXICO	73
Lutzomyia	*panamensis*	Veinte de Noviembre	-89.3002777778	18.8144444444	Campeche	MEXICO	73
Lutzomyia	*shannoni*	Veinte de Noviembre	-89.3002777778	18.8144444444	Campeche	MEXICO	73
Lutzomyia	*steatopyga*	Veinte de Noviembre	-89.3002777778	18.8144444444	Campeche	MEXICO	73
Lutzomyia	*trinidadensis*	Veinte de Noviembre	-89.3002777778	18.8144444444	Campeche	MEXICO	73
Lutzomyia	*deleoni*	Veinte de Noviembre	-89.3002777778	18.8144444444	Campeche	MEXICO	73
Lutzomyia	*permira*	Veinte de Noviembre	-89.3002777778	18.8144444444	Campeche	MEXICO	73
Lutzomyia	*cruciata*	Veinte de Noviembre	-89.3002777778	18.8144444444	Campeche	MEXICO	73
Lutzomyia	*shannoni*	Mansfield	-93.7001833333	32.0376388889	Louisiana	USA	105

Lutzomyia	*stewarti*	March Creek	-121.7325916667	37.9110194444	California	USA	8, 56, 105
Lutzomyia	*vexator*	March Creek	-121.7325916667	37.9110194444	California	USA	31, 56
Lutzomyia	*californica*	Marfa	-104.0298583333	30.3079250000	Texas	USA	26
Lutzomyia	*vexator*	Marfa	-104.0298583333	30.3079250000	Texas	USA	26
Lutzomyia	*anthophora*	Marfa	-104.0298583333	30.3079250000	Texas	USA	26
Lutzomyia	*panamensis*	Matamoros	-90.6486100000	18.5833333333	Campeche	MEXICO	3, 4
Lutzomyia	*shannoni*	Matamoros	-90.6486100000	18.5833333333	Campeche	MEXICO	4
Lutzomyia	*cruciata*	Matamoros	-90.6486100000	18.5833333333	Campeche	MEXICO	4, 105
Lutzomyia	*chiapanensis*	Medellin	-95.3675000000	18.4163888889	Veracruz Ignacio de la Llave	MEXICO	51
Lutzomyia	*carpenteri*	Medellin	-95.3675000000	18.4163888889	Veracruz Ignacio de la Llave	MEXICO	51
Lutzomyia	*longipalpis*	Merida-Xmatkuil	-89.6250083333	20.8668361111	Yucatan	MEXICO	84
Lutzomyia	*cruciata*	Merida-Xmatkuil	-89.6250083333	20.8668361111	Yucatan	MEXICO	43
Lutzomyia	*anthophora*	Miacatlan	-99.3537277777	18.7715083327	Morelos	MEXICO	105

Lutzomyia	*diabolica*	Miacatlan	-99.3537277777	18.7715083327	Morelos	MEXICO	105
Lutzomyia	*texana*	Miacatlan	-99.3537277777	18.7715083327	Morelos	MEXICO	105
Lutzomyia	*steatopyga*	Millionario	-88.9948000000	16.7435638889	Distrito el Cayo	BELIZE	99, 100
Lutzomyia	*beltrani*	Millionario	-88.9948000000	16.7435638889	Distrito el Cayo	BELIZE	101, 102
Lutzomyia	*deleoni*	Millionario	-88.9948000000	16.7435638889	Distrito el Cayo	BELIZE	101, 102
Lutzomyia	*shannoni*	Millionario	-88.9948000000	16.7435638889	Distrito el Cayo	BELIZE	101, 102
Brumptomyia	*mesai*	Millionario	-88.9948000000	16.7435638889	Distrito el Cayo	BELIZE	57, 99
Brumptomyia	*hamata*	Millionario	-88.9948000000	16.7435638889	Distrito el Cayo	BELIZE	57, 99
Lutzomyia	*panamensis*	Millionario	-88.9948000000	16.7435638889	Distrito el Cayo	BELIZE	57, 99
Lutzomyia	*bispinosa*	Millionario	-88.9948000000	16.7435638889	Distrito el Cayo	BELIZE	57, 99
Lutzomyia	*trinidadensis*	Millionario	-88.9948000000	16.7435638889	Distrito el Cayo	BELIZE	57, 99
Lutzomyia	*ovallesi*	Millionario	-88.9948000000	16.7435638889	Distrito el Cayo	BELIZE	57, 99
Lutzomyia	*cruciata*	Millionario	-88.9948000000	16.7435638889	Distrito el Cayo	BELIZE	57, 99

Lutzomyia	*cruciata*	Montebello	-96.3597222222	18.0611111111	Oaxaca	MEXICO	40
Lutzomyia	*shannoni*	Montgomery	-83.8897055556	38.0314583333	Tennessee	USA	67
Lutzomyia	*vexator*	Montgomery	-83.8897055556	38.0314583333	Tennessee	USA	67
Lutzomyia	*shannoni*	Monticello	-83.8701638889	30.5452027778	Florida	USA	104, 105
Lutzomyia	*panamensis*	Mountain Pine Ridge	-88.8000000000	16.9833333000	Distrito el Cayo	BELIZE	57, 99
Lutzomyia	*trinidadensis*	Mountain Pine Ridge	-88.8000000000	16.9833333000	Distrito el Cayo	BELIZE	57, 99
Lutzomyia	*deleoni*	Mountain Pine Ridge	-88.8000000000	16.9833333000	Distrito el Cayo	BELIZE	57, 99
Lutzomyia	*olmeca*	Mountain Pine Ridge	-88.8000000000	16.9833333000	Distrito el Cayo	BELIZE	57, 99
Lutzomyia	*carpenteri*	Mountain Pine Ridge	-88.8000000000	16.9833333000	Distrito el Cayo	BELIZE	99, 100
Lutzomyia	*cruciata*	Mountain Pine Ridge	-88.8000000000	16.9833333000	Distrito el Cayo	BELIZE	57, 99
Lutzomyia	*cratifer*	Nautla	-96.7729222222	20.2066194444	Veracruz Ignacio de la Llave	MEXICO	51
Lutzomyia	*shannoni*	Nautla	-96.7729222222	20.2066194444	Veracruz Ignacio de la Llave	MEXICO	51
Brumptomyia	*mesai*	Never Delay	-88.7657833333	17.3167055555	Distrito el Cayo	BELIZE	57

Lutzomyia	*panamensis*	Never Delay	-88.7657833333	17.3167055555	Distrito el Cayo	BELIZE	57
Lutzomyia	*permira*	Never Delay	-88.7657833333	17.3167055555	Distrito el Cayo	BELIZE	57
Lutzomyia	*cayennensis*	Never Delay	-88.7657833333	17.3167055555	Distrito el Cayo	BELIZE	57
Lutzomyia	*trinidadensis*	Never Delay	-88.7657833333	17.3167055555	Distrito el Cayo	BELIZE	57
Lutzomyia	*beltrani*	Never Delay	-88.7657833333	17.3167055555	Distrito el Cayo	BELIZE	57
Lutzomyia	*deleoni*	Never Delay	-88.7657833333	17.3167055555	Distrito el Cayo	BELIZE	57
Lutzomyia	*olmeca*	Never Delay	-88.7657833333	17.3167055555	Distrito el Cayo	BELIZE	57
Lutzomyia	*shannoni*	Never Delay	-88.7657833333	17.3167055555	Distrito el Cayo	BELIZE	57
Lutzomyia	*ovallesi*	Never Delay	-88.7657833333	17.3167055555	Distrito el Cayo	BELIZE	57
Lutzomyia	*cruciata*	Never Delay	-88.7657833333	17.3167055555	Distrito el Cayo	BELIZE	57
Lutzomyia	*shannoni*	New Orleans	-90.0715333333	29.9510666667	Louisiana	USA	105
Lutzomyia	*anthophora*	Nocupetaro de Morelos	-101.1620277770	19.0435138556	Michoacan de Ocampo	MEXICO	96, 97
Lutzomyia	*diabolica*	Nocupetaro de Morelos	-101.1620277770	19.0435138556	Michoacan de Ocampo	MEXICO	43

Lutzomyia	*cruciata*	Nocupetaro de Morelos	-101.1620277770	19.0435138556	Michoacan de Ocampo	MEXICO	38
Brumptomyia	*mesai*	Noh-Bec	-87.8083333333	19.2305555556	Quintana Roo	MEXICO	43
Lutzomyia	*ovallesi*	Noh-Bec	-87.8083333333	19.2305555556	Quintana Roo	MEXICO	43
Lutzomyia	*cruciata*	Noh-Bec	-87.8083333333	19.2305555556	Quintana Roo	MEXICO	43
Brumptomyia	*hamata*	Nuevo Becal	-89.2973055552	18.6303611104	Campeche	MEXICO	69
Lutzomyia	*carpenteri*	Nuevo Becal	-89.2973055552	18.6303611104	Campeche	MEXICO	69
Lutzomyia	*deleoni*	Nuevo Becal	-89.2973055552	18.6303611104	Campeche	MEXICO	69
Lutzomyia	*olmeca*	Nuevo Durango	-87.5895459714	20.7452226524	Quintana Roo	MEXICO	70
Lutzomyia	*shannoni*	Nuevo Durango	-87.5895459714	20.7452226524	Quintana Roo	MEXICO	70
Lutzomyia	*steatopyga*	Nuevo Durango	-87.5895459714	20.7452226524	Quintana Roo	MEXICO	70
Lutzomyia	*undulata*	Nuevo Durango	-87.5895459714	20.7452226524	Quintana Roo	MEXICO	70
Lutzomyia	*cruciata*	Nuevo Durango	-87.5895459714	20.7452226524	Quintana Roo	MEXICO	70
Lutzomyia	*cruciata*	Nuevo Montecristo	-93.3047220000	16.9355560000	Chiapas	MEXICO	74

Lutzomyia	*undulata*	Rio	-93.3750000000	16.7625000000	Chiapas	MEXICO	32
Lutzomyia	*olmeca*	Ocozocoautla	-93.3750000000	16.7625000000	Chiapas	MEXICO	40
Lutzomyia	*shannoni*	Ocozocoautla	-93.3750000000	16.7625000000	Chiapas	MEXICO	40
Lutzomyia	*ylephiletor*	Ocozocoautla	-93.3750000000	16.7625000000	Chiapas	MEXICO	40
Lutzomyia	*cratifer*	Ocozocoautla	-93.3750000000	16.7625000000	Chiapas	MEXICO	47
Lutzomyia	*shannoni*	O'Leno State Park	-82.6536527778	29.9191416667	Florida	USA	104, 105
Lutzomyia	*carpenteri*	Once de Mayo	-89.4608330000	18.0913890000	Campeche	MEXICO	75
Lutzomyia	*deleoni*	Once de Mayo	-89.4608330000	18.0913890000	Campeche	MEXICO	75
Lutzomyia	*longipalpis*	Once de Mayo	-89.4608330000	18.0913890000	Campeche	MEXICO	75
Lutzomyia	*olmeca*	Once de Mayo	-89.4608330000	18.0913890000	Campeche	MEXICO	75
Lutzomyia	*panamensis*	Once de Mayo	-89.4608330000	18.0913890000	Campeche	MEXICO	75
Lutzomyia	*shannoni*	Once de Mayo	-89.4608330000	18.0913890000	Campeche	MEXICO	75
Lutzomyia	*undulata*	Once de Mayo	-89.4608330000	18.0913890000	Campeche	MEXICO	75

Lutzomyia	*cruciata*	Once de Mayo	-89.4608330000	18.0913890000	Campeche	MEXICO	75
Brumptomyia	*mesai*	Opichen	-89.8573194444	20.5501194444	Yucatan	MEXICO	84
Lutzomyia	*deleoni*	Opichen	-89.8573194444	20.5501194444	Yucatan	MEXICO	84
Lutzomyia	*trinidadensis*	Opichen	-89.8573194444	20.5501194444	Yucatan	MEXICO	84
Lutzomyia	*cruciata*	Opichen	-89.8573194444	20.5501194444	Yucatan	MEXICO	84
Lutzomyia	*shannoni*	Ossabaw Island	-81.0945472222	31.8010527778	Georgia	USA	7, 12, 13, 14, 15, 16
Lutzomyia	*vexator*	Ossabaw Island	-81.0945472222	31.8010527778	Georgia	USA	105
Lutzomyia	*californica*	Othello	-119.1752916667	46.8258305556	Washington	USA	33, 105
Lutzomyia	*vexator*	Othello	-119.1752916667	46.8258305556	Washington	USA	33
Lutzomyia	*californica*	P.C. Boyd Desert Research Center	-117.3961555556	33.9530527778	California	USA	105
Lutzomyia	*shannoni*	Paint Creek State Park	-84.6505027778	39.3122888889	Ohio	USA	67
Lutzomyia	*longipalpis*	Pala	-99.3494444444	18.3316666667	Guerrero	MEXICO	20
Lutzomyia	*permira*	PALENQUE	-93.8419400000	16.6313900000	Chiapas	MEXICO	30

Lutzomyia	*steatopyga*	PALENQUE	-93.8419400000	16.6313900000	Chiapas	MEXICO	32
Lutzomyia	*cratifer*	PALENQUE	-93.8419400000	16.6313900000	Chiapas	MEXICO	47
Lutzomyia	*trinidadensis*	PALENQUE	-93.8419400000	16.6313900000	Chiapas	MEXICO	48
Lutzomyia	*longipalpis*	PALENQUE	-93.8419400000	16.6313900000	Chiapas	MEXICO	43
Lutzomyia	*serrana*	PALENQUE	-93.8419400000	16.6313900000	Chiapas	MEXICO	43
Lutzomyia	*cruciata*	PALENQUE	-93.8419400000	16.6313900000	Chiapas	MEXICO	105
Lutzomyia	*stewarti*	Palm Desert	-116.3744555556	33.7222444444	California	USA	105
Lutzomyia	*chiapanensis*	Palmas de Abajo	-96.4334111111	19.5831000000	Veracruz Ignacio de la Llave	MEXICO	51
Lutzomyia	*cruciata*	Panales	-103.8811111111	22.5905555556	Jalisco	MEXICO	43
Lutzomyia	*cruciata*	Papaloapan	-96.0947222222	18.1591666666	Oaxaca	MEXICO	40
Lutzomyia	*ovallesi*	Parque Nacional Tikal	-89.5916138889	17.2165472222	Peten	GUATEMALA	85, 86
Lutzomyia	*panamensis*	Parque Nacional Tikal	-89.5916138889	17.2165472222	Peten	GUATEMALA	85, 86
Lutzomyia	*shannoni*	Parque Nacional Tikal	-89.5916138889	17.2165472222	Peten	GUATEMALA	85, 86

Lutzomyia	*undulata*	Parque Nacional Tikal	-89.5916138889	17.2165472222	Peten	GUATEMALA	85, 86
Lutzomyia	*ylephiletor*	Parque Nacional Tikal	-89.5916138889	17.2165472222	Peten	GUATEMALA	85, 86
Lutzomyia	*serrana*	Parque Nacional Tikal	-89.5916138889	17.2165472222	Peten	GUATEMALA	86
Lutzomyia	*olmeca*	Parque Nacional Tikal	-89.5916138889	17.2165472222	Peten	GUATEMALA	86
Lutzomyia	*deleoni*	Parque Nacional Tikal	-89.5916138889	17.2165472222	Peten	GUATEMALA	86
Lutzomyia	*trinidadensis*	Parque Nacional Tikal	-89.5916138889	17.2165472222	Peten	GUATEMALA	86
Lutzomyia	*cruciata*	Parque Nacional Tikal	-89.5916138889	17.2165472222	Peten	GUATEMALA	85, 86, 105
Lutzomyia	*cruciata*	Paso Limon	-96.6822222222	15.8833333333	Oaxaca	MEXICO	38
Lutzomyia	*shannoni*	Patuxent National Wildlife Refuge	-76.7476805556	38.5399777778	Maryland	USA	34
Lutzomyia	*anthophora*	Pearsall	-99.0950333333	28.8921611111	Texas	USA	59
Lutzomyia	*diabolica*	Pearsall	-99.0950333333	28.8921611111	Texas	USA	59
Lutzomyia	*shannoni*	Pennville	-75.5165888889	39.6536833333	New Jersey	USA	76
Lutzomyia	*vexator*	Perth (Black Lake)	-76.2485666667	44.8988816667	Ontario	CANADA	24

Lutzomyia	*carpenteri*	Petcacab	-88.2097491850	19.2487770251	Quintana Roo	MEXICO	58
Lutzomyia	*deleoni*	Petcacab	-88.2097491850	19.2487770251	Quintana Roo	MEXICO	58
Brumptomyia	*mesai*	Petcacab	-88.2097491850	19.2487770251	Quintana Roo	MEXICO	58
Lutzomyia	*shannoni*	Petcacab	-88.2097491850	19.2487770251	Quintana Roo	MEXICO	58
Lutzomyia	*undulata*	Petcacab	-88.2097491850	19.2487770251	Quintana Roo	MEXICO	58
Lutzomyia	*olmeca*	Petcacab	-88.2097491850	19.2487770251	Quintana Roo	MEXICO	58
Lutzomyia	*carpenteri*	Petcacab	-88.2133003371	19.2517082312	Quintana Roo	MEXICO	58
Brumptomyia	*mesai*	Petcacab	-88.2133003371	19.2517082312	Quintana Roo	MEXICO	58
Lutzomyia	*steatopyga*	Petcacab	-88.2133003371	19.2517082312	Quintana Roo	MEXICO	58
Lutzomyia	*deleoni*	Petcacab	-88.2133003371	19.2517082312	Quintana Roo	MEXICO	58
Lutzomyia	*olmeca*	Petcacab	-88.2133003371	19.2517082312	Quintana Roo	MEXICO	58
Lutzomyia	*shannoni*	Petcacab	-88.2133003371	19.2517082312	Quintana Roo	MEXICO	58
Lutzomyia	*cruciata*	Petcacab	-88.2097491850	19.2487770251	Quintana Roo	MEXICO	58

Lutzomyia	*cruciata*	Petcacab	-88.2133003371	19.2517082312	Quintana Roo	MEXICO	58
Lutzomyia	*olmeca*	Peten	-90.2995777778	16.9093055556	Peten	GUATEMALA	57
Lutzomyia	*cruciata*	Peten	-90.2995777778	16.9093055556	Peten	GUATEMALA	43
Lutzomyia	*cruciata*	Piedra Cuache	-96.7016666666	16.7980555556	Oaxaca	MEXICO	40
Lutzomyia	*shannoni*	Pine Bluff	-86.5113777778	34.6609250000	Alabama	USA	105
Lutzomyia	*cruciata*	Piñal	-96.3711100000	15.7255600000	Oaxaca	MEXICO	43
Lutzomyia	*carpenteri*	Pipiapan	-95.2125000000	18.4497222222	Veracruz Ignacio de la Llave	MEXICO	51
Lutzomyia	*diabolica*	Plainview	-101.7067222222	34.1847944444	Texas	USA	104, 105
Lutzomyia	*beltrani*	Playa Escondida	-95.1112166666	18.4216805549	Veracruz Ignacio de la Llave	MEXICO	38, 45
Lutzomyia	*vexator*	Plummer's Island	-77.1763694444	38.9695555556	Maryland	USA	31, 38
Lutzomyia	*vexator*	Police Coulee	-116.5765027778	53.9379222222	Alberta	CANADA	91
Lutzomyia	*shannoni*	Poptun	-89.4227833333	16.3272944444	Peten	GUATEMALA	77
Lutzomyia	*olmeca*	Poptun	-89.4227833333	16.3272944444	Peten	GUATEMALA	77

Lutzomyia	*ylephiletor*	Poptun	-89.4227833333	16.3272944444	Peten	GUATEMALA	77
Lutzomyia	*bispinosa*	Poptun	-89.4227833333	16.3272944444	Peten	GUATEMALA	77
Lutzomyia	*panamensis*	Poptun	-89.4227833333	16.3272944444	Peten	GUATEMALA	77
Lutzomyia	*cruciata*	Poptun	-89.4227833333	16.3272944444	Peten	GUATEMALA	77
Lutzomyia	*diabolica*	Poteet	-98.5680750000	29.0405250000	Texas	USA	104, 105
Lutzomyia	*shannoni*	Princeton	-87.8819583333	37.1093583333	Kentucky	USA	68
Lutzomyia	*vexator*	Princeton	-87.8819583333	37.1093583333	Kentucky	USA	68
Lutzomyia	*shannoni*	Prussia Ridge	-83.0361388889	39.0713472222	Ohio	USA	67
Lutzomyia	*cayennensis*	Puente Jula	-96.3488888888	19.2000000000	Veracruz Ignacio de la Llave	MEXICO	51
Lutzomyia	*chiapanensis*	Puente Jula	-96.3488888888	19.2000000000	Veracruz Ignacio de la Llave	MEXICO	51
Lutzomyia	*shannoni*	Puerto Arturo	-89.0626172504	19.6781767101	Quintana Roo	MEXICO	58
Lutzomyia	*steatopyga*	Puerto Arturo	-89.0626172504	19.6781767101	Quintana Roo	MEXICO	58
Lutzomyia	*cratifer*	Puerto Arturo	-89.0626172504	19.6781767101	Quintana Roo	MEXICO	58

Lutzomyia	*shannoni*	Puerto Arturo	-89.0626172504	19.6781767101	Quintana Roo	MEXICO	58
Lutzomyia	*undulata*	Puerto Arturo	-89.0626172504	19.6781767101	Quintana Roo	MEXICO	58
Lutzomyia	*deleoni*	Puerto Arturo	-89.0660322464	19.6656275088	Quintana Roo	MEXICO	58
Brumptomyia	*mesai*	Puerto Arturo	-89.0660322464	19.6656275088	Quintana Roo	MEXICO	58
Lutzomyia	*shannoni*	Puerto Arturo	-89.0660322464	19.6656275088	Quintana Roo	MEXICO	58
Lutzomyia	*cratifer*	Puerto Arturo	-89.0660322464	19.6656275088	Quintana Roo	MEXICO	58
Lutzomyia	*undulata*	Puerto Arturo	-89.0660322464	19.6656275088	Quintana Roo	MEXICO	58
Lutzomyia	*cruciata*	Puerto Arturo	-89.0626172504	19.6781767101	Quintana Roo	MEXICO	58
Lutzomyia	*cruciata*	Puerto Arturo	-89.0660322464	19.6656275088	Quintana Roo	MEXICO	58
Lutzomyia	*cayennensis*	Puerto San Jose	-90.8210222222	13.9289166667	Escuintla	GUATEMALA	48, 57
Lutzomyia	*undulata*	Puerto San Jose	-90.8210222222	13.9289166667	Escuintla	GUATEMALA	29, 32, 57, 105
Lutzomyia	*cruciata*	Puerto San Jose	-90.8210222222	13.9289166667	Escuintla	GUATEMALA	57, 105
Lutzomyia	*dodgei*	Purificacion	-104.6031944444	19.7175861104	Jalisco	Mexico	46

Lutzomyia	*panamensis*	Putchituk Camp	-89.0583333333	16.7672222222	Distrito el Cayo	BELIZE	53
Lutzomyia	*bispinosa*	Putchituk Camp	-89.0583333333	16.7672222222	Distrito el Cayo	BELIZE	53
Lutzomyia	*permira*	Putchituk Camp	-89.0583333333	16.7672222222	Distrito el Cayo	BELIZE	57
Lutzomyia	*olmeca*	Putchituk Camp	-89.0583333333	16.7672222222	Distrito el Cayo	BELIZE	57
Lutzomyia	*shannoni*	Quincy	-84.5832444444	30.5854305556	Florida	USA	104, 105
Lutzomyia	*carpenteri*	Rancho La Ceiba	-88.7747807238	19.8582289761	Quintana Roo	MEXICO	20
Lutzomyia	*olmeca*	Rancho La Ceiba	-88.7747807238	19.8582289761	Quintana Roo	MEXICO	20
Lutzomyia	*ovallesi*	Rancho La Ceiba	-88.7747807238	19.8582289761	Quintana Roo	MEXICO	21
Lutzomyia	*panamensis*	Rancho La Ceiba	-88.7747807238	19.8582289761	Quintana Roo	MEXICO	21
Lutzomyia	*shannoni*	Rancho La Ceiba	-88.7747807238	19.8582289761	Quintana Roo	MEXICO	20
Lutzomyia	*cruciata*	Rancho La Ceiba	-88.7747807238	19.8582289761	Quintana Roo	MEXICO	20
Lutzomyia	*chiapanensis*	Rancho La Lomita	-90.3833333333	20.5833333333	Yucatan	MEXICO	48
Lutzomyia	*diabolica*	Rancho Las Flores	-99.1831250000	25.9242805555	Nuevo Leon	MEXICO	78

Lutzomyia	*stewarti*	Rancho Mirage, Magnesis Springs Canyon	-116.4155305556	33.7350388889	California	USA	105
Lutzomyia	*cruciata*	Rancho Pipiapan	-95.0602777778	18.4450000000	Veracruz Ignacio de la Llave	MEXICO	51
Lutzomyia	*shannoni*	Reelfoot Lake	-89.4221972222	36.3860888889	Tennessee	USA	92
Lutzomyia	*longipalpis*	Rincon del Lucero	-99.3298260000	18.2808450000	Guerrero	MEXICO	20
Lutzomyia	*shannoni*	Rio Cazones	-98.2008277778	19.0120416667	Puebla	MEXICO	3
Lutzomyia	*panamensis*	Rio Grande	-88.7773305556	16.1607194444	Distrito Toledo	BELIZE	57
Lutzomyia	*trinidadensis*	Rio Grande	-88.7773305556	16.1607194444	Distrito Toledo	BELIZE	57
Lutzomyia	*deleoni*	Rio Grande	-88.7773305556	16.1607194444	Distrito Toledo	BELIZE	57
Lutzomyia	*olmeca*	Rio Grande	-88.7773305556	16.1607194444	Distrito Toledo	BELIZE	57
Lutzomyia	*cratifer*	Rio Grande	-88.7773305556	16.1607194444	Distrito Toledo	BELIZE	57
Lutzomyia	*shannoni*	Rio Grande	-88.7773305556	16.1607194444	Distrito Toledo	BELIZE	57
Lutzomyia	*ovallesi*	Rio Grande	-88.7773305556	16.1607194444	Distrito Toledo	BELIZE	57
Lutzomyia	*cruciata*	Rio Grande	-88.7773305556	16.1607194444	Distrito Toledo	BELIZE	57

Lutzomyia	*cruciata*	Rio Platanar	-96.5586111111	15.9133333333	Oaxaca	MEXICO	40
Lutzomyia	*cruciata*	Rio Sarabia	-94.9333330000	17.1833330000	Oaxaca	MEXICO	43
Lutzomyia	*dodgei*	Rio Sabanal	-93.0762666660	16.7732166600	Chiapas	MEXICO	30
Lutzomyia	*shannoni*	Rio Sordo	-96.8111583333	17.2466666667	Oaxaca	MEXICO	38
Lutzomyia	*cruciata*	Rio Verde	-94.7888888889	17.0450000000	Oaxaca	MEXICO	38
Lutzomyia	*shannoni*	River Styx	-82.2406722222	29.5348805556	Florida	USA	104, 105
Lutzomyia	*steatopyga*	Roaring River	-88.7984722222	17.2330377778	Distrito el Cayo	BELIZE	99, 100
Lutzomyia	*trinidadensis*	Roaring River	-88.7984722222	17.2330377778	Distrito el Cayo	BELIZE	99, 100
Lutzomyia	*shannoni*	Roaring River	-88.7984722222	17.2330377778	Distrito el Cayo	BELIZE	99, 100
Brumptomyia	*mesai*	Roaring River	-88.7984722222	17.2330377778	Distrito el Cayo	BELIZE	57, 99
Lutzomyia	*panamensis*	Roaring River	-88.7984722222	17.2330377778	Distrito el Cayo	BELIZE	57, 99
Lutzomyia	*bispinosa*	Roaring River	-88.7984722222	17.2330377778	Distrito el Cayo	BELIZE	57, 99
Lutzomyia	*permira*	Roaring River	-88.7984722222	17.2330377778	Distrito el Cayo	BELIZE	57, 99

Lutzomyia	*cayennensis*	Roaring River	-88.7984722222	17.2330377778	Distrito el Cayo	BELIZE	57, 99
Lutzomyia	*beltrani*	Roaring River	-88.7984722222	17.2330377778	Distrito el Cayo	BELIZE	57, 99
Lutzomyia	*deleoni*	Roaring River	-88.7984722222	17.2330377778	Distrito el Cayo	BELIZE	57, 99
Lutzomyia	*olmeca*	Roaring River	-88.7984722222	17.2330377778	Distrito el Cayo	BELIZE	57, 99
Lutzomyia	*ovallesi*	Roaring River	-88.7984722222	17.2330377778	Distrito el Cayo	BELIZE	57, 99
Lutzomyia	*carpenteri*	Roaring River	-88.7984722222	17.2330377778	Distrito el Cayo	BELIZE	99, 100
Lutzomyia	*ylephiletor*	Roaring River	-88.7984722222	17.2330377778	Distrito el Cayo	BELIZE	103
Lutzomyia	*cruciata*	Roaring River	-88.7984722222	17.2330377778	Distrito el Cayo	BELIZE	57, 99
Lutzomyia	*shannoni*	Rose Hill	-78.0228916667	34.8282000000	North Caroline	USA	105
Lutzomyia	*carpenteri*	Saban	-88.4571559523	19.9675808880	Quintana Roo	MEXICO	58
Brumptomyia	*mesai*	Saban	-88.4571559523	19.9675808880	Quintana Roo	MEXICO	58
Lutzomyia	*shannoni*	Saban	-88.4571559523	19.9675808880	Quintana Roo	MEXICO	58
Lutzomyia	*undulata*	Saban	-88.4571559523	19.9675808880	Quintana Roo	MEXICO	58

Lutzomyia	*cratifer*	Saban	-88.4571559523	19.9675808880	Quintana Roo	MEXICO	58
Lutzomyia	*cratifer*	Saban	-88.4961852661	19.9843399712	Quintana Roo	MEXICO	58
Lutzomyia	*deleoni*	Saban	-88.4961852661	19.9843399712	Quintana Roo	MEXICO	58
Lutzomyia	*steatopyga*	Saban	-88.4961852661	19.9843399712	Quintana Roo	MEXICO	58
Lutzomyia	*trinidadensis*	Saban	-88.4961852661	19.9843399712	Quintana Roo	MEXICO	58
Brumptomyia	*mesai*	Saban	-88.4961852661	19.9843399712	Quintana Roo	MEXICO	58
Lutzomyia	*shannoni*	Saban	-88.4961852661	19.9843399712	Quintana Roo	MEXICO	58
Lutzomyia	*undulata*	Saban	-88.4961852661	19.9843399712	Quintana Roo	MEXICO	58
Lutzomyia	*cruciata*	Saban	-88.4571559523	19.9675808880	Quintana Roo	MEXICO	58
Lutzomyia	*cruciata*	Saban	-88.4961852661	19.9843399712	Quintana Roo	MEXICO	58
Lutzomyia	*shannoni*	Sam Houston State Park	-93.3388916667	30.2086277778	Louisiana	USA	87
Lutzomyia	*anthophora*	San Antonio	-98.4936277778	29.4241222222	Texas	USA	62, 104, 105
Lutzomyia	*texana*	San Antonio	-98.4936277778	29.4241222222	Texas	USA	19, 104, 105

Lutzomyia	*steatopyga*	San Antonio (Cayo)	-89.0239083333	17.0796666667	Distrito el Cayo	BELIZE	99, 100
Lutzomyia	*trinidadensis*	San Antonio (Cayo)	-89.0239083333	17.0796666667	Distrito el Cayo	BELIZE	57, 99, 100
Lutzomyia	*ovallesi*	San Antonio (Cayo)	-89.0239083333	17.0796666667	Distrito el Cayo	BELIZE	57, 99
Lutzomyia	*beltrani*	San Antonio (Cayo)	-89.0239083333	17.0796666667	Distrito el Cayo	BELIZE	57, 99
Lutzomyia	*olmeca*	San Antonio (Cayo)	-89.0239083333	17.0796666667	Distrito el Cayo	BELIZE	101, 102
Lutzomyia	*panamensis*	San Antonio (Cayo)	-89.0239083333	17.0796666667	Distrito el Cayo	BELIZE	101, 102
Lutzomyia	*shannoni*	San Antonio (Cayo)	-89.0239083333	17.0796666667	Distrito el Cayo	BELIZE	57, 99
Lutzomyia	*deleoni*	San Antonio (Cayo)	-89.0239083333	17.0796666667	Distrito el Cayo	BELIZE	57, 99
Lutzomyia	*carpenteri*	San Antonio (Cayo)	-89.0239083333	17.0796666667	Distrito el Cayo	BELIZE	99, 100
Lutzomyia	*cruciata*	San Antonio (Cayo)	-89.0239083333	17.0796666667	Distrito el Cayo	BELIZE	101, 102
Lutzomyia	*cruciata*	San Antonio Buenavista	-91.6496444444	16.1524722222	Chiapas	MEXICO	74
Lutzomyia	*beltrani*	San Antonio Nuevo	-88.0786733250	19.6262442933	Quintana Roo	MEXICO	58
Lutzomyia	*carpenteri*	San Antonio Nuevo	-88.0786733250	19.6262442933	Quintana Roo	MEXICO	58

Lutzomyia	*cratifer*	San Antonio Nuevo	-88.0786733250	19.6262442933	Quintana Roo	MEXICO	58
Lutzomyia	*deleoni*	San Antonio Nuevo	-88.0786733250	19.6262442933	Quintana Roo	MEXICO	58
Lutzomyia	*shannoni*	San Antonio Nuevo	-88.0786733250	19.6262442933	Quintana Roo	MEXICO	58
Lutzomyia	*steatopyga*	San Antonio Nuevo	-88.0786733250	19.6262442933	Quintana Roo	MEXICO	58
Lutzomyia	*trinidadensis*	San Antonio Nuevo	-88.0786733250	19.6262442933	Quintana Roo	MEXICO	58
Lutzomyia	*longipalpis*	San Antonio Nuevo	-88.0786733250	19.6262442933	Quintana Roo	MEXICO	58
Lutzomyia	*undulata*	San Antonio Nuevo	-88.0786733250	19.6262442933	Quintana Roo	MEXICO	58
Lutzomyia	*undulata*	San Antonio Nuevo	-88.0662706281	19.6239327247	Quintana Roo	MEXICO	58
Lutzomyia	*cratifer*	San Antonio Nuevo	-88.0662706281	19.6239327247	Quintana Roo	MEXICO	58
Lutzomyia	*deleoni*	San Antonio Nuevo	-88.0662706281	19.6239327247	Quintana Roo	MEXICO	58
Lutzomyia	*longipalpis*	San Antonio Nuevo	-88.0662706281	19.6239327247	Quintana Roo	MEXICO	58
Lutzomyia	*shannoni*	San Antonio Nuevo	-88.0662706281	19.6239327247	Quintana Roo	MEXICO	58
Lutzomyia	*steatopyga*	San Antonio Nuevo	-88.0662706281	19.6239327247	Quintana Roo	MEXICO	58

Lutzomyia	*cruciata*	San Antonio Nuevo	-88.0786733250	19.6262442933	Quintana Roo	MEXICO	58
Lutzomyia	*cruciata*	San Antonio Nuevo	-88.0662706281	19.6239327247	Quintana Roo	MEXICO	58
Lutzomyia	*cayennensis*	San Carlos Yautepec	-96.0006572222	16.4965500000	Oaxaca	MEXICO	38
Lutzomyia	*shannoni*	San Felasco Hammock	-82.4665972222	29.7282472222	Florida	USA	54, 55, 104, 105
Lutzomyia	*vexator*	San Felasco Hammock	-82.4665972222	29.7282472222	Florida	USA	55
Lutzomyia	*cruciata*	San Felasco Hammock	-82.4665972222	29.7282472222	Florida	USA	104, 105
Lutzomyia	*cruciata*	San Francisco	-89.9330888889	16.7917833333	Peten	GUATEMALA	105
Lutzomyia	*panamensis*	San Ignacio	-89.0752777777	17.1667476852	Distrito el Cayo	BELIZE	57
Lutzomyia	*olmeca*	San Ignacio	-89.0752777777	17.1667476852	Distrito el Cayo	BELIZE	57
Lutzomyia	*shannoni*	San Ignacio	-89.0752777777	17.1667476852	Distrito el Cayo	BELIZE	57
Lutzomyia	*carpenteri*	San Ignacio	-89.0752777777	17.1667476852	Distrito el Cayo	BELIZE	99, 100
Lutzomyia	*cruciata*	San Ignacio	-89.0752777777	17.1667476852	Distrito el Cayo	BELIZE	57
Brumptomyia	*mesai*	San Isidro Poniente	-88.8904288067	19.3252425328	Quintana Roo	MEXICO	58

Lutzomyia	*shannoni*	San Isidro Poniente	-88.8904288067	19.3252425328	Quintana Roo	MEXICO	58
Lutzomyia	*longipalpis*	San Isidro Poniente	-88.8904288067	19.3252425328	Quintana Roo	MEXICO	58
Lutzomyia	*steatopyga*	San Isidro Poniente	-88.8904288067	19.3252425328	Quintana Roo	MEXICO	58
Lutzomyia	*deleoni*	San Isidro Poniente	-88.9046755113	19.3442541584	Quintana Roo	MEXICO	58
Lutzomyia	*shannoni*	San Isidro Poniente	-88.9046755113	19.3442541584	Quintana Roo	MEXICO	58
Lutzomyia	*steatopyga*	San Isidro Poniente	-88.9046755113	19.3442541584	Quintana Roo	MEXICO	58
Lutzomyia	*cayennensis*	San Isidro Poniente	-88.9046755113	19.3442541584	Quintana Roo	MEXICO	58
Lutzomyia	*cratifer*	San Isidro Poniente	-88.9046755113	19.3442541584	Quintana Roo	MEXICO	58
Lutzomyia	*undulata*	San Isidro Poniente	-88.9046755113	19.3442541584	Quintana Roo	MEXICO	58
Lutzomyia	*cruciata*	San Isidro Poniente	-88.8904288067	19.3252425328	Quintana Roo	MEXICO	58
Lutzomyia	*cruciata*	San Isidro Poniente	-88.9046755113	19.3442541584	Quintana Roo	MEXICO	58
Lutzomyia	*cruciata*	San Jose Chinantequilla	-95.9900000000	17.3069400000	Oaxaca	MEXICO	38
Lutzomyia	*carpenteri*	San Jose Nexapa	-92.2582222222	15.0628333333	Chiapas	MEXICO	66

Lutzomyia	*cruciata*	San Jose Nexapa	-92.2582222222	15.0628333333	Chiapas	MEXICO	66
Lutzomyia	*cruciata*	San Juan Viejo	-95.1597200000	17.0444400000	Oaxaca	MEXICO	43
Lutzomyia	*longipalpis*	San Lucas	-100.7854999996	18.5702777778	Michoacan de Ocampo	MEXICO	43
Lutzomyia	*shannoni*	San Miguel Chongos	-95.9677777700	16.0050000000	Oaxaca	MEXICO	38
Lutzomyia	*cruciata*	San Miguel Chongos	-95.9677777700	16.0050000000	Oaxaca	MEXICO	38
Lutzomyia	*cruciata*	San Miguel Ecatepec	-95.7550000000	16.2597222222	Oaxaca	MEXICO	38
Lutzomyia	*cayennensis*	San Pedro Columbia	-88.9535555555	16.2701166667	Distrito Toledo	BELIZE	57
Lutzomyia	*trinidadensis*	San Pedro Columbia	-88.9535555555	16.2701166667	Distrito Toledo	BELIZE	57
Lutzomyia	*olmeca*	San Pedro Columbia	-88.9535555555	16.2701166667	Distrito Toledo	BELIZE	57
Lutzomyia	*shannoni*	San Pedro Columbia	-88.9535555555	16.2701166667	Distrito Toledo	BELIZE	57
Lutzomyia	*cruciata*	San Pedro Columbia	-88.9535555555	16.2701166667	Distrito Toledo	BELIZE	57
Lutzomyia	*cruciata*	San Pedro Ixcatlan	-96.5097222220	18.1444440000	Oaxaca	MEXICO	38
Lutzomyia	*shannoni*	San Pedro Mezcalapa	-94.3094400000	17.8936100000	Veracruz Ignacio de la Llave	MEXICO	47

Lutzomyia	*undulata*	San Pedro Mezcalapa	-96.6941527778	19.8387527778	Veracruz Ignacio de la Llave	MEXICO	51
Lutzomyia	*carpenteri*	San Pedro Peralta	-88.8498910442	18.6538842727	Quintana Roo	MEXICO	58
Lutzomyia	*deleoni*	San Pedro Peralta	-88.8498910442	18.6538842727	Quintana Roo	MEXICO	58
Brumptomyia	*mesai*	San Pedro Peralta	-88.8498910442	18.6538842727	Quintana Roo	MEXICO	58
Lutzomyia	*ovallesi*	San Pedro Peralta	-88.8498910442	18.6538842727	Quintana Roo	MEXICO	58
Lutzomyia	*panamensis*	San Pedro Peralta	-88.8498910442	18.6538842727	Quintana Roo	MEXICO	58
Lutzomyia	*shannoni*	San Pedro Peralta	-88.8498910442	18.6538842727	Quintana Roo	MEXICO	58
Lutzomyia	*steatopyga*	San Pedro Peralta	-88.8498910442	18.6538842727	Quintana Roo	MEXICO	58
Lutzomyia	*olmeca*	San Pedro Peralta	-88.8498910442	18.6538842727	Quintana Roo	MEXICO	58
Lutzomyia	*longipalpis*	San Pedro Peralta	-88.8498910442	18.6538842727	Quintana Roo	MEXICO	58
Lutzomyia	*undulata*	San Pedro Peralta	-88.8498910442	18.6538842727	Quintana Roo	MEXICO	58
Lutzomyia	*carpenteri*	San Pedro Peralta	-88.8476212070	18.6543312204	Quintana Roo	MEXICO	58
Brumptomyia	*hamata*	San Pedro Peralta	-88.8476212070	18.6543312204	Quintana Roo	MEXICO	58

Lutzomyia	*cratifer*	San Pedro Peralta	-88.8476212070	18.6543312204	Quintana Roo	MEXICO	58
Lutzomyia	*deleoni*	San Pedro Peralta	-88.8476212070	18.6543312204	Quintana Roo	MEXICO	58
Brumptomyia	*mesai*	San Pedro Peralta	-88.8476212070	18.6543312204	Quintana Roo	MEXICO	58
Lutzomyia	*longipalpis*	San Pedro Peralta	-88.8476212070	18.6543312204	Quintana Roo	MEXICO	58
Lutzomyia	*olmeca*	San Pedro Peralta	-88.8476212070	18.6543312204	Quintana Roo	MEXICO	58
Lutzomyia	*ovallesi*	San Pedro Peralta	-88.8476212070	18.6543312204	Quintana Roo	MEXICO	58
Lutzomyia	*panamensis*	San Pedro Peralta	-88.8476212070	18.6543312204	Quintana Roo	MEXICO	58
Lutzomyia	*shannoni*	San Pedro Peralta	-88.8476212070	18.6543312204	Quintana Roo	MEXICO	58
Lutzomyia	*steatopyga*	San Pedro Peralta	-88.8476212070	18.6543312204	Quintana Roo	MEXICO	58
Lutzomyia	*undulata*	San Pedro Peralta	-88.8476212070	18.6543312204	Quintana Roo	MEXICO	58
Lutzomyia	*cruciata*	San Pedro Peralta	-88.8498910442	18.6538842727	Quintana Roo	MEXICO	58
Lutzomyia	*cruciata*	San Pedro Peralta	-88.8476212070	18.6543312204	Quintana Roo	MEXICO	58
Lutzomyia	*shannoni*	San Pedro Soteapan	-94.8727777778	18.2313888888	Veracruz Ignacio de la Llave	MEXICO	51

Lutzomyia	*diabolica*	San Vicente Boqueron	-98.0539555556	18.2772249993	Puebla	MEXICO	43
Lutzomyia	*longipalpis*	San Vicente Boqueron	-98.0539555556	18.2772249993	Puebla	MEXICO	43
Lutzomyia	*diabolica*	Santa Ana	-98.7459027744	20.1153777777	Hidalgo	MEXICO	32
Lutzomyia	*cruciata*	Santa Ana	-98.7459027744	20.1153777777	Hidalgo	MEXICO	32, 105
Lutzomyia	*shannoni*	Santa Catarina Jamixtepec	-96.0411100000	16.0161100000	Oaxaca	MEXICO	47
Lutzomyia	*cruciata*	Santa Catarina Jamixtepec	-96.0411100000	16.0161100000	Oaxaca	MEXICO	38
Lutzomyia	*longipalpis*	Santa Cruz	-99.3404900000	18.3000000000	Guerrero	MEXICO	20
Lutzomyia	*shannoni*	Santa Cruz Condoy	-95.7958333333	16.9786111111	Oaxaca	MEXICO	38
Lutzomyia	*cruciata*	Santa Cruz Condoy	-95.7958333333	16.9786111111	Oaxaca	MEXICO	38
Lutzomyia	*texana*	Santa Engracia	-99.2000000000	24.0166667000	Tamaulipas	MEXICO	43
Lutzomyia	*cruciata*	Santa Engracia	-99.2000000000	24.0166667000	Tamaulipas	MEXICO	43
Brumptomyia	*mesai*	Santa Isabel	-88.0961111111	19.4700000000	Quintana Roo	MEXICO	64
Brumptomyia	*hamata*	Santa Isabel	-88.0961111111	19.4700000000	Quintana Roo	MEXICO	64

Lutzomyia	*carpenteri*	Santa Isabel	-88.0961111111	19.4700000000	Quintana Roo	MEXICO	64
Lutzomyia	*deleoni*	Santa Isabel	-88.0961111111	19.4700000000	Quintana Roo	MEXICO	64
Lutzomyia	*olmeca*	Santa Isabel	-88.0961111111	19.4700000000	Quintana Roo	MEXICO	64
Lutzomyia	*shannoni*	Santa Isabel	-88.0961111111	19.4700000000	Quintana Roo	MEXICO	64
Lutzomyia	*trinidadensis*	Santa Isabel	-88.0961111111	19.4700000000	Quintana Roo	MEXICO	64
Lutzomyia	*undulata*	Santa Isabel	-88.0961111111	19.4700000000	Quintana Roo	MEXICO	64
Lutzomyia	*cruciata*	Santa Isabel	-88.0961111111	19.4700000000	Quintana Roo	MEXICO	64
Lutzomyia	*vexator*	Santa Margarita	-120.6090583333	35.3899611111	California	USA	8
Brumptomyia	*mesai*	Ta	-93.3750000000	16.7625000000	Chiapas	MEXICO	32
Lutzomyia	*deleoni*	Santa Maria	-93.3750000000	16.7625000000	Chiapas	MEXICO	32
Lutzomyia	*olmeca*	Santa Maria	-93.8266700000	16.9186100000	Chiapas	MEXICO	40
Lutzomyia	*shannoni*	Santa Maria	-93.8266700000	16.9186100000	Chiapas	MEXICO	40
Lutzomyia	*cratifer*	Santa Maria	-93.7216670000	16.6946666667	Chiapas	MEXICO	47

Lutzomyia	*beltrani*	Santa Maria	-93.7216670000	16.6946666667	Chiapas	MEXICO	46
Lutzomyia	*deleoni*	Santa Maria	-93.7216670000	16.6946666667	Chiapas	MEXICO	46
Lutzomyia	*cruciata*	Santa Maria	-93.8266700000	16.9186100000	Chiapas	MEXICO	105
Lutzomyia	*undulata*	Santa Maria Huatulco	-93.3750000000	16.7625000000	Oaxaca	MEXICO	98
Lutzomyia	*cruciata*	Santa Maria Xadani	-96.0411111111	16.0161111111	Oaxaca	MEXICO	43
Lutzomyia	*olmeca*	Santiago Jalahui (Xalahui)	-95.7700666667	17.4526222222	Oaxaca	MEXICO	40
Lutzomyia	*cruciata*	Santiago Jalahui (Xalahui)	-95.7700666667	17.4526222222	Oaxaca	MEXICO	43
Lutzomyia	*shannoni*	Santo Domingo Tehuantepec	-95.2409166667	16.3195944444	Oaxaca	MEXICO	47
Lutzomyia	*bispinosa*	Sayab Camp	-88.9666667000	17.1000000000	Distrito el Cayo	BELIZE	53
Lutzomyia	*panamensis*	Sayab Camp	-88.9666667000	17.1000000000	Distrito el Cayo	BELIZE	53
Lutzomyia	*ylephiletor*	Sayab Camp	-88.9666667000	17.1000000000	Distrito el Cayo	BELIZE	53
Lutzomyia	*trinidadensis*	Sayab Camp	-88.9666667000	17.1000000000	Distrito el Cayo	BELIZE	53
Lutzomyia	*shannoni*	Sayab Camp	-88.9666667000	17.1000000000	Distrito el Cayo	BELIZE	53, 57, 99

Lutzomyia	*ovallesi*	Sayab Camp	-88.9666667000	17.1000000000	Distrito el Cayo	BELIZE	53
Lutzomyia	*permira*	Sayab Camp	-88.9666667000	17.1000000000	Distrito el Cayo	BELIZE	57, 99
Lutzomyia	*olmeca*	Sayab Camp	-88.9666667000	17.1000000000	Distrito el Cayo	BELIZE	57, 99
Lutzomyia	*deleoni*	Sayab Camp	-88.9666667000	17.1000000000	Distrito el Cayo	BELIZE	103
Lutzomyia	*cruciata*	Sayab Camp	-88.9666667000	17.1000000000	Distrito el Cayo	BELIZE	53
Brumptomyia	*mesai*	Seye	-89.3705444444	20.8371805556	Yucatan	MEXICO	84
Lutzomyia	*cruciata*	Seye	-89.3705444444	20.8371805556	Yucatan	MEXICO	84
Lutzomyia	*californica*	Shafter	-119.2717750000	35.5010000000	California	USA	8
Lutzomyia	*panamensis*	Sibun Camp	-88.6556777778	17.1318805556	Distrito el Cayo	BELIZE	57
Lutzomyia	*trinidadensis*	Sibun Camp	-88.6556777778	17.1318805556	Distrito el Cayo	BELIZE	57
Lutzomyia	*shannoni*	Sibun Camp	-88.6556777778	17.1318805556	Distrito el Cayo	BELIZE	57
Lutzomyia	*ovallesi*	Sibun Camp	-88.6556777778	17.1318805556	Distrito el Cayo	BELIZE	57
Lutzomyia	*bispinosa*	Sibun Camp	-88.6556777778	17.1318805556	Distrito el Cayo	BELIZE	103

Lutzomyia	*ylephiletor*	Sibun Camp	-88.6556777778	17.1318805556	Distrito el Cayo	BELIZE	103
Lutzomyia	*cruciata*	Sibun Camp	-88.6556777778	17.1318805556	Distrito el Cayo	BELIZE	57
Lutzomyia	*shannoni*	Sierra de Santa Cruz	-89.2500000000	15.6666111111	Izabal	GUATEMALA	77
Lutzomyia	*olmeca*	Sierra de Santa Cruz	-89.2500000000	15.6666111111	Izabal	GUATEMALA	77
Lutzomyia	*ylephiletor*	Sierra de Santa Cruz	-89.2500000000	15.6666111111	Izabal	GUATEMALA	77
Lutzomyia	*bispinosa*	Sierra de Santa Cruz	-89.2500000000	15.6666111111	Izabal	GUATEMALA	77
Lutzomyia	*panamensis*	Sierra de Santa Cruz	-89.2500000000	15.6666111111	Izabal	GUATEMALA	77
Lutzomyia	*cruciata*	Sierra de Santa Cruz	-89.2500000000	15.6666111111	Izabal	GUATEMALA	77
Lutzomyia	*anthophora*	Sinton	-97.5091611111	28.0367611111	Texas	USA	104, 105
Lutzomyia	*vexator*	Skalkhado	-114.1596638889	46.2482722222	Montana	USA	9
Lutzomyia	*permira*	Soccoths	-88.9962138889	17.0810277778	Distrito el Cayo	BELIZE	53, 57
Lutzomyia	*deleoni*	Solferino	-87.4062067276	21.3452542917	Quintana Roo	MEXICO	58
Brumptomyia	*mesai*	Solferino	-87.4062067276	21.3452542917	Quintana Roo	MEXICO	58

Brumptomyia	*hamata*	Solferino	-87.4062067276	21.3452542917	Quintana Roo	MEXICO	58
Lutzomyia	*olmeca*	Solferino	-87.4062067276	21.3452542917	Quintana Roo	MEXICO	58
Lutzomyia	*panamensis*	Solferino	-87.4062067276	21.3452542917	Quintana Roo	MEXICO	58
Lutzomyia	*shannoni*	Solferino	-87.4062067276	21.3452542917	Quintana Roo	MEXICO	58
Lutzomyia	*steatopyga*	Solferino	-87.4062067276	21.3452542917	Quintana Roo	MEXICO	58
Lutzomyia	*trinidadensis*	Solferino	-87.4062067276	21.3452542917	Quintana Roo	MEXICO	58
Lutzomyia	*undulata*	Solferino	-87.4062067276	21.3452542917	Quintana Roo	MEXICO	58
Brumptomyia	*mesai*	Solferino	-87.3987027189	21.3342313444	Quintana Roo	MEXICO	58
Brumptomyia	*hamata*	Solferino	-87.3987027189	21.3342313444	Quintana Roo	MEXICO	58
Lutzomyia	*cayennensis*	Solferino	-87.3987027189	21.3342313444	Quintana Roo	MEXICO	58
Lutzomyia	*deleoni*	Solferino	-87.3987027189	21.3342313444	Quintana Roo	MEXICO	58
Lutzomyia	*olmeca*	Solferino	-87.3987027189	21.3342313444	Quintana Roo	MEXICO	58
Lutzomyia	*shannoni*	Solferino	-87.3987027189	21.3342313444	Quintana Roo	MEXICO	58

Lutzomyia	*steatopyga*	Solferino	-87.3987027189	21.3342313444	Quintana Roo	MEXICO	58
Lutzomyia	*cruciata*	Solferino	-87.4062067276	21.3452542917	Quintana Roo	MEXICO	58
Lutzomyia	*cruciata*	Solferino	-87.3987027189	21.3342313444	Quintana Roo	MEXICO	58
Lutzomyia	*deleoni*	Solidaridad	-88.3052777778	18.5036111111	Quintana Roo	MEXICO	38
Lutzomyia	*olmeca*	Solidaridad	-88.3052777778	18.5036111111	Quintana Roo	MEXICO	40
Lutzomyia	*shannoni*	Solidaridad	-88.3052777778	18.5036111111	Quintana Roo	MEXICO	40
Lutzomyia	*cruciata*	Solola	-91.1833333333	14.7666250000	Solola	GUATEMALA	43
Brumptomyia	*hamata*	Sontecomapan	-95.0355600000	18.5041700000	Veracruz Ignacio de la Llave	MEXICO	22
Lutzomyia	*deleoni*	Sontecomapan	-95.0355600000	18.5041700000	Veracruz Ignacio de la Llave	MEXICO	22, 45
Lutzomyia	*panamensis*	Sontecomapan	-95.0355600000	18.5041700000	Veracruz Ignacio de la Llave	MEXICO	22
Brumptomyia	*mesai*	Sontecomapan	-95.0355600000	18.5041700000	Veracruz Ignacio de la Llave	MEXICO	38, 43
Lutzomyia	*chiapanensis*	Soyacuatla	-96.5777777778	19.5747222222	Veracruz Ignacio de la Llave	MEXICO	51
Lutzomyia	*longipalpis*	Soyacuatla	-96.5777777778	19.5747222222	Veracruz Ignacio de la Llave	MEXICO	51

Lutzomyia	*texana*	Soyacuatla	-96.5777777778	19.5747222222	Veracruz Ignacio de la Llave	MEXICO	51
Lutzomyia	*cruciata*	Soyacuatla	-96.5777777778	19.5747222222	Veracruz Ignacio de la Llave	MEXICO	51
Lutzomyia	*panamensis*	Spanish Lookout	-89.0280556000	17.3052778000	Distrito el Cayo	BELIZE	57, 99
Lutzomyia	*olmeca*	Spanish Lookout	-89.0280556000	17.3052778000	Distrito el Cayo	BELIZE	57, 99
Lutzomyia	*shannoni*	Spanish Lookout	-89.0280556000	17.3052778000	Distrito el Cayo	BELIZE	57, 99
Lutzomyia	*ovallesi*	Spanish Lookout	-89.0280556000	17.3052778000	Distrito el Cayo	BELIZE	57, 99
Lutzomyia	*cruciata*	Spanish Lookout	-89.0280556000	17.3052778000	Distrito el Cayo	BELIZE	103
Lutzomyia	*shannoni*	Spanish Water Hole	-89.0833333333	16.8999416667	Distrito el Cayo	BELIZE	57
Lutzomyia	*deleoni*	Spanish Water Hole	-89.0833333333	16.8999416667	Distrito el Cayo	BELIZE	103
Lutzomyia	*diabolica*	Stacy Ranch	-99.1013500000	28.8303111111	Texas	USA	78
Lutzomyia	*anthophora*	Stacy Ranch	-99.1013500000	28.8303111111	Texas	USA	78
Lutzomyia	*shannoni*	Stewart	-87.7763333333	36.0517500000	Tennessee	USA	67
Lutzomyia	*vexator*	Stewart	-87.7763333333	36.0517500000	Tennessee	USA	67

Lutzomyia	*vexator*	Strawberry Canyon	-122.2008302778	37.7484916667	California	USA	8, 31
Lutzomyia	*californica*	Suprestitions Mountains	-111.2392861111	33.4782027778	Arizona	USA	105
Lutzomyia	*vexator*	Sussex	-74.6076611111	41.2098166667	New Jersey	USA	76
Lutzomyia	*shannoni*	Suwanne National Wildlife Refuge	-82.9227361111	29.4784444444	Florida	USA	34
Lutzomyia	*vexator*	Suwanne National Wildlife Refuge	-82.9227361111	29.4784444444	Florida	USA	34
Lutzomyia	*cruciata*	Tamazunchale	-98.7833333000	21.2666667000	San Luis Potosi	MEXICO	43, 105
Lutzomyia	*texana*	Taniche	-96.7527777778	16.5658333333	Oaxaca	MEXICO	38
Lutzomyia	*cruciata*	Tapachula	-92.2605600000	14.9080600000	Chiapas	MEXICO	105
Lutzomyia	*olmeca*	Teapa	-92.9533250000	17.5504694444	Tabasco	MEXICO	98
Lutzomyia	*ylephiletor*	Teapa	-92.9533250000	17.5504694444	Tabasco	MEXICO	32
Lutzomyia	*cruciata*	Teapa	-92.9533250000	17.5504694444	Tabasco	MEXICO	43, 105
Lutzomyia	*cruciata*	Teotihuacan del Valle	-92.2640277778	14.9999166667	Chiapas	MEXICO	66
Lutzomyia	*serrana*	Tepozal	-105.0661100000	21.3872200000	Nayarit	MEXICO	43

Lutzomyia	*shannoni*	Tepozal	-105.0661100000	21.3872200000	Nayarit	MEXICO	47
Lutzomyia	*cruciata*	Tepozal	-105.0661100000	21.3872200000	Nayarit	MEXICO	43
Lutzomyia	*longipalpis*	Tequesquitengo	-99.2597200000	18.6111100000	Morelos	MEXICO	43
Lutzomyia	*cruciata*	Texin	-96.9961110000	19.3680560000	Veracruz Ignacio de la Llave	MEXICO	51
Lutzomyia	*shannoni*	Tishomingo State Park	-88.2317111111	34.6367611111	Mississippi	USA	39
Lutzomyia	*cruciata*	Toma de Agua	-96.6663888889	15.8872222222	Oaxaca	MEXICO	38
Lutzomyia	*deleoni*	Tomas Garrido	-89.0602698348	18.0623525942	Quintana Roo	MEXICO	70
Lutzomyia	*steatopyga*	Tomas Garrido	-89.0602698348	18.0623525942	Quintana Roo	MEXICO	70
Lutzomyia	*olmeca*	Tomas Garrido	-89.0602698348	18.0623525942	Quintana Roo	MEXICO	70
Lutzomyia	*ovallesi*	Tomas Garrido	-89.0602698348	18.0623525942	Quintana Roo	MEXICO	70
Lutzomyia	*panamensis*	Tomas Garrido	-89.0602698348	18.0623525942	Quintana Roo	MEXICO	70
Lutzomyia	*shannoni*	Tomas Garrido	-89.0602698348	18.0623525942	Quintana Roo	MEXICO	70
Lutzomyia	*cruciata*	Tomas Garrido	-89.0602698348	18.0623525942	Quintana Roo	MEXICO	70

Lutzomyia	*cruciata*	Tonaguia	-96.0325000000	17.3322222222	Oaxaca	MEXICO	40
Lutzomyia	*vexator*	Topaz Lake	-119.5397722222	38.6749527778	California	USA	8, 31
Lutzomyia	*shannoni*	Torrega State Park	-84.8567166667	30.1507444444	Florida	USA	104, 105
Lutzomyia	*cruciata*	Trece Aguas	-89.4403000000	16.1988416667	Peten	GUATEMALA	17, 57
Lutzomyia	*shannoni*	Trenton	-77.3561111111	35.0635750000	North Caroline	USA	105
Lutzomyia	*deleoni*	Tres Garantias	-87.4062067276	21.3452542917	Quintana Roo	MEXICO	58
Brumptomyia	*mesai*	Tres Garantias	-87.4062067276	21.3452542917	Quintana Roo	MEXICO	58
Lutzomyia	*olmeca*	Tres Garantias	-87.4062067276	21.3452542917	Quintana Roo	MEXICO	58
Lutzomyia	*panamensis*	Tres Garantias	-87.4062067276	21.3452542917	Quintana Roo	MEXICO	58
Lutzomyia	*shannoni*	Tres Garantias	-87.4062067276	21.3452542917	Quintana Roo	MEXICO	58
Lutzomyia	*ovallesi*	Tres Garantias	-87.4062067276	21.3452542917	Quintana Roo	MEXICO	58
Lutzomyia	*undulata*	Tres Garantias	-87.4062067276	21.3452542917	Quintana Roo	MEXICO	58
Lutzomyia	*carpenteri*	Tres Garantias	-89.0445104588	18.2138640187	Quintana Roo	MEXICO	58

Lutzomyia	*deleoni*	Tres Garantias	-89.0445104588	18.2138640187	Quintana Roo	MEXICO	58
Lutzomyia	*olmeca*	Tres Garantias	-89.0445104588	18.2138640187	Quintana Roo	MEXICO	58
Lutzomyia	*ovallesi*	Tres Garantias	-89.0445104588	18.2138640187	Quintana Roo	MEXICO	58
Lutzomyia	*panamensis*	Tres Garantias	-89.0445104588	18.2138640187	Quintana Roo	MEXICO	58
Lutzomyia	*permira*	Tres Garantias	-89.0445104588	18.2138640187	Quintana Roo	MEXICO	58
Lutzomyia	*shannoni*	Tres Garantias	-89.0445104588	18.2138640187	Quintana Roo	MEXICO	58
Lutzomyia	*steatopyga*	Tres Garantias	-89.0445104588	18.2138640187	Quintana Roo	MEXICO	58
Lutzomyia	*cruciata*	Tres Garantias	-87.4062067276	21.3452542917	Quintana Roo	MEXICO	58
Lutzomyia	*cruciata*	Tres Garantias	-89.0445104588	18.2138640187	Quintana Roo	MEXICO	58
Lutzomyia	*shannoni*	Tres Rios	-96.6911100000	15.8766700000	Oaxaca	MEXICO	47
Lutzomyia	*cruciata*	Tres Rios	-96.6911100000	15.8766700000	Oaxaca	MEXICO	38
Lutzomyia	*shannoni*	Trigg	-87.7763333333	36.8473638889	Kentucky	USA	67
Lutzomyia	*vexator*	Trigg	-87.7763333333	36.8473638889	Kentucky	USA	67

Lutzomyia	*serrana*	Tulin	-94.9563888889	18.2388888888	Veracruz Ignacio de la Llave	MEXICO	51
Lutzomyia	*shannoni*	Tulin	-94.9563888889	18.2388888888	Veracruz Ignacio de la Llave	MEXICO	51
Lutzomyia	*shannoni*	Tumbo Madedero	-90.7904930000	18.5999910000	Campeche	MEXICO	3, 4
Lutzomyia	*panamensis*	Tumbo Madedero	-90.7904930000	18.5999910000	Campeche	MEXICO	3, 4
Lutzomyia	*cruciata*	Tumbo Madedero	-90.7904930000	18.5999910000	Campeche	MEXICO	3, 4
Lutzomyia	*longipalpis*	Tuxtla Gutierrez	-93.1166666667	16.7500138889	Chiapas	MEXICO	32
Lutzomyia	*cayennensis*	Tuzales	-96.1000000000	18.7811111111	Veracruz Ignacio de la Llave	MEXICO	51
Lutzomyia	*deleoni*	Tuzik	-88.1678613955	19.9168103554	Quintana Roo	MEXICO	70
Lutzomyia	*steatopyga*	Tuzik	-88.1678613955	19.9168103554	Quintana Roo	MEXICO	70
Lutzomyia	*shannoni*	Tuzik	-88.1678613955	19.9168103554	Quintana Roo	MEXICO	70
Lutzomyia	*cruciata*	Tuzik	-88.1678613955	19.9168103554	Quintana Roo	MEXICO	70
Lutzomyia	*stewarti*	UC Hopland Field Saint	-123.1142111111	38.9706777778	California	USA	8
Lutzomyia	*vexator*	UC Hopland Field Saint	-123.1142111111	38.9706777778	California	USA	8

Lutzomyia	*cayennensis*	Uh-May	-88.0486111111	19.4166666667	Quintana Roo	MEXICO	18
Lutzomyia	*shannoni*	Uh-May	-88.0486111111	19.4166666667	Quintana Roo	MEXICO	18
Lutzomyia	*deleoni*	Uh-May	-88.0486111111	19.4166666667	Quintana Roo	MEXICO	18
Lutzomyia	*olmeca*	Uh-May	-88.0486111111	19.4166666667	Quintana Roo	MEXICO	18
Lutzomyia	*panamensis*	Uh-May	-88.0486111111	19.4166666667	Quintana Roo	MEXICO	18
Lutzomyia	*trinidadensis*	Uh-May	-88.0486111111	19.4166666667	Quintana Roo	MEXICO	18
Lutzomyia	*cruciata*	Uh-May	-88.0486111111	19.4166666667	Quintana Roo	MEXICO	18
Lutzomyia	*stewarti*	Ukiah	-123.2077833333	39.1501722222	California	USA	105
Lutzomyia	*californica*	Ukiah	-123.2077833333	39.1501722222	California	USA	8
Lutzomyia	*anthophora*	Uvalde	-99.7861666667	29.2096833333	Texas	USA	1, 104, 105
Lutzomyia	*diabolica*	Uvalde	-99.7861666667	29.2096833333	Texas	USA	1, 23, 27, 43
Lutzomyia	*shannoni*	Valentine Lake	-92.6853416667	31.2425944444	Louisiana	USA	87
Lutzomyia	*deleoni*	Veinte de Noviembre	-89.4608333300	18.0913888900	Campeche	MEXICO	83

Brumptomyia	*mesai*	Veinte de Noviembre	-89.4608333300	18.0913888900	Campeche	MEXICO	83
Lutzomyia	*olmeca*	Veinte de Noviembre	-89.4608333300	18.0913888900	Campeche	MEXICO	83
Lutzomyia	*ovallesi*	Veinte de Noviembre	-89.4608333300	18.0913888900	Campeche	MEXICO	83
Lutzomyia	*panamensis*	Veinte de Noviembre	-89.4608333300	18.0913888900	Campeche	MEXICO	83
Lutzomyia	*shannoni*	Veinte de Noviembre	-89.4608333300	18.0913888900	Campeche	MEXICO	83
Lutzomyia	*permira*	Veinte de Noviembre	-89.4608333300	18.0913888900	Campeche	MEXICO	83
Lutzomyia	*cruciata*	Veinte de Noviembre	-89.4608333300	18.0913888900	Campeche	MEXICO	83
Lutzomyia	*shannoni*	Vergel	-97.5705555550	16.8721805556	Oaxaca	MEXICO	38
Lutzomyia	*cruciata*	Vistahermosa	-96.3277800000	17.6347200000	Oaxaca	MEXICO	40
Lutzomyia	*shannoni*	Wakula Springs	-84.3051750000	30.2335361111	Florida	USA	104, 105
Lutzomyia	*vexator*	Walnut Creek	-122.0650222222	37.9062666667	California	USA	8
Lutzomyia	*shannoni*	Waycross	-82.3540166667	31.2135500000	Georgia	USA	105
Lutzomyia	*texana*	Welder Wildlife Refuge	-97.7719416667	27.9543305556	Texas	USA	104, 105

Lutzomyia	*californica*	Wendel	-120.2335472222	40.3482361111	California	USA	8
Lutzomyia	*shannoni*	West Feliciana	-91.4048250000	30.8433361111	Louisiana	USA	87
Lutzomyia	*shannoni*	Wilmington	-75.5466666667	39.7456027778	Delaware	USA	105
Lutzomyia	*vexator*	Woodside	-122.2537972222	37.4296055556	California	USA	8
Lutzomyia	*longipalpis*	Zacapa	-89.5333333333	14.9666944444	Zacapa	GUATEMALA	57
Lutzomyia	*cruciata*	Zamora Caleton	-95.4830555556	18.5797222220	Veracruz Ignacio de la Llave	MEXICO	38
Lutzomyia	*beltrani*	Zapotal	-94.3036100000	17.8583300000	Veracruz Ignacio de la Llave	MEXICO	46
Lutzomyia	*olmeca*	Zapotal	-94.3036100000	17.8583300000	Veracruz Ignacio de la Llave	MEXICO	38, 47
Lutzomyia	*shannoni*	Zapotal	-94.3036100000	17.8583300000	Veracruz Ignacio de la Llave	MEXICO	38
Lutzomyia	*cruciata*	Zongolica	-96.9986111111	18.6647222222	Veracruz Ignacio de la Llave	MEXICO	38
Lutzomyia	*cayennensis*	Zumpango del Rio	-98.6838305556	17.9324611111	Guerrero	MEXICO	28

REFERENCES

1. Addis, C. 1945. *Phlebotomus* (*Dampfomyia*) *anthophorus,* n.sp., and *Phlebotomus diabolicus* Hall from Texas (Diptera: Psychodidae). *The Journal of Parasitology.* **31:** 119-127.

2. Aitken, T., AJ. Main and D. Young. 1977. *Lutzomyia vexator* (Coquillet) in Connecticut (Diptera: Psychodidae). *Proceedings of the Entomological Society of Washington.* **79:** 582-582.

3. Biagi, F. and A. de Buen de Biagi. 1953a. Datos ecológicos de algunos flebotomus mexicanos (Diptera: Psych.). *Annales del Instituto de Biología.* Tomo 24. No. 2. México.

4. Biagi, F. and A. de Buen de Biagi. 1953b. Algunos flebotomus del área endêmica de Leishmaniasis tegumentária Americana del Estado de Campeche, México. *Revista de Medicina.* Tomo 33. No. 679. pp 315-319. México.

5. Biagi, F., A. de Biagi and F. Beltrán. 1965. *Phlebotomus flaviscutellatus*, transmisor natural de Leishmania mexicana. *Prensa Médica México.* **30:** 267-272.

6. Biagi,F., A. de Biagi, and F. Beltrán. 1966. Actividad horaria de *Phlebotomus* antropófilos en la Península de Yucatán. *Revista de Salud Pública (México)*. **26**: 73-77.

7. Brinson, F., D. Hagan, J. Comer and D. Strohlein. 1992. Seasonal Abundance of *Lutzomyia shannoni* (Diptera: Psychodidae) on Ossabaw Island, Georgia. *Journal of Medical Entomology.* **29:** 178-182.

8. Chaniotis, B. N. & Anderson, J. R. 1968. Age structure, population dynamics and vector potential of Phlebotomus in northern California. Part II. Field population dynamics and natural flagellate infections in parous females. *Journal of Medical Entomology.* **5:** 273-292.

9. Chaniotis, B. 1974. Phlebotomine sandflies in Montana: First report. *Mosquito News.* **34:** 334-335.

10. Claborn, D., E. Rowton, P. Lawyer, G. Brown and L. Keep. 2009. Species diversity and relative abundance of Phlebotomine sand flies (Diptera: Psychodidae) on three army installations in the Southern United States and susceptibility of a domestic sand fly to infection with Old World *Leishmania major. Military Medicine.* **174:** 1203-1208.

11.Claborn, D., P. Masuoka, M. Morroz and L. Keep. 2008. Habitat analysis of North American sand flies near veterans returning from leishmania-endemic war zones. *International Journal of Health Geographics.* **7:** 65 doi:10.1186/1476-072X-7-65.

12.Comer, J., D. Kavanaugh, D. Stallknecht and J. Corn. 1994a. Population dynamics of *Lutzomyia shannoni* (Diptera: Psychodidae) in relation to the epizootiology of vesicular stomatitis virus on Ossabaw Island, Georgia. *Journal of Medical Entomology.* **31:** 850-854.

13. Comer, J., D. Kavanaugh, D. Stallknecht, G. Ware, J. Corn and V. Nettles. 1993. Effect of forest type on the distribution of *Lutzomyia shannoni* (Diptera: Psychodidae) and vesicular stomatitis virus on Ossabaw Island, Georgia. *Journal of Medical Entomology.* **30:** 555-560.

14. Comer, J. and J. Corn. 1991. Funnel trap fot the capture of Phlebotomine Sand flies (Diptera: Psychodidae) from Tree Holes. *Journal of Medical Entomology.* **28:** 289-292.

15. Comer, J. J. Corn, D. Stallknecht, J. Landgraf and V. Nettles. 1992. Titers of Vesicular Stomatitis virus, New Jersey Serotype, in naturally infected male and female *Lutzomyia shannoni* (Diptera: Psychodidae) in Georgia. *Journal of Medical Entomology.* **29:** 368-370.

16. Comer, J. W. Irby and D. Kavanaugh. 1994b. Hosts of *Lutzomyia shannoni* (Diptera: Psychodidae) in relation to vesicular stomatitis virus on Ossabaw Island, Gerogia, U.S.A. *Medical and Veterinary Entomology.* **8:** 325-330.

17. Coquillett, D. 1907. Discovery of blood-sucking Psychodidae in America. *Entomological News.* **18:** 101-102.

18. Cruz, A., J. García, P. Manrique-Saide and J. Pérez. 1994. Taxonomical identification of anthropophilic species of *Lutzomyia* in Quintana Roo, Yucatan Peninsula, Mexico. *Biomédica* . **5:** 127-131.

19. Dampf, A. 1938. Un nuevo *Phlebotomus* (Insecta, Diptera, Fam. Psychodidae) procedente de Texas, E.U.A. *Anales de la Escuela Nacional de Ciencias Biologicas.* **1:** 1-8.

20. de Buen de Biagi, A. F. Beltrán and F. Biagi. 1966. Nuevos conocimientos sobre los flebótomos del área endémica de Leishmaniasis cutánea en Yucatán. *Revista de Investigación en Salud Pública.* **26**: 139-153.

21. de Buen, A. 1966. Clave para identificación rápida de las hembras de *Phlebotomus* antropófilos del área endémica de Leishmaniasis cutánea en México. *Revista de Investigación en Salud Pública.* **26**: 367-373.

22. Díaz-Nájera, A. 1963. Lista de mosquitos capturados en tres localidades del estado de Veracruz, Mexico. *Revista del Instituto de Salubridad y Enfermedades Tropicales.* 23: 187-192.

23. Díaz-Nájera, A. 1970. Presencia de *Lutzomyia (Lutzomyia) diabolica* (Hall, 1936) en Muzquiz, Coahuila, México (Diptera: Psychodidae). *Revista de Investigación en Salud Pública.* **31:** 62-66.

24. Downes, J. 1972. Canadian records of *Phlebotomus vexator*, *Trichomyia nuda*, and *Maruina lanceolata* (Diptera: Psychodidae). *The Canadian Entomologist.* **104:** 1135-1136.

25. Eads, R., H. Treviño and E. Campos. 1965. Additional Records of *Phlebotomus texanus* . *Proceedings of the Entomological Society of Washington.* **67:** 251-252.

26. Easton, E., M. Price and O. Graham. 1968. The collection of biting flies in West Texas with Malaise and Animal-baited Traps. *Mosquito News.* **28:** 465-469.

27. Endris, R., PV. Perkins, D. Young and R. Johnson. 1982. Techniques for laboratory rearing of sand flies (Diptera: Psychodidae). *Mosquito News.* **42:** 400-407.

28. Fairchild, G. and M. Hertig. 1948. Notes on the *Phlebotomus* of Panama (Diptera: Psychodidae) IV. *P. atroclavatus* Knab, *P. cayennensis* Floch and Abonnenc, *P. chiapanensis* Dampf and some related forms from the West Indies and Mexico. *Annals of the Entomological Society of America.* **41**: 455-467.

29. Fairchild, G. and M. Hertig. 1950. Notes on the Phlebotomus of Panama (Diptera, Psychodidae). VI. *Phlebotomus shannoni* Dyar and related species. *Annals of the Entomological Society of America.* 43: 523-533.

30. Fairchild, G. B. and M. Hertig. 1956. Notes on the Phlebotomus of Panama (Diptera: Psychodidae) XII. The group anthophorus, with descriptions of four species from Panama and Mexico. *Annals of the Entomological Society of America.* **49**: 307-312.

31. Fairchild, G. B. and M. Hertig. 1957. Notes on the Phlebotomus of panamá, XIII. The vexator group, with descriptions of new species from Panamá and california**.** *Annals of the Entomological Society of America* **. 50:** 325-334.

32. Fairchild, G. B. and M. Hertig. 1959. Geographic distribution of the phlebotomus sandflies of Central America (Diptera: Psychodidae). *Annals of the Entomological Society of America* . **52**: 121-124.

33. Fairchild, G.B. and R.F. Harwood. 1961. *Phlebotomus* sandflies from animal burrows in Eastern Washington. *Proceedings of the Entomological Society of Washington.* **63:** 239-245.

34. Florin, D., SJ. Davis, C. Olsen, P. Lawyer, R. Lipnick, G. Schultz, E. Rowton, R. Wilkerson and L. Keep. 2011. Morphometric and Molecular analyses of the Sand Fly Species *Lutzomyia shannoni* (Diptera: Psychodidae: Phlebotominae) collected from seven different geographical areas in the Southeastern United States. *Journal of Medical Entomology.* **48:** 154-166.

35. Florin, D., P. Lawyer, E. Rowton, G. Schultz, R. Wilkerson, S. Davies, R. Lipnicj and L. Keep. 2010. Morphological anomalies in two *Lutzomyia (Psathyromyia) shannoni* (Diptera: Psychodidae) specimens collected from Fort Rucker, Alabama, and Fort Campbell, Kentucky. *Journal of Medical Entomology.* **47:** 952-956.

36. Galliard, H. 1934a. Un phlebotome nouveau du Mexique: *Phlebotomus almazani* n. sp. *Annales de Parasitologie Humaine et Comparée.* **12**: 193-195.

37. Galliard, H. 1934b. Un phlebotome nouveau du Mexique: *Phlebotomus yucatanensis* n. sp. *Annales de Parasitologie Humaine et Comparée.* **12**: 177-181.

38. Godínez-Alvarez, A. and S. Ibáñez-Bernal. 2010. Catalogo de Psychodidae (Diptera) de la colección de artropodos con importancia médica del INDRE, Secretaria de Salud, México. *Acta Zoologica Mexicana.* **26:** 99-121.

39. Goddard, J. and C. McHugh. 2005. New records for the Phlebotomine sand fly *Lutzomyia shannoni* (Dyar) (Diptera: Psychodidae) in Mississipi. *Journal of the Mississippi Academy of Sciences.* **50:** 195-196.

40. Gonzalez, C. Personal collections records.

41. Haddow, A., G. Curler and J. Moulton. 2008. New records of *Lutzomyia shannoni* and *Lutzomyia vexator* (Diptera: Psychodidae) in eastern Tennessee. *Journal of Vector Ecology.* **33:** 393-396.

42. Hall, D. 1936. *Phlebotomus (Brumptomyia) diabolicus,* a new species of biting gnat from Texas (Diptera: Psychodidae). *Proceedings of the Entomological Society of Washington.* **38:** 27-28.

43. Ibáñez-Bernal, S. 1999. Phlebotominae (Diptera: Psychodidae) de México. I.- *Brumptomyia* França y Parrot; *Lutzomyia* França, las especies de *Lutzomyia* (*Lutzomyia*) França y del grupo *verrucarum*. *Folia Entomológica Mexicana.* **107**: 61-116.

44. Ibañez-Bernal, S. 2000. Psychodidae (Diptera). In Llorente, J., E. González y N. Papavero. (Eds) pp 607-626. Biodiversidad, taxonomia y biogeografia de artrópodos de Mexico: Hacia una sintesis de su conocimiento. Vol II. Mexico, D.F. Universidad Nacional Autonoma de México. ISBN: 968-36-8003-8.

45. Ibáñez-Bernal, S. 2001. Phlebotominae (Diptera: Psychodidae) de México II.- Las especies de *Lutzomyia* (*Coromyia*) Barreto, del grupo *Delpozoi* y de *Lutzomyia* (*Dampfomyia*) Addis. *Folia Entomológica Mexicana.* **40**: 17-43.

46. Ibáñez-Bernal, S. 2001. Notes on the Psychodidae (Diptera) of Belize: Subfamilies Bruchomyiinae and Phlebotominae. *Annales of the Entomological Society of America.* **94:** 367-385.

47. Ibáñez-Bernal, S. 2002. Phlebotominae (Diptera: Psychodidae) de México. III. Las especies de *Lutzomyia* (*Psathyromyia*) Barreto, del grupo *Aragoi*, de *L.* (*Trichopygomyia*) Barreto, del grupo *Dreisbachi* y de *L.* (*Nyssomyia*) Barreto. *Folia Entomológica Mexicana.* **41**: 149-183.

48. Ibáñez-Bernal, S. 2003. Phlebotominae (Diptera: Psychodidae) de México. IV. Las especies de *Lutzomyia* (*Psychodopygus*) Mangabeira, *L.* (*Micropygomyia*) Barretto, *Lutzomyia* grupo *Oswaldoi, L.* (*Helcocyrtomyia*) Barreto y especies del género sin agrupar. *Folia Entomológica Mexicana.* **42:** 109-152.

49. Ibáñez-Bernal, S., G. Rodríguez, C. Gómez and J. Ricardez. 2004. First record of *Lutzomyia evansi* (Nuñez-Tovar, 1924) in Mexico (Diptera: Psychodidae, Phlebotominae). *Memorias do Instituto Oswaldo Cruz.* **99**: 127-129.

50. Ibáñez-Bernal, S., R. Hernández and F. Mendoza. 2006. Collections of Bruchomyiinae and Phlebotominae (Diptera: Psychodidae) from the north-central portion of the state of Veracruz, Mexico, with the description of a new species. *Zootaxa* . **1270**: 19-33.

51. Ibáñez-Bernal, S., T. Suárez and F. Mendoza. 2011. An updated checklist of the phlebotomine sand flies of Veracruz, Mexico (Diptera: Psychodidae, Phlebotominae). *Zootaxa.* **2928:** 29-40.

52. Lawyer, P., D. Young, J. Butler and D. Akin. 1987. Development of *Leishmania mexicana* in *Lutzomyia diabolica and Lutzomyia shannoni* (Diptera: Psychodidae). *Journal of Medical Entomology.* **24:** 347-355.

53. Lewis, D. and P. Garnham. 1959. The species of *Phlebotomus* (Diptera: Psychodidae) in British Honduras. *Proceedings of the Royal Society B.* **28:** 79-89.

54. Mann, R. and P. Kaufman. 2010a. Colonization of *Lutzomyia shannoni* (Diptera: Psychodidae) utilizing an artificial blood feeding technique. *Journal of Vector Ecology.* **35:** 286-294.

55. Mann, R. and P. Kaufman. 2010b. The seasonal abundance of phlebotomine sand flies, *Lutzomyia* species in Florida. *Journal of the American Mosquito Control Association.* **26:** 10-17.

56. Mangabeira, O. and P. Galindo. 1944. The genus *flebotomus* in California. *American Journal of Hygiene.* **49:** 182-198.

57. Martins, A., P. Williams and A. Lima. 1978. American Sand Flies (Diptera: Psychodidae, Phlebotominae). *Academia Brasileira de Ciencias.* Rio de Janeiro, Brasil. pp.197.

58. May, E., H. Hernandez and E. Rebollar-Téllez. 2011. Distribución de flebotomineos (Diptera: Psychodidae) en Quintana Roo, México. *Acta Zoológica Mexicana.* 27: 273-289.

59. McHugh, C. 1991. Distributional records for some North American Sand Flies, *Lutzomyia* (Diptera: Psychodidae). *Entomological News.* **102:** 192-194.

60. McHugh, C. 1999. Notes on the sand flies (Diptera: Psychodidae) of Southern Arizona. *Entomological News.* **110:** 297-301.

61. McHugh, C., B. Ostrander, R. Raymond and S. Kerr. 2001. Population dynamics of sand flies (Diptera: Psychodidae) at two foci of Leishmaniasis in Texas. *Journal of Medical Entomology.* **38:** 268-277.

62. McHugh, C., M. Grogl and R. Kreutzer. 1993. Isolation of *Leishmania mexicana* (Kinetoplastida: Trypanosomatidae) from *Lutzomyia anthophora* (Diptera: Psychodidae) collected in Texas. *Journal of Medical Entomology.* **30:** 631-633.

63. Mead, D. and C. Cupp. 1995. Ocurrence of *Lutzomyia anthophora* (Diptera: Psychodidae) in Arizona. *Journal of Medical and Entomology.* **32:** 747-748.

64. Méndez, C. 2005. Diversidad y Abundancia de flebótomos adultos (Diptera: Psychodidae) en tres localidades de la Península de Yucatán. Memoria de Residencia Profesional. Instituto Tecnológico Agropecuario No. 2. (ITA)-SEP-SEIT. Yucatán, México.

65. Mézquita, E. 2005. Especies de flebótomos (Diptera: Psychodidae) en dos localidades de Calakmul, Campeche con transmisión de Leishmaniasis abarcando 2002-2004. Memoria de Residencia Profesional, Instituto Tecnológico Agropecuario No. 2. (ITA)-SEP-SEIT. Yucatán, México.

66. Mikery-Pacheco, O. 2011. Detección de *Wolbachia* y *Leishmania* en poblaciones silvestres de *Lutzomyia spp.* (Diptera: Psychodidae) en la zona cafetalera de la Región Soconusco, Chiapas. Thesis. Licenciatura en Ingeniero Biotecnologo. Universidad Autonoma de Chiapas. Tapachula, Chiapas.

67. Minter, L., B. Kovacic, D. Claborn, P. Lawyer, D. Florin and G. Brown. 2009. New State records for *Lutzomyia shannoni* and *Lutzomyia vexator. Journal of Medical Entomology.* **46:** 965-968.

68. Minter, L., G. Brown and D. Johnson. 2011. Investigation of habitat effects on the spatial distribution of *Lu. shannoni* across heterogeneous environments, with note of respective mosquito species composition. *Journal of Medical Entomology.* **48:** 1103-1111.

69. Montes de Oca-Aguilar, A. 2010. Análisis prospectivo para la caracterización de sitios de desarrollo larval y de reposo de flebotomineos adultos en la Península de Yucatán. Thesis. Licenciatura en Biología. Instituto Tecnológico de Conkal, Conkal, Yucatán.

70. Moo-Llanes, D. 2009. Diversidad y distribución Geográfica de las especies de los géneros *Brumptomyia* (Franca & Parrot) y *Lutzomyia* (Franca) (Diptera: Psychodidae) en Quintana Roo, México. Thesis. Licenciatura en Biología. Universidad Autónoma de Yucatán. Yucatán, México.

71. Ortega. M. 1966. Flebotomos de la Cuenca del Balsas (Diptera: Psychodidae). Thesis. Licenciatura en Biologia. Universidad Autónoma de México. México, D.F.

72. Ostfeld, R., P. Roy, W. Haumaier, L. Canter, F. Keesing and E. Rowton. 2004. Sand fly (*Lutzomyia vexator*) (Diptera: Psychodidae) populations in upstate New York: Abundance, Microhabitat and Phenology. *Journal of Medical Entomology.* **41:** 774-778.

73. Pech-May, A., F. Escobedo, M. Berzunza and E. Rebollar. 2010. Incrimination of four sandfly previously unrecognized as vectors of Leishmania parasites in Mexico. *Medical and Veterinary Entomology.* **24:** 150-161.

74. Pech-May, A., C. Marina-Fernández, E. Vázquez-Domínguez, M. Berzunza-Cruz, E.A. Rebollar-Téllez, J.A. Narváez-Zapata, D. Moo-Llanes, S. Ibáñez-Bernal, J. Ramsey-Willoquet, and I. Becker-Fauser. 2013. Genetic structure and genetic divergence in population of *Lutzomyia cruciata,* a phlebotomine sand fly (Diptera: Psychodidae) vector of *Leishmania mexicana* in southeastern Mexico. *Infection, Genetics and Evolution.* 16: 254-262.

75. Peraza, G. 2009. Especies de flebotomineos (Diptera: Psychodidae) de importancia médica y su potencial como vectores de Leishmania mexicana (Kinetoplastida: Trypanosomatidae) en Calakmul, Campeche. Thesis. Licenciatura en Biología. Universidad Autónoma de Yucatán, México.

76. Price, D., D. Gunther and R. Gaugler. 2011. First collection records of Phlebotomine sand flies (Diptera: Pychodidae) from New Jersey. *Journal of Medical Entomology.* **48:** 476-478.

77. Porter, C., F. Steurer and R. Kreutzer. 1987. Isolation of *Leishmania mexicana mexicana* from *Lutzomyia ylephiletor* in Guatemala. *Transactions of the Royal Society of Tropical Medicine and Hygiene.* **81:** 929-930.

78. Rebollar-Téllez, E. Personal collections records.

79. Rebollar-Téllez, E., A. Ramírez and F. Andrade. 1996a. A two years study on vectors of cutaneous leishmaniasis. Evidence for sylvatic transmission cycle in the state of Campeche, Mexico. *Memorias do Instituto Oswaldo Cruz.* 91: 555-560.

80. Rebollar-Téllez, E., E. Tun, P. Manrique-Saide and F. Andrade. 2005. Relative abundance of sandfly species (Diptera: Phlebotomidae) in two villages in same area of Campeche in Southern Mexico. *Annals of Tropical Medicine and Parasitology.* 99: 193-201.

81. Rebollar-Téllez, E., F. Reyes, I. Fernández and F. Andrade. 1996b. Population dynamics and biting rhythm of the anthropophilic sandfly *Lutzomyia cruciata* (Diptera: Psychodidae) in Southeast, Mexico. *Revista do Instituto de Medicina Tropical de São Paulo* 38: 29-33.

82. Rebollar-Téllez, E., F. Reyes, I. Fernández and F. Andrade. 1996c. Abundance and parity rate of *Lutzomyia cruciata* (Diptera: Psychodidae) in an endemic focus of Localized Cutaneous Leishmaniasis in Southern Mexico. *Journal of Medical Entomology* . 33: 683-685.

83. Rebollar- Téllez, E., P. Manrique-Saide, E. Tun, A. Che and F. Dzul. 2004. Further records of phlebotomid sandflies (Diptera: Psychodidae) from Campeche, Mexico. *Entomological News.* 115: 283-291.

84. Rebollar, E.A., H. Orilla, F. Dzul, A. Che, P. Manrique and A. Zapata. 2006. An udpate on the phlebotomid sand fly (Diptera: Phlebotomidae) fauna of Yucatan, Mexico. *Entomological News.* 117: 21-23.

85. Rowton, E., M. de Mata, N. Rizzo, C. Porter and T. Navin. 1992. Isolation of *Leishmania braziliensis* from *Lutzomyia ovallesi* (Diptera: Psychodidae) in Guatemala. *American Journal of Tropical Medicine and Hygiene.* **46:** 465-468.

86. Rowton, E., M. de Mata, N. Rizzo, T. Navin and Porter, C. 1991. Vectors of *Leishmania braziliensis* in the Peten, Guatemala. *Parassitologia* **33:** (Supplement 1) 501-504.

87. Rosabal, R. and A. Miller. 1970. Phlebotomine sand flies in Louisiana (Diptera: Psychodidae). *Mosquito News.* **30:** 180-187.

88. Rozeboom, L. and C. Courson. 1944. *Phlebotomus limai* Fonseca in the United States (Diptera: Psychodidae). *The Journal of Parasitology.* **30:** 274-275.

89. Sánchez, L., C. Pozo and E. Rebollar-Téllez. 2007. “Colección de referencia de vectores de Leishmania spp. (causante de la Leishmaniasis cutánea localizada) de la Península de Yucatán y un estudio preliminar en un área endémica de Quintana Roo”. Informe Final de Actividades para la Comisión Nacional para el Conocimiento y Uso de la Biodiversidad (CONABIO).

90. Sánchez, L., M. Berzunza, I. Becker and E. Rebollar-Téllez. 2010. Sand flies naturally infected by *Leishmania (L.) mexicana* in the peri-urban area of Chetumal city, Quintana Roo, México. *Transactions of the Royal Society of Tropical Medicine and Hygiene.* **104:** 406-411.

91. Schemanchuk, J., R. Robertson and K. Depner. 1978. Ocurrence of two species of *Phlebotomus* sandflies (Diptera: Psychodidae) in burrows oy yellow-bellied marmot, *Marmota flaviventris Nosophora,* in Southern Alberta. *The Canadian Enmologist.* **110:** 1355-1358.

92. Snow, W.E. 1955. Feeding activities of some blood-sucking Diptera with reference to vertical distribution in bottomland forest. *Annals Entomological Society of America.* **48:** 512-521.

93. Treviño, N. 2001. Dinámica de infección de Leishmaniasis y fluctuación poblacional de Luztomyia spp. (Diptera: Psychodidae) en cafetales de Nayarit, México. Thesis de Maestría. Universidad Autónoma de Nuevo León.

94. Vargas, L. and A. Diáz-Nájera. 1951. *Phlebotomus betrani* n. sp. y *Phlebotomus wirthi* n. sp. del estado de Veracruz (Diptera: Psychodidae). *Revista del Instituto de Salubridad y Enfermedades Tropicales.* **12**: 101-106.

95. Vargas, L. and A. Diáz-Nájera. 1952. Dos flebotomus nuevos de Mexico (Diptera: Psychodidae). *Revista de la Sociedad Mexicana de Historia Natural.* **13**: 63-74.

96. Vargas, L. and A. Diáz-Nájera. 1953a. Lista de flebotomos mexicanos y su distribución geográfica (Diptera: Psychodidae). *Revista del Instituto de Salubridad y Enfermedades Tropicales.* **13**: 309-314.

97. Vargas, L. and A. Diáz-Nájera. 1953b. Nuevas especies de flebotomos de México (Diptera: Psychodidae). *Revista del Instituto de Salubridad y Enfermedades Tropicales.* **13**: 41-52.

98. Vargas, L. and A. Diáz-Nájera 1959. *Phlebotomus farilli* n. sp., *Ph. humboldti* n. sp., y *Ph. olmecus* n. sp. de Mexico (Diptera: Psychodidae). *Revista del Instituto de Salubridad y Enfermedades Tropicales.* **19**: 141-143.

99. Williams, P. 1970a. Phlebotomine sandflies and Leishmaniasis in British Honduras (Belize). *Royal Society of Tropical Medicine and Hygiene.* **64:** 317-363.

100. Williams, P. 1970b. On the vertical distribution of Phlebotomine sandflies (Diptera: Psychodidae) in British Honduras (Belize). *Bulletin of Entomological Research.* **59:** 637-646.

101. Williams, P. 1976. The phlebotomine sandflies (Diptera: Psychodidae) of caves in Belize, Central America. *Bulletin of Entomological Research.* **65:** 601-614.

102. **Williams, P**. **1976**. The form of *Lutzomyia beltrani* (vagas & Diáz-Nájera) (Diptera, Psychodidae) in Belize, Central America. *Bulletin of Entomological Research.* **65:** 595-599.

103. Williams, P., D. Lewis and P. Garnham. 1965. On Dermal Leishmaniasis in British Honduras. *Transactions of the Royal Society of Tropical Medicine and Hygiene.* **59:** 64-71.

104. Young, D. 1972. Phlebotomine sand flies from Texas and Florida (Diptera: Psychodidae). *The Florida Entomologist.* **55:** 61-64.

105. Young, D. G. and P.V. Perkins. 1984. Phlebotomine sand flies of North America (Diptera: Psychodidae). *Mosquito News.* **44**: 263-304.
