# Supplementary material for: Current and Future Niche of North and Central American Sand Flies (Diptera: Psychodidae) in Climate Change Scenarios
Source: PLoS Negl Trop Dis. 2013 Sep 19;7(9):e2421. doi: 10.1371/journal.pntd.0002421 (PMC3777871; doi:10.1371/journal.pntd.0002421)
Supplement: Table S2 — Test accuracy, AUC (ROC curve) and statistical significance. (PDF) [file pntd.0002421.s028.pdf]

**Table S2. Test accuracy, AUC (ROC curve) and statistical significance.**

| Species | INTERNAL TEST | | | EXTERNAL TEST | | |
|---|---|---|---|---|---|---|
| | Accuracy | AUC | *P* | Accuracy | AUC | *P* |
| *Brumptomyia hamata* | 1.00 | 0.98 | $1.10^{E-63}$ | 1.00 | 0.98 | $7.85^{E-56}$ |
| *Br. mesai* | 1.00 | 0.98 | 0.00 | 1.00 | 0.98 | $7.55^{E-136}$ |
| *Lutzomyia beltrani* | 1.00 | 0.98 | $1.61^{E-88}$ | 1.00 | 0.98 | $6.19^{E-38}$ |
| *Lu. bispinosa* | 1.00 | 0.99 | $2.35^{E-63}$ | 1.00 | 0.99 | $2.99^{E-22}$ |
| *Lu. carpenteri* | 1.00 | 0.98 | $6.86^{E-271}$ | 1.00 | 0.98 | $8.53^{E-100}$ |
| *Lu. cayennensis* | 1.00 | 0.98 | $1.52^{E-306}$ | 0.93 | 0.98 | $3.71^{E-85}$ |
| *Lu. chiapanensis* | 1.00 | 0.97 | $3.39^{E-76}$ | 0.75 | 0.96 | $4.06^{E-18}$ |
| *Lu. cratifer* | 1.00 | 0.98 | $4.09^{E-174}$ | 1.00 | 0.98 | $6.75^{E-69}$ |
| *Lu. cruciata* | 1.00 | 0.97 | 0.00 | 0.97 | 0.96 | $1.49^{E-183}$ |
| *Lu. deleoni* | 0.99 | 0.98 | 0.00 | 1.00 | 0.98 | $2.04^{E-217}$ |
| *Lu. dodgei* | 1.00 | 0.98 | $2.57^{E-55}$ | 1.00 | 0.98 | $8.51^{E-22}$ |
| *Lu. longipalpis* | 1.00 | 0.97 | $1.86^{E-190}$ | 1.00 | 0.97 | $9.61^{E-67}$ |
| *Lu. olmeca olmeca* | 0.99 | 0.98 | 0.00 | 0.96 | 0.98 | $1.70^{E-261}$ |
| *Lu. ovallesi* | 1.00 | 0.98 | 0.00 | 1.00 | 0.98 | $2.47^{E-112}$ |
| *Lu. panamensis* | 0.98 | 0.98 | 0.00 | 1.00 | 0.98 | $2.17^{E-138}$ |
| *Lu. permira* | 1.00 | 0.98 | $7.82^{E-141}$ | 1.00 | 0.98 | $6.37^{E-51}$ |
| *Lu. serrana* | 1.00 | 0.80 | $5.28^{E-93}$ | 0.80 | 0.97 | $9.82^{E-26}$ |
| *Lu. shannoni* | 0.97 | 0.92 | $3.60^{E-257}$ | 0.91 | 0.90 | $1.11^{E-73}$ |
| *Lu. steatopyga* | 1.00 | 0.98 | 0.00 | 1.00 | 0.98 | $1.88^{E-177}$ |
| *Lu. trinidadensis* | 1.00 | 0.98 | $7.87^{E-298}$ | 1.00 | 0.98 | $1.23^{E-100}$ |
| *Lu. undulata* | 1.00 | 0.98 | $2.98^{E-224}$ | 1.00 | 0.98 | $2.29^{E-81}$ |
| *Lu. ylephiletor* | 1.00 | 0.98 | $1.69^{E-77}$ | 1.00 | 0.98 | $2.52^{E-33}$ |
| *Lu. anthophora* | 0.95 | 0.97 | $5.69^{E-29}$ | 0.57 | 0.80 | $2.11^{E-04}$ |
| *Lu. californica* | 0.92 | 0.92 | $5.03^{E-16}$ | 1.00 | 0.92 | $7.04^{E-09}$ |
| *Lu. diabolica* | 1.00 | 0.93 | $2.59^{E-53}$ | 0.91 | 0.89 | $1.79^{E-15}$ |
| *Lu. stewarti* | 1.00 | 0.94 | $9.45^{E-22}$ | 1.00 | 0.95 | $7.53^{E-09}$ |
| *Lu. texana* | 0.95 | 0.94 | $5.35^{E-48}$ | 1.00 | 0.95 | $7.75^{E-19}$ |
| *Lu. vexator* | 0.83 | 0.85 | $1.68^{E-12}$ | 1.00 | 0.90 | $6.86^{E-08}$ |
