# Supplementary material for: Current and Future Niche of North and Central American Sand Flies (Diptera: Psychodidae) in Climate Change Scenarios
Source: PLoS Negl Trop Dis. 2013 Sep 19;7(9):e2421. doi: 10.1371/journal.pntd.0002421 (PMC3777871; doi:10.1371/journal.pntd.0002421)
Supplement: Table S3 — Proportion of sand fly species' ENM overlap and their territorial projection of change to 2050. (PDF) [file pntd.0002421.s029.pdf]

Table S3. Proportion of conserved sand fly species´ ENM (overlap) and overall geographic projection change between present and 2050, in both CC scenarios (%).

Categories	Species	Overlap A2	Overlap B2	ENM change in A2 (%)	ENM change in B2 (%)
Tropical	*Brumptomyia hamata*	84.33	98.02	12.91	31.18
	Br. mesai	94.33	99.39	25.89	32.06
	Lutzomyia beltrani	97.94	99.74	24.88	28.96
	Lu. bispinosa	92.10	99.29	76.68	81.01
	Lu. carpenteri	95.07	99.44	26.19	32.29
	Lu. cayennensis	97.10	99.46	25.75	28.60
	Lu. chiapanensis	97.71	99.54	18.80	23.39
	Lu. cratifer	87.86	99.07	14.05	27.61
	Lu. cruciata	97.16	97.86	71.13	62.49
	Lu. deleoni	88.13	88.13	0.00	26.57
	Lu. dodgei	88.41	98.38	14.95	26.27
	Lu. longipalpis	95.91	99.31	16.61	19.37
	Lu. olmeca olmeca	90.87	99.70	15.33	28.02
	Lu. ovallesi	96.72	99.75	28.95	33.28
	Lu. panamensis	98.38	99.78	31.35	35.18
	Lu. permira	97.02	99.33	21.23	26.25
	Lu. serrana	92.60	99.10	17.69	27.41
	Lu. shannoni	94.91	96.45	17.11	13.84
	Lu. steatopyga	92.03	99.40	17.86	29.26
	Lu. trinidadensis	85.14	98.13	10.91	22.67
	Lu. undulata	96.06	99.45	21.70	26.77
	Lu. ylephiletor	97.76	99.38	50.01	41.40
Temperate	*Lu. anthophora*	90.46	91.49	32.66	26.21
	Lu. californica	97.26	99.24	27.54	40.17
	Lu. diabolica	99.38	99.56	58.95	54.70
	Lu. stewarti	95.47	98.30	23.13	35.39
Broad-range	*Lu. texana*	99.51	99.88	71.76	65.00
	Lu. vexator	68.73	72.99	-15.79	-14.78
